# The Rb_7_Bi_3−3*x*_Sb_3*x*_Cl_16_ Family: A Fully Inorganic Solid Solution with Room‐Temperature Luminescent Members

**DOI:** 10.1002/anie.202003822

**Published:** 2020-07-02

**Authors:** Bogdan M. Benin, Kyle M. McCall, Michael Wörle, Viktoriia Morad, Marcel Aebli, Sergii Yakunin, Yevhen Shynkarenko, Maksym V. Kovalenko

**Affiliations:** ^1^ Laboratory of Inorganic Chemistry ETH Zürich 8093 Zürich Switzerland; ^2^ Laboratory for Thin Films and Photovoltaics Empa—Swiss Federal Laboratories for Materials 8600 Dübendorf Switzerland

**Keywords:** antimony, luminescence, perovskites, self-trapped excitons, solvothermal synthesis

## Abstract

Low‐dimensional ns^2^‐metal halide compounds have received immense attention for applications in solid‐state lighting, optical thermometry and thermography, and scintillation. However, these are based primarily on the combination of organic cations with toxic Pb^2+^ or unstable Sn^2+^, and a stable inorganic luminescent material has yet to be found. Here, the zero‐dimensional Rb_7_Sb_3_Cl_16_ phase, comprised of isolated [SbCl_6_]^3−^ octahedra and edge‐sharing [Sb_2_Cl_10_]^4−^ dimers, shows room‐temperature photoluminescence (RT PL) centered at 560 nm with a quantum yield of 3.8±0.2 % at 296 K (99.4 % at 77 K). The temperature‐dependent PL lifetime rivals that of previous low‐dimensional materials with a specific temperature sensitivity above 0.06 K^−1^ at RT, making it an excellent thermometric material. Utilizing both DFT and chemical substitution with Bi^3+^ in the Rb_7_Bi_3−3*x*_Sb_3*x*_Cl_16_ (*x*≤1) family, we present the edge‐shared [Sb_2_Cl_10_]^4−^ dimer as a design principle for Sb‐based luminescent materials.

## Graphical abstract

**The Rb_7_Sb_3_Cl_16_ phase**, comprised of isolated octahedra and edge‐shared dimers, demonstrates photoluminescence centered at 560 nm with a quantum yield of 3.8 % at 296 K (99.4 % at 77 K) and a specific temperature sensitivity above 0.06 K^−1^ at 300 K. Utilizing DFT and chemical substitution with Bi^3+^ in the Rb_7_Bi_3−3*x*_Sb_3*x*_Cl_16_ (*x*≤1) family, the [Sb_2_Cl_10_]^4−^ dimer is presented as a design principle for Sb‐based luminescent materials.

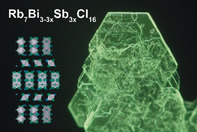

## Introduction

Low‐dimensional metal halide semiconductors, especially their zero‐dimensional (0D) counterparts,^1^ exhibit vastly different properties as compared to the 3D metal halide perovskites.^2^ While 3D metal halide perovskites have advanced the field of optoelectronics with significant improvements in full‐color imaging,^3^ photodetection,^4^ X‐ray imaging,^5^ hard‐radiation detection,^6^ solar cells,^7^ and light‐emitting diodes,^8^ in part due to their defect‐tolerant photophysics and charge transport properties,^9^ their low‐dimensional counterparts have found their niche in complementary fields such as solid‐state lighting,^10^ scintillation,^11^ and remote optical thermometry (ROT) and thermography.^12^ ROT and thermography are critical methods in several fields ranging from the diagnosis of technical failures to medical diagnostics in which thermal fluctuations or temperature deviations must be precisely identified.^13^

Many previous examples of luminescent zero‐dimensional materials have focused on Group 14 group metals (Pb^2+^, Sn^2+^, and Ge^2+^).10a, 11, 14 Of these, materials containing Sn^2+^ octahedra and disphenoids have been demonstrated to have room‐temperature (RT) photoluminescence quantum yields (PLQYs) of up to 20 % in fully inorganic cases14a and above 80 % in hybrid organic–inorganic ones.^11^, ^15^ Regardless of their performance, these materials are still hindered by their oxidative instability, which necessitates post‐processing to shield them from the environment. This challenge is exacerbated for finely dispersed forms of these materials; for example, Cs_4_SnBr_6_ NCs are stable for only several hours before significant degradation is observed.^16^

Further progress in the field of 0D optoelectronics requires the discovery of oxidatively stable and thermally robust phosphors that exhibit either of the two most useful features of their Group 14 counterparts: high RT PL from the relaxation of self‐trapped excitons (STEs) for solid‐state lighting, or temperature‐dependent lifetimes for ROT. The closest ns^2^‐analogue to the Group 14‐STE emitters are the trivalent pnictogens (Sb^3+^ and Bi^3+^).^17^ While several hybrid organic–inorganic low‐dimensional pnictogen halide phases are known,^18^ their fully inorganic counterparts, such as the A_3_B_2_X_9_ (A=Rb, Cs; X=Cl, Br, I) family of compounds and the A_2_B^I^B^III^X_6_ (A=Rb, Cs; B^I^=Na, Ag; B^III^=Sb, Bi; X=Cl) double perovskites exhibit either no, or very inefficient, RT PL.^19^

It was found that Sb^3+^‐doped and Bi^3+^‐doped structures are photoluminescent at RT, and that these are efficient STE emitters.^20^ This is true despite the higher structural dimensionality of many of these materials, as the doping provides isolated environments for the Sb^3+^ and Bi^3+^ centers, effectively reducing the electronic dimensionality to 0D.^21^ We posited that 0D structures with isolated ns^2^ centers, as observed in the hybrid organic–inorganic perovskites based on Sb^3+^,18a would be the most effective fully inorganic solid‐state emitters. We thus sought to replicate such a 0D environment through dimensional reduction of the pnictogen halide family through the exploration of alkali‐halide‐rich structures.

Herein we present the synthesis, structural tunability, and characterization of several new low‐dimensional pnictogen chlorides of the Rb_7_Bi_3*x*_Sb_3−3*x*_Cl_16_ family that exhibit RT PL. These compounds were first prepared by Wells as early as 1897,^22^ but their structures and optical properties remained undetermined until a recent report on Rb_7_Bi_3_Cl_16_ nanocrystals.^23^

## Results and Discussion

The antimony‐based Rb_7_Sb_3_Cl_16_ was synthesized solvothermally from Sb_2_O_3_ and RbCl in concentrated HCl at 160 °C. After holding this temperature for 24 h, the reaction was slowly cooled to RT at a rate of 5 °C h^−1^. The contents of the reaction were separated by vacuum filtration to yield clear, colorless hexagonal platelets as the only product.

**Figure 1:**
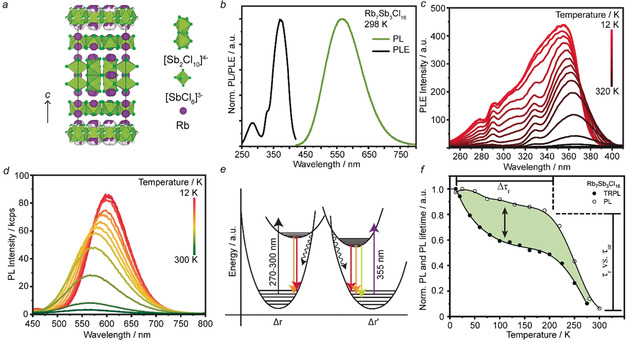
a) The crystal structure of Rb_7_Sb_3_Cl_16_ as viewed along the [100] axis contains [Sb_2_Cl_10_]^4−^ dimers and [SbCl_6_]^3−^ octahedra (green with green chlorine atoms) separated by Rb^+^ cations (purple).^31^ b) RT PL and PLE spectra for Rb_7_Sb_3_Cl_16_. c) dT‐PLE spectra of Rb_7_Sb_3_Cl_16_ measured at the PL max for each temperature. d) dT‐PL spectra for Rb_7_Sb_3_Cl_16_ measured at the PLE‐max at each temperature. e) The proposed configurational‐coordinate diagram for Rb_7_Sb_3_Cl_16_. f) A comparison of the integrated PL intensity from subpanel (d) and the average, intensity (integral) weighted, lifetime for Rb_7_Sb_3_Cl_16_.

Single‐crystal X‐ray diffraction at RT reveals that Rb_7_Sb_3_Cl_16_ crystallizes in the hexagonal space group *P* $\bar{6}$ 2*m*, with lattice parameters *a*=*b*=12.9802(4) Å, *c*=34.2522(11) Å (Figure 1 a; Supporting Information, Tables S1–S10), with powder X‐ray diffraction confirming that the product is phase pure and structurally stable over months (Supporting Information, Figures S1, S2). This quasi‐0D structure is comprised of alternating layers of isolated [Sb_2_Cl_10_]^4−^ edge‐shared dimers and [SbCl_6_]^3−^ octahedra; these layers are stacked along the *c*‐axis. Intriguingly, the [Sb_2_Cl_10_]^4−^ edge‐sharing dimer unit is distorted with a slight bend towards one side, which might be a consequence of the lone pair of the central Sb^III^ cations, as suggested by Ruck et al. for the similar compound, Tl_7_Bi_3_I_16_.^24^ Every other dimer layer exhibits threefold rotational disorder in the *a*‐*b* plane, with equal one‐third occupancy for each possible rotation providing the best structural refinement (Supporting Information, Figures S3, S4). This asymmetry also affects the [SbCl_6_]^3−^ octahedral layers adjacent to the disordered dimer layers, as the thermal parameters of the neighboring Cl atoms are significantly higher than those on faces neighboring the ordered dimer layer (Supporting Information, Figure S5). This disorder appears to be intrinsic to this compound, as cooling to 230 K or 100 K yields isostructural refinements with decreasing lattice parameters and no changes to either the rotational disorder or the unit cell ordering (Supporting Information, Figure S6; Tables S1–S10).

By obtaining single crystals of Rb_7_Sb_3_Cl_16_, we were also able to measure an absorption coefficient of about 150 cm^−1^ for these materials (Supporting Information, Figure S7). Additionally, Rb_7_Sb_3_Cl_16_ is most likely a direct band gap material and the band gap was determined to be 3.27 eV (Supporting Information, Figure S7, inset).

Exposing Rb_7_Sb_3_Cl_16_ crystals to ultraviolet (UV) light at RT results in yellow PL centered at 560 nm with a full‐width‐at‐half‐maximum (FWHM) of 0.53 eV, a RT (namely, 296 K) PLQY of 3.8±0.2 % (Figure 1 b), and a PLQY of 99.4±0.5 % at 77 K (Supporting Information, Figure S8). The drastic increase in PLQY with cooling has been observed to be a general feature of such 0D materials.^12^ Furthermore, the PLQY remains stable over the course of 2 months after storage under ambient conditions. The PL excitation (PLE) spectrum for this material exhibits a molecular‐like peak in the UV with a large Stokes shift of 1.16 eV, which is typical for 0D STE phosphors.

To further characterize these properties and gain insight into the source of emission in Rb_7_Sb_3_Cl_16_, we first conducted a series of steady‐state spectroscopic experiments: excitation‐power dependent PL (dW‐PL) at RT (Supporting Information, Figure S9), temperature‐dependent PLE (dT‐PLE) and PL (dT‐PL) from RT down to 12 K (Figure 1 c,d; Supporting Information, Figure S10).

The RT PL and PLE shown in Figure 1 b are typical for STE‐emissive 0D materials. Further evidence for this mechanism was obtained by performing power‐dependent PL measurements from an average excitation power of 2 μW to 2 mW (Supporting Information, Figure S9). The clearly linear behavior (slope=0.95, R^2^=0.998) supports the STE hypothesis, as permanent defects may be saturated at sufficiently high excitation intensities.19b This also provides a base reference for later thermometric experiments, as the excitation intensities utilized are insufficient to increase non‐radiative thermal quenching and do not cause any spectral shifting.

A clear blue‐shift in the main excitation peak is observed in the PLE as temperature decreases, along with the appearance of several additional bands (Figure 1 c). These additional peaks appear strongly overlapped and convoluted, but can be approximately placed at 270 nm, 290 nm, 305 nm, and 330 nm. As the temperature increases, these either disappear owing to quenching or merge until only the PLE max at 365 nm is recognizable. The multiple peaks at cryogenic temperatures hint at the diversity of Sb^III^ environments present within this structure, suggesting their mutual involvement in the observed spectra.

The dT‐PL spectra were then measured by exciting at each of the PLE peak positions listed above as they evolved with temperature (Figure 1 d; Supporting Information, Figure S10). Excitation at the PLE max was used to measure the PL spectra in Figure 3 d as a function of temperature. These spectra exhibit three discernable trends: PL intensity saturation below 40 K, a PL red‐shift with decreasing temperature, and PL broadening as temperature increases.

The integrated PL intensity of these spectra could be fit with an Arrhenius model containing two activation energies to investigate the quenching of each peak with temperature (Supporting Information, Figure S11). While an exact description for these energies (<30 meV and >200 meV) cannot be given, we suggest that the large activation energy is related to thermal quenching of the STE state and the <30 meV may be the activation energy that corresponds to transfer between different triplet sates of the Sb^3+^ cation, as previously determined for Cs_3_Bi_2_Cl_9_.19a

The PL red‐shift with decreasing temperature occurs concomitantly with an increasing Stokes shift (1.21 eV at RT to 1.43 eV at 12 K) and maybe the result of an altered lone‐pair stereoactivity.^25^ This same behavior is observed in the dT‐PL spectra originating from 290 nm and 330 nm excitation (Supporting Information, Figure S10).

To probe these observations, the FWHM of the three sets of dT‐PL spectra were fit with the Toyozawa model to determine the phonon energy associated with the observed temperature dependent broadening (Supporting Information, Figure S12, S13). An energy of 20.1±0.5 meV (ca. 165 cm^−1^) was extracted from the broadening of the main emission peak (ca. 360 nm excitation). The same analysis, when applied to the dT‐PL spectra at 290 nm excitation, reveals a higher effective phonon energy of 39±1 meV while the dT‐PL spectra at 330 nm excitation is discontinuous and requires two phonon energies of 19.4±0.9 meV and 38.6±0.5 meV to model, suggesting the overlap of features associated with both [Sb_2_Cl_10_]^4−^ dimers and [SbCl_6_]^3−^ octahedra (Supporting Information, Figure S13). The agreement of these energies implies that there are two distinct emission features with characteristic phonon energies near 39 meV and 20 meV; however, these effective phonon energies represent an average energy and are not always the result of a single vibrational mode. Nevertheless, they can be often correlated to the Raman spectrum of a material.

The Raman spectrum for Rb_7_Sb_3_Cl_16_ is quite complex, as there are vibrational modes from both the octahedral and dimeric units with additional peak splitting due to the bent dimers that break the *D*_2*h*_ symmetry of a perfect dimer unit (Supporting Information, Figure S14). Therefore, while a small peak at 160 cm^−1^ (19.8 meV) and the most intense peak at 306 cm^−1^ (37.9 meV) match the energies extracted from the Toyozawa fits of FWHM vs. T from the dT‐PL spectra, the large number of vibrational modes precludes any definite identification of the structural origin for these features.

These observations are further corroborated by PL‐PLE maps measured at 12 K, 100 K, and 200 K (Supporting Information, Figure S15). At the lowest temperature, a diagonal trend in the map can be observed indicating the presence of two distinct, yet overlapped, bands. As the temperature increased, these features continued to overlap and then fade until only a single peak in the PL‐PLE map could be observed.

With these observations in mind, we propose a qualitative configurational coordinate (CC) diagram for Rb_7_Sb_3_Cl_16_ (Figure 1 e). Unlike previous models for STE‐emitters, it includes two ground states and two excited states. The two separate ground states were drawn to reflect the suspected contribution of both the [Sb_2_Cl_10_]^4−^ dimers and [SbCl_6_]^3−^ octahedra, while the separate excited states are based on the existence of two strongly overlapped emission peaks at cryogenic temperatures and indicate the STEs that may form on these isolated structural features. The wavy lines that originate from the intersection of ground and excited states represent thermally activated (phonon‐assisted) quenching processes which compete with radiative recombination at temperatures near to RT.

As depicted in the CC diagram, the spectral features originating from [Sb_2_Cl_10_]^4−^ dimers and [SbCl_6_]^3−^ octahedra may have different lifetime‐temperature behaviors. Therefore, dT‐time‐resolved PL (dT‐TRPL) was measured from RT to 12 K (Figure 1 f). While RT TRPL demonstrates a monoexponential trace similar to those observed in other STE emitting systems (Supporting Information, Figure S16),^12^ the average lifetime becomes biexponential as the temperature decreases indicating the existence of two different radiative processes. This behavior occurs concomitantly with the average lifetime becoming strongly wavelength‐dependent (Supporting Information, Figure S16c).

These two observations are similarly tied to the efficient radiative recombination of STEs at both [SbCl_6_]^3−^ octahedra and [Sb_2_Cl_10_]^4−^ dimers at low temperatures. We investigated this observation further by performing a time‐resolved emission spectroscopy (TRES) experiment (Supporting Information, Figure S17). The pseudocolor 2D TRES intensity plot closely matches the RT steady state PL spectrum. Upon close inspection, a small, very fast component can be identified with very weak blue‐shifted PL further indicating that these are two non‐interacting, monoexponential processes that have spectral overlap thus supporting the hypothesis that two emissive centers may exist except that one clearly dominates in intensity.

This strong dependence of the PL lifetime on temperature has been previously demonstrated to be a general feature of other 0D and 1D Sn‐halides, and showcases such materials as exceptional thermometric agents.^12^ The outstanding suitability of such materials towards thermometry and thermography stems from three main factors: the very steep dependence of their PL lifetime on temperature, the fact that these PL lifetime values fall in an easy‐to‐measure range that spans several nanoseconds to several microseconds, and the intrinsic nature of the PL lifetime as it is only affected by temperature and remains unaltered by other factors such as impurities or partial degradation. Therefore, the average lifetime versus temperature behavior of Rb_7_Sb_3_Cl_16_ was evaluated to screen its potential thermometric performance. By comparing the integrated PL intensity with the average lifetime, two distinct temperature‐sensitive regimes can be identified: radiative‐nonradiative competition (quenching) near RT and radiative only (non‐quenching) below 100 K. The quenching regime behaves analogously to Sn‐based STE emitters, with the PL‐intensity and average lifetime varying directly owing to competition between radiative and non‐radiative channels.^12^ The non‐quenching regime demonstrates temperature‐dependent lifetimes without a significant decrease in PL intensity, indicating a direct variation in the radiative lifetime. This feature has been previously observed in Bi^III^‐based systems and is attributed to a change in the emission characteristics owing to transfer between different luminescent triplet states. In both regimes (cryogenic temperatures and close to RT), the observed lifetime shifts may be significant enough to have potential applications in ROT. Furthermore, Rb_7_Sb_3_Cl_16_ exhibits excellent thermal stability with complete reversibility in PL intensity and PL lifetime up to 150 °C (423 K; Supporting Information, Figure S18).

The figure‐of‐merit for this application is the specific thermometric sensitivity, where higher specific sensitivities indicate greater thermometric precision as smaller thermal deviations are required to alter the average PL lifetime. These can be calculated using the following formula:

(1) $$\alpha =-\frac{d\tau }{dT}\frac{1}{\tau }$$

where *α* is the specific sensitivity in inverse temperature, *τ* is the average PL lifetime, and *T* is the temperature.^12^, ^26^ The specific thermometric sensitivity for Rb_7_Sb_3_Cl_16_ is shown in the Supporting Information, Figure S19. Although the non‐quenching regime (*T*<100 K) yields rather low values for *α* (≤0.01 K^−1^), *α* exceeds 0.06 K^−1^ near RT, equaling that of the exceptional Sn‐halide‐based STE thermographic materials^12^ and highlighting the potential of this compound as a stable inorganic thermometric material.

**Figure 2:**
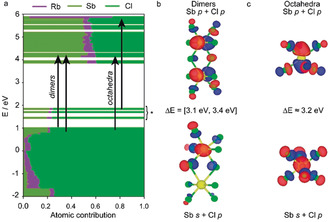
a) Partial density of states for Rb_7_Sb_3_Cl_16_. b) Occupied (lower) and unoccupied (higher) molecular orbitals belonging to [Sb_2_Cl_10_]^4−^ dimers. c) Occupied (lower) and unoccupied (higher) molecular orbitals belonging to [SbCl_6_]^3−^ octahedra.

Given this excellent thermal sensitivity, we sought to determine which structural feature ([SbCl_6_]^3−^ octahedra or [Sb_2_Cl_10_]^4−^ dimer) was responsible for, or dominant in, the observed RT PL. From the dT‐PL/PLE/TRPL and Raman experiments, we could already infer that both structural units within the Rb_7_Sb_3_Cl_16_ structure contribute to its PL. To help determine which Sb‐site in Rb_7_Sb_3_Cl_16_ can be assigned to the PL and PLE max for this material, we performed density functional theory (DFT) calculations of the partial density of states (DOS) and compared the energies of these transitions (Figure 2 a). The disorder in the complex Rb_7_Sb_3_Cl_16_ structure was unable to be modeled through a tripled supercell along the *c*‐axis, as the resulting structure was too large to utilize in computational studies. A new ordered model was therefore generated in Materials Studio from the 230 K Rb_7_Sb_3_Cl_16_ structure, maintaining the original unit cell size and using the other partially ordered layer as a template to arrange the disordered [Sb_2_Cl_10_]^4−^ dimers.

The electronic states were found to be localized on both the [SbCl_6_]^3−^ octahedra and the [Sb_2_Cl_10_]^4−^ dimers, with no states near the HOMO–LUMO gap extending over incongruent structural features (Figure 2 b,c). The states near the valence band/HOMO are comprised of Sb 5s and Cl 4p orbitals, whereas the conduction band/LUMO consists primarily of Sb 5p, Cl 4p orbitals (corresponding to an sp to p allowed transition). As in the case of other main‐group metal halides,^27^ the alkali cation (Rb^+^, purple) does not significantly contribute to the DOS near the band gap.

Unexpectedly, the DFT calculations indicated that both [Sb_2_Cl_10_]^4−^ dimers and [SbCl_6_]^3−^ octahedra had energetically feasible transitions lying close to one another. Although most low‐energy transitions belong to the [Sb_2_Cl_10_]^4−^ dimers, the HOMO is octahedral. While our model is far from definitive, it suggests that [Sb_2_Cl_10_]^4−^ dimers are the predominant source of PL and therefore that the mutual optical activity, especially at low temperatures, is likely to be the result of numerous possible transitions occurring on effectively isolated structural features.

The inability to effectively distinguish between these sites computationally inspired us to take a chemical approach and investigate the Rb_7_Bi_3−*x*_Sb_*x*_Cl_16_ family, where optical changes could, ideally, be tracked back to discrete structural modifications. Previously, it was reported that Rb_7_Bi_3_Cl_16_ exhibits blue PL when prepared as nanocrystals.^23^ Given the observation of bright STE emission from single crystals in other 0D systems as well as in Rb_7_Sb_3_Cl_16_, we expected that we would be able to study these same optical properties in single crystals of Rb_7_Bi_3_Cl_16_.

**Figure 3:**
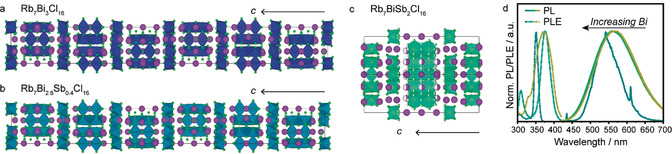
a) The crystal structure of Rb_7_Bi_3_Cl_16_ as viewed along the [100] axis contains Bi dimers and octahedra (blue with green chlorine atoms) separated by Rb^+^ cations (purple).^31^ b) The crystal structure of Rb_7_Bi_2.6_Sb_0.4_Cl_16_ as viewed along the [100] axis contains mixed Sb/Bi dimers and octahedra (cerulean with green chlorine atoms) separated by Rb^+^ cations (purple).^31^ c) The crystal structure of Rb_7_BiSb_2_Cl_16_ as viewed along [110] contains mixed Sb/Bi dimers and octahedra (aquamarine with green chlorine atoms) separated by Rb^+^ cations (purple).^31^ d) The normalized RT PL (solid) and PLE (dashed) spectra of Rb_7_Bi_3−3*x*_Sb_3*x*_Cl_16_.

Single crystals of the Rb_7_Bi_3_Cl_16_ phase were grown solvothermally from a solution containing a 1:1 ratio of Rb:Bi to avoid the formation of the Rb_3_BiCl_6_ phase. Although the structure was confirmed to have similar motifs to that of its Sb‐analogue (bismuth chloride dimers and octahedra), it was observed to have additional superstructure reflections in the diffractograms that were not fully described by the disordered model isostructural to the Sb‐endmember (Supporting Information, Figure S20; Tables S11–S14). Instead, the Rb_7_Bi_3_Cl_16_ phase crystallizes in the *R* $\bar{3}$ *c* space group, with a tripled *c*‐axis to yield unit cell parameters of *a*=*b*=13.10250(10) Å and *c*=102.9084(17) Å.(Figure 3 a; Supporting Information, Note 1).

Contrary to the Sb‐analogue, Rb_7_Bi_3_Cl_16_ did not exhibit PL under blue‐light or UV excitation at RT (Supporting Information, Figure S21). Furthermore, pattern matching the solved structure confirms the product to be phase pure Rb_7_Bi_3_Cl_16_, ruling out the possibility of any parasitic impurities such as Rb_3_BiCl_6_ that might absorb UV light without re‐emission (Supporting Information, Figures S22–S25). By cooling Rb_7_Bi_3_Cl_16_ to 12 K, red PL centered at 610 nm was observed; however, the PL rapidly quenched and red‐shifted with increasing temperature until it had completely vanished around 200 K (Supporting Information, Figures S26, S27). The observed red PL also suggests that [Bi_2_Cl_10_]^4−^ dimers, rather than [BiCl_6_]^3−^ octahedra (which should emit blue), are key to the observed luminescence.^28^ Furthermore, the PLE exhibits a much simpler spectrum than Rb_7_Sb_3_Cl_16_ with only one major peak at 355 nm and a smaller feature located around 290 nm. We attributed these two features to the bismuth chloride dimers and octahedra, respectively, and suppose that their relatively weak intensity is due to a low‐temperature quenching regime (radiative/non‐radiative competition) for this material. We confirmed this with dT‐TRPL and found that the center of the quenching regime was at about 100 K (Supporting Information, Figure S28, S29).

By characterizing the two compositional endmembers, we could see that the Bi^3+^ effectively acts as a knock‐out element for RT PL. Any changes to the RT PL and PLE spectra should therefore be related to the changing environment around Sb.

Two intermediate compositions containing both Sb and Bi were then prepared (additional synthetic information can be found in the Supporting Information). The Sb‐rich composition, Rb_7_BiSb_2_Cl_16_, was found to be isostructural with Rb_7_Sb_3_Cl_16_ with the Bi atoms forming a solid solution with partial occupancy of both the octahedra and dimer sites (Figure 3 b; Supporting Information, Figure S30, Tables S15–S18). The Bi‐rich compound, Rb_7_Bi_2.6_Sb_0.4_Cl_16_, on the other hand, exhibits a fully ordered structure isostructural to Rb_7_Bi_3_Cl_16_ (Figure 3 c; Supporting Information, Figure S31, Tables S19–S22).

These four structures, although visually dissimilar based on unit cell parameters alone, all constitute a single isostructural 7‐3‐16 structure family. The three‐fold rotational symmetry that masquerades itself as disorder in the dimer layer of the Sb‐rich and Sb‐only structures is unmasked in the Bi‐rich and Bi‐only phases. Furthermore, these materials adhere to Vegard's law (when axis tripling is accounted for) and suggest a solid solution between Sb and Bi (Supporting Information, Figure S32).

Although it had been expected that these two cations, having different sizes (0.76 Å for Sb^3+^ and 1.03 Å for Bi^3+^),^29^ might occupy different sites within these structures, the structure solutions indicated similar alloying across all sites. This was also strongly supported by solid‐state nuclear magnetic resonance (ssNMR) experiments. ^87^Rb was selected as the nuclei of choice as ^85^Rb, ^121^Sb, ^123^Sb, and ^209^Bi all possess large quadrupolar moments, and the multitude of different chlorine species (even in the compositional endmember materials) excluded ^35^Cl and ^37^Cl. The 1D experiments (Supporting Information, Figure S33) for Rb_7_Sb_3_Cl_16_ and Rb_7_Bi_3_Cl_16_ exhibit signals that are broadened by a second order quadrupole interaction. The mixed materials, however, only show broad overlapping signals. To improve the resolution, multi‐quantum magic‐angle spinning (MQMAS) experiments were used (Supporting Information, Figure S34). This technique effectively separates the anisotropic quadrupole interaction from the isotropic chemical shift thus allowing for the identification of individual species (namely, atomic environments). The mixed‐metal materials exhibit signals around 70 ppm that are spread along the chemical shift axis and are not present in the spectra of Rb_7_Sb_3_Cl_16_ or Rb_7_Bi_3_Cl_16_. This chemical shift distribution supports the formation of a solid solution owing to the stochastic distribution of Sb and Bi among all crystallographic sites.

Optically, the substitution of Sb^3+^ with Bi^3+^ quenches the emission of these materials, and blue‐shifts both the PL and PLE with respect to Rb_7_Sb_3_Cl_16_ (Figure 3 d). Concomitant to the blue‐shift of the Bi‐rich PLE spectrum, the width of the PLE peak sharply narrows. This is possibly related to the quenching of any mixed [Bi_2−*x*_Sb_2*x*_Cl_10_]^4−^ (*x*≤1) dimers as the alloying with Bi would be expected to decrease phonon energies and therefore lead to lower quenching temperatures.

These two materials were then investigated by dT‐PL/PLE/TRPL; their specific sensitivities were also determined and compared to both Rb_7_Sb_3_Cl_16_ and Rb_7_Bi_3_Cl_16_ (Supporting Information, Figures S35–S39). Analogously to Rb_7_Bi_3_Cl_16_, the PLE spectra of both Rb_7_BiSb_2_Cl_16_ and Rb_7_Bi_2.6_Sb_0.4_Cl_16_ exhibit fewer peaks vis‐à‐vis Rb_7_Sb_3_Cl_16_. Furthermore, they both feature a prominent PLE max around 355 nm and a second weaker feature around 300–320 nm (Supporting Information, Figures S35, S37). Excitation at these two sets of wavelengths results in two separate emission bands, with similar emission energies but different intensities (Supporting Information, Figures S36, S38, S39). As a result, the intensity ratios between these two sets of wavelengths are dependent on both emission wavelength and temperature (Supporting Information, Figure S40). By comparing the excitation‐peak intensity ratios with temperature, an inverse relationship between high energy and low energy transitions could be observed for the Sb‐rich phase. However, this relationship is not observed in the Bi‐rich phase, indicating that energy transfer between high and low energy transitions is unlikely, which is in agreement with the computational prediction that there are no viable dimer‐to‐octahedra transitions near the HOMO–LUMO gap for Rb_7_Sb_3_Cl_16_ (Figure 2; Supporting Information, Figure S41).^30^ A more plausible interpretation is that the higher and lower energy excitation peaks are, respectively, associated with octahedral and dimer environments.

The comparison of PL and PLE spectra of all materials at 12 K demonstrates that the mixed‐metal materials have larger band gaps than the compositional endmembers (Supporting Information, Figures S42, S43). However, the PL for both substituted materials is blue‐shifted from both Rb_7_Sb_3_Cl_16_ and Rb_7_Bi_3_Cl_16_, suggesting that they emit from a similar structural feature. By comparing the PL and PLE spectra at 200 K, we can see Rb_7_Bi_3_Cl_16_ has already quenched, indicating that any [BiCl_6_]^3−^ octahedra or [Bi_2_Cl_10_]^4−^ dimers should be optically silent. Therefore, we expect mixed‐Sb/Bi dimers to be dark as well; this leaves the Sb‐octahedra as the source of the weak and blue‐shifted emission which is observed in the mixed‐metal systems, especially the Bi‐rich Rb_7_Bi_2.6_Sb_0.4_Cl_16_. While the PLE shows that the peak position for the mixed‐metals matches that of Rb_7_Sb_3_Cl_16_ (agrees with previous PL/PLE maps), the PL demonstrates a monotonic shift in peak emission wavelength with the Bi‐rich phase having the most blue‐shifted emission as a result of weak [SbCl_6_]^3−^ octahedra emission. This agrees with our assignment of [Sb_2_Cl_10_]^4−^ dimer‐centered emission as the predominant source of PL in Rb_7_Sb_3_Cl_16_ at RT.

To judge the potential of these substituted materials as thermometric luminophores, as well to determine whether Sb/Bi alloying is an effective way to alter the thermometric sensitivity regime, the PL lifetimes of the two mixed‐metal materials were measured to determine their specific sensitivities. In both cases, the mixed‐metal compositions demonstrate lifetimes that are intermediate between those of Rb_7_Sb_3_Cl_16_ and Rb_7_Bi_3_Cl_16_ (Supporting Information, Figure S44). However, the additional Bi^3+^ in these structures appears to quench the emission without increasing the steepness of the lifetime vs. temperature curve. This widening of the quenching regime (radiative vs. nonradiative competition) decreases specific sensitivity values, showing that the Sb/Bi substitution does not lend itself towards thermographic utilization (Supporting Information, Figure S45).

Given the increased Bi content of these materials, as well as previous works on the use of low‐dimensional materials as detectors, we characterized the electronic properties and measured the X‐ray photoresponse of Rb_7_Sb_3_Cl_16_ and Rb_7_Bi_2.6_Sb_0.4_Cl_16_, which had yielded the largest crystals (Supporting Information, Figures S46–S48). As expected from their 0D structure, these materials exhibit very low conductivities (hundreds of pS cm^−1^). These materials also demonstrate a weak photocurrent response under X‐ray illumination, with Rb_7_Sb_3_Cl_16_ having a *μτ* product of 1.3×10^−5^ cm^2^ S^−1^ V^−1^ (Supporting Information, Figure S47), on par with that of the pnictogen A_3_M_2_I_9_ compounds.27b The Bi rich structure has an improved X‐ray photoconductivity with a *μτ* of 1.75×10^−3^ cm^2^ S^−1^ V^−1^ (Supporting Information, Figure S48).

As a final example of the numerous synthetic possibilities for this system, we also explored the substitution of Rb^+^ for K^+^. This resulted in the formation of a related 0D structure; however, the main product phase was KCl with only small amounts (ca. 3–5 %) of K_7_Sb_3_Cl_16_. This material was found to crystallize in a *P*6_3_/*mmc* space group, and also contains an ordered octahedral layer and a disordered dimeric layer (Supporting Information, Figures S49, S50; Table S23–26; Note S3). It exhibits RT PL centered at 590 nm with a Stokes shift of 1.2 eV and a FWHM of 0.53 eV (Supporting Information, Figure S51). This phase exhibits similar temperature dependent properties as its Rb‐analogue and is another promising thermometric luminophore with specific sensitivities similar to those of Rb_7_Sb_3_Cl_16_ (Supporting Information, Figures S52–S54).

## Conclusion

Through the synthesis and substitutional investigation of the Rb_7_Bi_3*x*_Sb_3−3*x*_Cl_16_ material family, we found three RT luminescent 0D Sb‐halides that exhibit excellent thermometric sensitivities around RT while remaining oxidatively stable. Furthermore, through a combination of DFT analysis and temperature‐dependent optical studies, we find evidence suggesting that the efficient RT PL originates from the edge‐shared [Sb_2_Cl_10_]^4−^ dimers of the structures. This structural motif has not received significant attention to date, but these results show that such dimers may be an excellent coordination to target for the discovery of luminescent 0D pnictogen‐halides.

## Conflict of interest

The authors declare no conflict of interest.

## Supporting information

As a service to our authors and readers, this journal provides supporting information supplied by the authors. Such materials are peer reviewed and may be re‐organized for online delivery, but are not copy‐edited or typeset. Technical support issues arising from supporting information (other than missing files) should be addressed to the authors.

Supplementary

Supplementary
