# Supplementary material for: The Rb7Bi3−3xSb3xCl16 Family: A Fully Inorganic Solid Solution with Room‐Temperature Luminescent Members
Source: Angew Chem Int Ed Engl. 2020 Jul 2;59(34):14490–7. doi: 10.1002/anie.202003822 (PMC7496723; doi:10.1002/anie.202003822)
Supplement: Supplementary file 2 — Supplementary [file ANIE-59-14490-s002.pdf]

Angewandte Chemie
Eine Zeitschrift der Gesellschaft Deutscher Chemiker

Supporting Information

The $Rb_7Bi_{3-3x}Sb_{3x}Cl_{16}$ Family: A Fully Inorganic Solid Solution with Room-Temperature Luminescent Members

*Bogdan M. Benin, Kyle M. McCall, Michael Wörle, Viktoriia Morad, Marcel Aebli, Sergii Yakunin, Yevhen Shynkarenko, and Maksym V. Kovalenko**

anie_202003822_sm_miscellaneous_information.pdf

Supporting Information

Table of Contents

Table of Contents 1

1. EXPERIMENTAL PROCEDURES 2

1.1 Materials 2

1.2 Synthesis of $Rb_7Sb_3Cl_{16}$ 2

1.3 Synthesis of $K_7Sb_3Cl_{16}$ 2

1.4 Synthesis of $Rb_7Bi_3Cl_{16}$ 2

1.5 Synthesis of $Rb_7Bi_{3-3x}Sb_{3x}Cl_{16}$ 2

1.6 Room-temperature absorption, photoluminescence (PL), quantum yield, and photoluminescence excitation (PLE) spectra 3

1.7 Absorption coefficient determination 3

1.8 Temperature dependent (dT) PL, dT-PLE, dT-time-resolved PL (TRPL), time-resolved emission spectroscopy (TRES), and power-dependent PL. 3

1.9 Powder diffraction and single crystal diffraction 3

1.10 Raman 3

1.11 I-V and X-ray photoconductivity 4

1.12 ^{87}Rb NMR 4

1.13 Computational details 4

2. RESULTS AND DISCUSSION 5

2.1 Structural and optical characterization 5

3. REFERENCES 52

1. EXPERIMENTAL PROCEDURES

1.1 Materials

Rubidium chloride (RbCl, 99+%) was purchased from Acros, potassium chloride (KCl, ≥99.5%) and bismuth (III) oxide (Bi_2O_3, 99.9%) were purchased from Sigma Aldrich, antimony (III) oxide (Sb_2O_3, >98%) was purchased from Fluka, and hydrochloric acid (HCl, 37%) was purchased from VWR.

All chemicals were stored and handled, and all manipulations were performed under ambient conditions. All chemicals were used as received without further purification.

Stainless steel autoclaves from Parr instruments and Amar Equipments were utilized with PTFE containers for all solvothermal syntheses.

1.2 Synthesis of $Rb_7Sb_3Cl_{16}$

In a typical synthesis, Sb_2O_3 (1.5 mmol) was dissolved in HCl (5 mL) while stirring. Once a clear solution was obtained, RbCl (7 mmol) was added directly to the Sb_2O_3/HCl solution and the autoclave was sealed and placed into a muffle furnace. The autoclave was then heated to 160 °C at 50 °C/hr and kept at this temperature for 24 hours before cooling to room temperature at a rate of 5 °C/hr. The contents of the reaction were then separated by vacuum filtration without washing.

1.3 Synthesis of $K_7Sb_3Cl_{16}$

In a typical synthesis, Sb_2O_3 (1.5 mmol) was dissolved in HCl (5 mL) while stirring. Once a clear solution was obtained, KCl (7 mmol) was added directly to the Sb_2O_3/HCl solution and the autoclave was sealed and placed into a muffle furnace. The autoclave was then heated to 160 °C at 50 °C/hr and kept at this temperature for 24 hours before cooling to room temperature at a rate of 5 °C/hr. The contents of the reaction were then separated by vacuum filtration without washing.

1.4 Synthesis of $Rb_7Bi_3Cl_{16}$

In a typical synthesis, Bi_2O_3 (1.5 mmol) was dissolved in HCl (5 mL) while stirring. Once a clear solution was obtained, RbCl (3 mmol) was added directly to the Bi_2O_3/HCl solution and the autoclave was sealed and placed into a muffle furnace. The autoclave was then heated to 160 °C at 50 °C/hr and kept at this temperature for 24 hours before cooling to room temperature at a rate of 5 °C/hr. The contents of the reaction were then separated by vacuum filtration without washing.

1.5 Synthesis of $Rb_7Bi_{3-3x}Sb_{3x}Cl_{16}$

Sb_2O_3 and Bi_2O_3 were combined in the desired ratio (total of 1.5 mmol combined) and dissolved in HCl (5 mL) while stirring. Once a clear solution was obtained, RbCl (7 mmol) was added directly to the Sb_2O_3/Bi_2O_3/HCl solution and the autoclave was sealed and placed into a muffle furnace. The autoclave was then heated to 160 °C at 50 °C/hr and kept at this temperature for 24 hours before cooling to room temperature at a rate of 2-5°C/hr (slower rates gave larger crystals). The contents of the reaction were then separated by vacuum filtration without washing.

1.6 Room-temperature absorption, photoluminescence (PL), quantum yield, and photoluminescence excitation (PLE) spectra

Diffuse reflectance spectra of microcrystalline powders were collected using a Jasco V670 spectrophotometer equipped with an integrating sphere (ILN-725). The absorption spectra were then calculated using a Kubelka-Munk transformation.

Room-temperature Photoluminescence (PL) and photoluminescence excitation (PLE) spectra were measured using a Fluorolog iHR 320 Horiba Jobin Yvon spectrofluorometer equipped with a Xe lamp and a photomultiplier tube. Samples were measured while held between quartz slides. Absolute values of the photoluminescence quantum yield (PLQY) were measured on a Quantaurus-QY C13534-11 Series spectrometer from Hamamatsu with an integrating sphere. The sample was filled in the quartz petri dish and placed in the bottom of the sphere. Excitation was scanned from 300 to 400 nm with a 10 nm step.

1.7 Absorption coefficient determination

The absorption coefficient of $Rb_7Sb_3Cl_{16}$ was measured from a single crystal, which was taped to a 1 mm round, metallic aperture. This was placed in front of the integrating sphere of a Jasco V770 spectrometer. The crystal's thickness was measured using optical microscopy in both white light and fluorescence modes from a side view of the platelet shaped crystal.

1.8 Temperature dependent (dT) PL, dT-PLE, dT-time-resolved PL (TRPL), time-resolved emission spectroscopy (TRES), and power-dependent PL.

Temperature dependent PL, PLE, and TRPL traces were measured from 320 K to 12 K on a FluoTime 300 spectrometer from PicoQuant Gmbh that was equipped with a TimeHarp 260 PICO counting unit, which was coupled with a CS204, closed cycle helium cryostat from Advanced Research Systems for temperature variation.

PL and PLE spectra were measured using a Xe lamp coupled to a monochromator. TRPL traces were recorded using a frequency tripled Nd:YAG laser (355 nm wavelength, a power density of about 0.1 mW cm^{-2}, a pulse duration of 10 ps). Scattered light from the excitation source was suppressed with a 400 nm longpass filter placed before the emission monochromator.

The average radiative lifetimes were determined as intensity (integral) weighted $\tau_{avg} = (\sum_{i=1}^{2} \tau_i^2 \cdot A_i)$ / $(\sum_{i=1}^{2} \tau_i \cdot A_i)$ where A_i and τ_i are the corresponding amplitudes and exponential decay parameters in a bi-exponential analysis.

TRES studies were performed using the above system in the TRPL configuration with a built-in TRES wizard.

Powder dependent PL studies were performed by manually varying the excitation intensity of the 355 nm laser. A range of average excitation powers from 2 µW to 2 mW was used.

1.9 Powder diffraction and single crystal diffraction

Powder diffraction patterns were collected on a STADI P diffractometer (STOE & Cie GmbH, Darmstadt, Germany) in transmission mode (Debye-Scherrer geometry). The diffractometer is equipped with a silicon strip MYTHEN 1K detector (Fa. DECTRIS) and a curved Ge (111)-monochromator ($CuK\alpha_1$, λ=1.54056 Å).

Single-crystal X-ray diffraction measurements were conducted on Bruker Smart, Bruker Smart Apex 2, Oxford Xcalibur S, and a Bruker D8 Venture each equipped with a molybdenum sealed tube X-ray source (MoKα = , λ=0.71073 Å) and graphite monochromators. Crystals were tip-mounted with paraffin oil or Lithelen high vacuum grease.

Absorption correction was performed with Multi-scan *CrysAlis PRO* 1.171.39.31d (Rigaku Oxford Diffraction, 2017). Empirical absorption correction using spherical harmonics, implemented in SCALE3 ABSPACK scaling algorithm. Data was processed and refined with *CrysAlis PRO* 1.171.39.31d (Rigaku OD, 2017), *SHELXS*,[1] XL,[1] and Olex2.[2]

1.10 Raman

Raman spectroscopy was conducted using a Thermo Scientific DXR 2 confocal Raman microscope with 455 nm laser excitation. Each sample was measured in the range of 77.6 cm^{-1} to 3773.6 cm^{-1}, no peaks were observed above 500 cm^{-1}.

1.11 I-V and X-ray photoconductivity

I-V measurements were recorded with a Keithley 236 source measurement unit. For the characterization with X-rays, the Keithley 237 source measurement unit was used to apply a bias voltage while X-rays were generated using the Mini–X Amptek X-ray tube with an accelerating voltage of 50 kV and a X-ray tube current of 50 µA.

1.12 ^{87}Rb NMR

^{87}Rb solid-state Magic Angle Spinning (MAS) NMR was measured on a 16.4 T Bruker Avance III HD spectrometer (Bruker Biospin, Fällanden, Switzerland). The instrument was equipped with a 2.5 mm double-channel solid-state probe head. The spinning frequency was set to 20 kHz. Chemical shifts were referenced to RbCl in D_2O (0.01 M).

For the 1D spectra, a one pulse experiment was used with an excitation pulse of 1 µs. Between 128 and 1024 transients were acquired with a recycle delay of 0.5 s.

The 2D multi quantum MAS (MQMAS) spectra were acquired using a Bruker 3Q MAS pulse program for odd half integer spin nuclei, using 3 pulses with full echo acquisition (mp3qdfs). The 90-degree pulse was set to 4.1 µs and 2048 transients were acquired. To cover the whole spectral range, the transmitter offset was set to 25 and 75 ppm. Variable echo build up times (15 and 60 rotor cycles) were used to detect a full echo of fast and slow relaxing species.

1.13 Computational details

Calculations were carried out at the Density Functional Theory level as implemented in the cp2k quantum chemistry code. Using the experimentally determined and disordered $Rb_7Sb_3Cl_{16}$ structure, a $P1$ unit cell with parameters $a = c = 12.9655$, $c = 34.1932$ containing 248 atoms was constructed by ordering the disordered dimer layer with the ordered layer as a template. A mixed plane-wave and Gaussian basis set approach was used to describe the wave function and electronic density, respectively. The kinetic energy cutoff of the plane-wave basis was set to 400 Rydberg, while a double-ζ basis set plus polarization functions was employed to describe the molecular orbitals. Density of states, emission and excitation energies were calculated using Perdew-Burke-Ernzerhof (PBE) exchange-correlation functional. Scalar relativistic effects have been accounted for by using effective core potential functions in the basis set. Spin-orbit coupling effects were not included. Unit cell parameters were taken from experimental data and not relaxed, whereas atomic coordinates were optimized until the force reached 0.023 eV/ Å.

2. RESULTS AND DISCUSSION

2.1 Structural and optical characterization

Table S1. Crystal data and structure refinement comparison for $Rb_7Sb_3Cl_{16}$ at 300 K, 230 K, and 100 K.

Empirical formula	$Rb_7Sb_3Cl_{16}$	$Rb_7Sb_3Cl_{16}$	$Rb_7Sb_3Cl_{16}$
Formula weight	1530.74	1530.74	1530.74
Temperature	**300 K[a]**	**230(2) K[b]**	**100(2) K[c]**
Wavelength	0.71073 Å	0.71073 Å	0.71073 Å
Crystal system	Hexagonal	Hexagonal	Hexagonal
Space group	*P*-62*m*	*P*-62*m*	*P*-62*m*
Unit cell dimensions	a = 12.9802(4) Å, α = 90°<br>b = 12.9802(4) Å, β = 90°<br>c = 34.2522(11) Å, γ = 120°	a = 12.9655(2) Å, α = 90°<br>b = 12.9655(2) Å, β = 90°<br>c = 34.1932(8) Å, γ = 120°	a = 12.8570(2) Å, α = 90°<br>b = 12.8570(2) Å, β = 90°<br>c = 34.1574(5) Å, γ = 120°
Volume	4997.8(3) $Å^3$	4977.93(19) $Å^3$	4889.84(17) $Å^3$
Z	6	6	6
Density (calculated)	3.052 g/cm^3	3.064 g/cm^3	3.119 g/cm^3
Absorption coefficient	13.855 mm^{-1}	13.910 mm^{-1}	14.161 mm^{-1}
F(000)	4104	4104	4104
Crystal size	0.241 x 0.187 x 0.03 mm^3	0.232 x 0.214 x 0.034 mm^3	0.201 x 0.168 x 0. 084 mm^3
θ range for data collection	1.784 to 31.572°	1.787 to 30.507°	1.789 to 30.497°
Index ranges	-18<=h<=18, -18<=k<=18, -49<=l<=49	-18<=h<=18, -18<=k<=18, -48<=l<=48	-18<=h<=18, -18<=k<=18, -50<=l<=50
Reflections collected	59312	297301	251406
Independent reflections	5798 [R_{int} = 0.0908]	5524 [R_{int} = 0.1190]	5426 [R_{int} = 0.1172]
Completeness to θ = 25.242°	99.9%	99.9%	99.9%
Refinement method	Full-matrix least-squares on F^2	Full-matrix least-squares on F^2	Full-matrix least-squares on F^2
Data / restraints / parameters	5798 / 0 / 218	5524 / 0 / 218	5426 / 0 / 208
Goodness-of-fit	1.093	1.216	1.109
Final R indices [I > 2σ(I)]	R_{obs} = 0.0818, wR_{obs} = 0.2226	R_{obs} = 0.0546, wR_{obs} = 0.1492	R_{obs} = 0.0802, wR_{obs} = 0.2347
R indices [all data]	R_{all} = 0.1037, wR_{all} = 0.2385	R_{all} = 0.0559, wR_{all} = 0.1505	R_{all} = 0.0825, wR_{all} = 0.2370
Largest diff. peak and hole	6.468 and -2.562 e·$Å^{-3}$	2.429 and -1.820 e·$Å^{-3}$	3.967 and -3.597 e·$Å^{-3}$
Absolute structure	Refined as inversion twin	Refined as inversion twin	Refined as inversion twin
Absolute structure parameter	0.46(4)	0.50(3)	0.50(3)

[a] $R = \Sigma||F_o|-|F_c|| / \Sigma|F_o|$, $wR = \{\Sigma[w(|F_o|^2 - |F_c|^2)^2] / \Sigma[w(|F_o|^4)]\}^{1/2}$ and $w=1/[\sigma^2(Fo^2)+(0.1415P)^2+17.7772P]$ where $P=(Fo^2+2Fc^2)/3$

[b] $R = \Sigma||F_o|-|F_c|| / \Sigma|F_o|$, $wR = \{\Sigma[w(|F_o|^2 - |F_c|^2)^2] / \Sigma[w(|F_o|^4)]\}^{1/2}$ and $w=1/[\sigma^2(Fo^2)+(0.0623P)^2+31.5830P]$ where $P=(Fo^2+2Fc^2)/3$

[c] $R = \Sigma||F_o|-|F_c|| / \Sigma|F_o|$, $wR = \{\Sigma[w(|F_o|^2 - |F_c|^2)^2] / \Sigma[w(|F_o|^4)]\}^{1/2}$ and $w=1/[\sigma^2(Fo^2)+(0.1214P)^2+132.1359P]$ where $P=(Fo^2+2Fc^2)/3$

Table S2. Atomic coordinates (x10[4]) and equivalent isotropic displacement parameters (Å^{2}x10^3) for $Rb_7Sb_3Cl_{16}$ at 300 K with estimated standard deviations in parentheses.

Label	x	y	z	Occupancy	U_{eq}*
Sb(1)	-6677(1)	0	-656(1)	1	26(1)
Sb(2)	-13257(1)	0	-2499(1)	1	26(1)
Sb(4)	-10000	0	-4340(1)	1	23(1)
Sb(3)	-16666.67	-3333.33	-4337(1)	1	24(1)
Rb(2)	-3333.33	3333.33	0	1	34(1)
Rb(3)	-13333.33	3333.33	-1328(1)	1	35(1)
Rb(1)	-2789(2)	0	0	1	32(1)
Rb(4)	-12882(2)	0	-1309(1)	1	39(1)
Rb(7)	-10000	0	-2909(2)	1	49(1)
Rb(5)	-13333.33	3333.33	-2805(1)	1	62(1)
Rb(6)	-16661(2)	0	-2152(2)	1	65(1)
Rb(8)	-13334(2)	-3334(2)	-3674(2)	0.6667	32(1)
Rb(11)	-13307(3)	-3307(3)	-5000	0.6667	38(2)
Cl(4)	-5542(3)	2370(4)	-664(1)	1	33(1)
Cl(7)	-13047(5)	1737(5)	-2013(2)	1	51(2)
Cl(6)	-11593(5)	0	-2123(2)	1	54(2)
Cl(1)	-5456(6)	0	-1230(2)	1	66(2)
Rb(10)	-13813(5)	0	-3674(2)	0.3333	34(2)
Rb(9)	-13403(4)	-488(4)	-3688(2)	0.3333	34(1)
Cl(3)	-8076(7)	0	0	1	64(3)
Cl(2)	-8092(6)	0	-1146(3)	1	68(2)
Cl(9)	-13319(6)	-1645(7)	-2944(2)	1	79(2)
Cl(5)	-5231(8)	0	0	1	81(4)
Cl(8)	-15037(7)	0	-2916(3)	1	107(4)
Cl(16)	-15479(11)	-1018(10)	-4337(3)	0.3333	34(2)
Cl(12)	-12344(9)	-1087(10)	-4332(3)	0.3333	31(2)
Cl(13)	-14287(10)	-2113(12)	-4329(3)	0.3333	35(2)
Rb(12)	-13370(5)	-578(5)	-5000	0.3333	36(2)
Rb(13)	-13845(6)	0	-5000	0.3333	36(2)
Cl(14)	-15445(14)	-2074(15)	-3802(6)	0.3333	71(5)
Cl(15)	-16666(9)	-1872(14)	-3900(6)	0.3333	66(5)
Cl(18)	-15270(20)	-3370(14)	-5000	0.3333	88(11)
Cl(11)	-11453(14)	-1453(14)	-3870(6)	0.3333	49(4)
Cl(10)	-11241(13)	0	-3824(6)	0.3333	53(5)
Cl(19)	-11480(20)	-1480(20)	-5000	0.3333	104(18)
Cl(20)	-11390(20)	0	-5000	0.3333	87(14)
Cl(17)	-16590(20)	-4760(20)	-5000	0.3333	102(13)

*U_{eq} is defined as one third of the trace of the orthogonalized U_{ij} tensor.

Table S3. Anisotropic displacement parameters (Å^{2}x10^3) for $Rb_7Sb_3Cl_{16}$ at 300 K with estimated standard deviations in parentheses.

Label	U_{11}	U_{22}	U_{33}	U_{12}	U_{13}	U_{23}
Sb(1)	28(1)	28(1)	20(1)	14(1)	-1(1)	0
Sb(2)	26(1)	29(1)	23(1)	15(1)	1(1)	0
Sb(4)	24(1)	24(1)	22(1)	12(1)	0	0
Sb(3)	23(1)	23(1)	27(1)	11(1)	0	0
Rb(2)	40(2)	40(2)	21(2)	20(1)	0	0
Rb(3)	44(1)	44(1)	18(1)	22(1)	0	0
Rb(1)	32(1)	30(2)	35(2)	15(1)	0	0
Rb(4)	52(2)	37(2)	24(1)	18(1)	0(1)	0
Rb(7)	58(2)	58(2)	30(2)	29(1)	0	0
Rb(5)	69(2)	69(2)	47(2)	35(1)	0	0
Rb(6)	60(2)	37(2)	88(3)	19(1)	-2(2)	0
Rb(8)	31(2)	31(2)	32(2)	14(2)	-2(1)	-2(1)
Rb(11)	34(2)	34(2)	42(3)	16(2)	0	0
Cl(4)	33(2)	34(2)	29(2)	14(2)	0(2)	2(2)
Cl(7)	91(3)	46(2)	32(2)	45(2)	11(2)	-1(2)
Cl(6)	51(2)	104(5)	25(2)	52(3)	-1(2)	0
Cl(1)	70(3)	95(6)	41(3)	47(3)	17(2)	0
Rb(10)	37(2)	24(3)	37(3)	12(2)	-3(2)	0
Rb(9)	30(2)	34(2)	37(2)	16(2)	-2(2)	2(2)

Cl(3)	36(3)	33(4)	121(9)	16(2)	0	0
Cl(2)	59(3)	39(3)	99(5)	20(2)	-46(4)	0
Cl(9)	132(6)	70(4)	68(3)	74(4)	-11(3)	-32(3)
Cl(5)	52(4)	112(10)	100(8)	56(5)	0	0
Cl(8)	71(3)	202(12)	93(6)	101(6)	-20(4)	0
Cl(16)	37(5)	32(5)	34(4)	19(4)	-2(4)	-3(4)
Cl(12)	25(4)	25(5)	35(4)	6(4)	0(4)	0(4)
Cl(13)	25(4)	44(6)	35(4)	18(5)	0(4)	-5(5)
Rb(12)	27(3)	25(3)	52(4)	11(2)	0	0
Rb(13)	26(3)	28(4)	53(5)	14(2)	0	0
Cl(14)	52(8)	58(8)	100(13)	27(8)	-40(9)	-39(9)
Cl(15)	29(6)	50(8)	109(13)	13(5)	7(6)	-44(9)
Cl(18)	54(13)	19(8)	190(30)	19(8)	0	0
Cl(11)	44(6)	44(6)	66(11)	27(7)	20(8)	20(8)
Cl(10)	39(6)	60(11)	67(12)	30(6)	19(7)	0
Cl(19)	23(8)	23(8)	270(60)	13(9)	0	0
Cl(20)	32(9)	45(16)	190(50)	22(8)	0	0
Cl(17)	68(17)	34(10)	210(40)	29(11)	0	0

The anisotropic displacement factor exponent takes the form: $-2\pi^2[h^2a^{*2}U_{11} + ... + 2hka^*b^*U_{12}]$

Table S4. Selected bond lengths [Å] for $Rb_7Sb_3Cl_{16}$ at 300 K with estimated standard deviations in parentheses.

Label	Distances
Sb(1)-Cl(4)	2.665(4)
Sb(1)-Cl(1)	2.525(6)
Sb(1)-Cl(3)	2.890(6)
Sb(1)-Cl(2)	2.487(6)
Sb(2)-Rb(4)	4.105(2)
Sb(2)-Cl(7)	2.705(5)
Sb(2)-Cl(6)	2.515(7)
Sb(2)-Rb(10)	4.090(6)
Sb(2)-Rb(9)	4.113(5)
Sb(2)-Cl(9)	2.591(6)
Sb(2)-Cl(8)	2.715(9)
Sb(4)-Cl(12)	2.638(10)
Sb(4)-Cl(11)	2.479(17)
Sb(4)-Cl(10)	2.390(17)
Sb(3)-Cl(16)	2.603(11)
Sb(3)-Cl(13)	2.675(11)
Sb(3)-Cl(14)	2.440(15)
Sb(3)-Cl(15)	2.417(13)
Rb(2)-Rb(1)	4.7199(16)
Rb(2)-Cl(4)	3.371(3)
Rb(2)-Cl(5)	3.7590(8)
Rb(3)-Rb(4)	4.6477(15)
Rb(3)-Cl(7)	3.273(5)
Rb(1)-Cl(5)	3.170(10)
Rb(4)-Cl(7)	3.379(5)
Rb(4)-Cl(6)	3.252(6)
Rb(7)-Cl(6)	3.395(6)
Rb(7)-Cl(9)	3.733(6)
Rb(7)-Cl(10)	3.52(2)
Rb(5)-Cl(7)	3.545(5)
Rb(5)-Cl(8)	3.7665(9)
Rb(6)-Cl(8)	3.360(11)
Rb(8)-Cl(9)	3.321(6)
Rb(8)-Cl(12)	3.387(11)
Rb(8)-Cl(13)	3.322(12)
Rb(8)-Cl(11)	2.531(18)
Rb(11)-Cl(13)	3.357(11)
Rb(11)-Cl(18)	2.51(3)
Rb(11)-Cl(19)	2.37(3)

Rb(10)-Rb(9)	1.011(8)
Rb(10)-Cl(8)	3.046(12)
Rb(10)-Cl(16)	2.954(12)
Rb(10)-Cl(13)	3.353(12)
Rb(10)-Cl(14)	2.495(16)
Rb(10)-Cl(15)	3.349(13)
Rb(10)-Cl(10)	3.377(18)
Rb(9)-Cl(9)	2.990(9)
Rb(9)-Cl(16)	3.290(12)
Rb(9)-Cl(12)	2.901(11)
Rb(9)-Cl(13)	2.857(12)
Rb(9)-Cl(14)	2.441(16)
Rb(9)-Cl(11)	3.396(7)
Rb(9)-Cl(10)	2.591(17)
Cl(16)-Rb(12)	3.378(12)
Cl(16)-Rb(13)	2.931(11)
Cl(16)-Cl(14)	2.30(2)
Cl(16)-Cl(15)	2.03(2)
Cl(12)-Cl(13)	2.185(17)
Cl(12)-Rb(12)	2.886(11)
Cl(12)-Cl(11)	2.147(18)
Cl(12)-Cl(10)	2.25(2)
Cl(13)-Rb(12)	2.880(11)
Cl(13)-Rb(13)	3.399(12)
Cl(13)-Cl(14)	2.37(2)
Rb(12)-Rb(13)	1.186(10)
Rb(12)-Cl(18)	3.205(18)
Rb(12)-Cl(19)	3.203(8)
Rb(12)-Cl(20)	2.28(3)
Rb(13)-Cl(20)	3.18(3)
Cl(14)-Cl(15)	1.76(2)
Cl(18)-Cl(17)	1.76(3)
Cl(11)-Cl(10)	1.772(13)
Cl(19)-Cl(20)	1.869(18)

Table S5. Atomic coordinates (x10^4) and equivalent isotropic displacement parameters (Å^{2}x10^3) for $Rb_7Sb_3Cl_{16}$ at 230(2) K with estimated standard deviations in parentheses.

Label	x	y	z	Occupancy	U_{eq}^*
Sb(1)	6676(1)	0	5657(1)	1	26(1)
Sb(2)	10000	3252(1)	7500(1)	1	24(1)
Sb(3)	10000	0	9342(1)	1	20(1)
Sb(4)	6666.67	3333.33	9340(1)	1	20(1)
Rb(1)	2786(2)	0	5000	1	26(1)
Rb(2)	6666.67	3333.33	5000	1	31(1)
Rb(3)	6666.67	3333.33	6328(1)	1	28(1)
Rb(4)	10000	2884(2)	6310(1)	1	31(1)
Rb(5)	10000	0	7912(1)	1	40(1)
Rb(6)	6666.67	3333.33	7806(1)	1	53(1)
Rb(7)	10000	6674(2)	7154(2)	1	64(1)
Rb(8)	10000	6661(2)	8677(1)	0.6667	33(1)
Rb(9)	10000	3833(4)	8678(2)	0.3333	32(1)
Rb(10)	9532(3)	2940(4)	8693(1)	0.3333	33(1)
Rb(11)	10000	3848(5)	10000	0.3333	33(2)
Rb(12)	9418(4)	2789(4)	10000	0.3333	33(1)
Rb(13)	10000	6696(3)	10000	0.6667	32(1)
Cl(1)	7909(2)	2371(2)	5663(1)	1	27(1)
Cl(2)	5457(4)	0	6230(2)	1	52(2)

Cl(3)	8109(4)	0	6134(2)	1	55(2)
Cl(4)	8077(5)	0	5000	1	58(2)
Cl(5)	5212(5)	0	5000	1	75(3)
Cl(6)	8255(3)	3032(4)	7015(1)	1	41(1)
Cl(7)	10000	1586(3)	7123(2)	1	43(1)
Cl(8)	8359(6)	1671(5)	7951(2)	1	83(2)
Cl(9)	10000	5053(6)	7923(2)	1	115(4)
Cl(10)	7868(12)	3368(10)	8770(4)	0.3333	55(3)
Cl(11)	8117(10)	4787(10)	8880(5)	0.3333	64(4)
Cl(12)	8555(10)	0	8845(4)	0.3333	39(2)
Cl(13)	10000	1237(10)	8801(4)	0.3333	48(3)
Cl(14)	8912(8)	1255(8)	9331(2)	0.3333	32(2)
Cl(15)	7894(9)	2189(8)	9329(2)	0.3333	34(2)
Cl(16)	8991(7)	4474(8)	9341(2)	0.3333	33(2)
Cl(17)	8063(14)	4704(14)	10000	0.3333	63(5)
Cl(18)	8156(14)	3404(16)	10000	0.3333	78(7)
Cl(19)	10000	8532(16)	10000	0.3333	65(7)
Cl(20)	8600(16)	8600(16)	10000	0.3333	67(8)

*U_{eq} is defined as one third of the trace of the orthogonalized U_{ij} tensor.

Table S6. Anisotropic displacement parameters (Å^{2}x10^3) for $Rb_7Sb_3Cl_{16}$ at 230(2) K with estimated standard deviations in parentheses.

Label	U_{11}	U_{22}	U_{33}	U_{12}	U_{13}	U_{23}
Label	U_{11}	U_{22}	U_{33}	U_{12}	U_{13}	U_{23}
Sb(1)	29(1)	28(1)	21(1)	14(1)	-1(1)	0
Sb(2)	28(1)	23(1)	23(1)	14(1)	0	1(1)
Sb(3)	19(1)	19(1)	21(1)	10(1)	0	0
Sb(4)	18(1)	18(1)	25(1)	9(1)	0	0
Rb(1)	22(1)	24(1)	33(1)	12(1)	0	0
Rb(2)	35(1)	35(1)	23(2)	17(1)	0	0
Rb(3)	32(1)	32(1)	19(1)	16(1)	0	0
Rb(4)	28(1)	37(1)	25(1)	14(1)	0	0(1)
Rb(5)	47(1)	47(1)	28(2)	23(1)	0	0
Rb(6)	59(1)	59(1)	42(2)	30(1)	0	0
Rb(7)	34(1)	56(2)	95(2)	17(1)	0	-4(2)
Rb(8)	35(2)	35(1)	30(2)	18(1)	0	1(1)
Rb(9)	27(2)	34(2)	34(2)	14(1)	0	1(2)
Rb(10)	36(2)	41(2)	30(2)	25(2)	-4(2)	-5(2)
Rb(11)	25(3)	26(2)	47(4)	12(2)	0	0
Rb(12)	27(2)	27(2)	46(3)	14(2)	0	0
Rb(13)	34(2)	30(2)	32(2)	17(1)	0	0
Cl(1)	31(2)	27(2)	26(1)	15(1)	-2(1)	0(1)
Cl(2)	49(2)	66(3)	48(3)	33(2)	16(2)	0
Cl(3)	45(2)	31(2)	85(4)	16(1)	-36(2)	0
Cl(4)	29(2)	24(3)	119(7)	12(2)	0	0
Cl(5)	39(3)	89(7)	113(8)	45(3)	0	0
Cl(6)	36(2)	68(2)	29(2)	34(2)	-1(2)	10(2)
Cl(7)	77(3)	38(2)	26(2)	39(2)	0	-4(2)
Cl(8)	71(3)	56(2)	75(3)	-5(2)	31(3)	20(2)
Cl(9)	218(11)	72(3)	103(5)	109(6)	0	-15(3)
Cl(10)	56(6)	50(6)	58(6)	25(5)	21(5)	0(5)
Cl(11)	41(5)	47(6)	117(11)	31(5)	35(6)	46(7)
Cl(12)	34(4)	28(5)	51(6)	14(2)	-10(5)	0
Cl(13)	64(9)	39(4)	48(7)	32(5)	0	8(5)
Cl(14)	26(4)	32(4)	38(3)	14(3)	2(3)	3(3)
Cl(15)	40(4)	28(4)	40(4)	21(3)	2(4)	-1(3)
Cl(16)	30(4)	30(4)	38(4)	13(3)	6(3)	2(3)
Cl(17)	35(7)	38(7)	128(17)	27(6)	0	0
Cl(18)	30(7)	48(9)	160(20)	25(6)	0	0
Cl(19)	22(7)	29(6)	140(20)	11(4)	0	0

The anisotropic displacement factor exponent takes the form: $-2\pi^2[h^2a^{*2}U_{11} + ... + 2hka^*b^*U_{12}]$.

Table S7. Selected bond lengths [Å] for $Rb_7Sb_3Cl_{16}$ at 230(2) K with estimated standard deviations in parentheses.

Label	Distances
Sb(1)-Cl(1)	2.664(3)
Sb(1)-Cl(2)	2.517(5)
Sb(1)-Cl(3)	2.471(5)
Sb(1)-Cl(4)	2.890(5)
Sb(1)-Cl(5)	2.942(5)
Sb(2)-Rb(4)	4.0967(19)
Sb(2)-Rb(9)	4.095(5)
Sb(2)-Rb(10)	4.112(4)
Sb(2)-Cl(6)	2.703(3)
Sb(2)-Cl(7)	2.515(4)
Sb(2)-Cl(8)	2.597(5)
Sb(2)-Cl(9)	2.745(7)
Sb(3)-Cl(12)	2.528(13)
Sb(3)-Cl(13)	2.448(13)
Sb(3)-Cl(14)	2.634(8)
Sb(4)-Cl(10)	2.481(11)
Sb(4)-Cl(11)	2.454(11)
Sb(4)-Cl(15)	2.664(8)
Sb(4)-Cl(16)	2.610(8)
Rb(1)-Cl(5)	3.145(7)
Rb(2)-Rb(3)	4.5402(18)
Rb(2)-Cl(1)	3.362(2)
Rb(2)-Cl(5)	3.7529(6)
Rb(3)-Rb(4)	4.6406(10)
Rb(3)-Cl(1)	3.365(3)
Rb(3)-Cl(2)	3.8041(9)
Rb(3)-Cl(6)	3.274(3)
Rb(4)-Cl(1)	3.299(3)
Rb(4)-Cl(3)	3.3456(17)
Rb(4)-Cl(6)	3.375(3)
Rb(4)-Cl(7)	3.250(4)
Rb(5)-Cl(7)	3.391(5)
Rb(5)-Cl(8)	3.722(6)
Rb(5)-Cl(12)	3.701(15)
Rb(5)-Cl(13)	3.437(15)
Rb(6)-Cl(6)	3.536(4)
Rb(6)-Cl(8)	3.799(6)
Rb(6)-Cl(9)	3.7648(10)
Rb(6)-Cl(10)	3.638(14)
Rb(7)-Cl(9)	3.367(9)
Rb(8)-Cl(9)	3.316(8)
Rb(8)-Cl(11)	2.533(11)
Rb(8)-Cl(16)	3.348(9)
Rb(9)-Rb(10)	1.005(6)
Rb(9)-Cl(9)	3.027(10)
Rb(9)-Cl(10)	2.537(13)
Rb(9)-Cl(11)	3.315(10)
Rb(9)-Cl(13)	3.393(14)
Rb(9)-Cl(15)	3.339(10)
Rb(9)-Cl(16)	2.939(9)
Rb(10)-Cl(8)	2.991(7)
Rb(10)-Cl(10)	2.497(14)
Rb(10)-Cl(12)	3.402(5)
Rb(10)-Cl(13)	2.592(13)
Rb(10)-Cl(14)	2.903(9)
Rb(10)-Cl(15)	2.852(9)
Rb(10)-Cl(16)	3.280(9)

Rb(11)-Rb(12)	1.191(8)
Rb(11)-Cl(16)	2.917(9)
Rb(11)-Cl(17)	3.214(13)
Rb(11)-Cl(18)	2.161(16)
Rb(12)-Cl(14)	2.882(9)
Rb(12)-Cl(15)	2.869(9)
Rb(12)-Cl(18)	2.149(16)
Rb(13)-Cl(16)	3.365(9)
Rb(13)-Cl(17)	2.548(17)
Rb(13)-Cl(19)	2.38(2)
Cl(10)-Cl(11)	1.743(16)
Cl(10)-Cl(15)	2.460(16)
Cl(10)-Cl(16)	2.429(16)
Cl(11)-Cl(16)	2.097(16)
Cl(12)-Cl(13)	1.761(10)
Cl(12)-Cl(14)	2.207(14)
Cl(13)-Cl(14)	2.306(15)
Cl(14)-Cl(15)	2.192(12)
Cl(17)-Cl(18)	1.75(2)
Cl(19)-Cl(20)	1.861(13)
Sb(1)-Cl(1)	2.664(3)

Table S8. Atomic coordinates (x10^4) and equivalent isotropic displacement parameters (Å^{2}x10^3) for $Rb_7Sb_3Cl_{16}$ at 100(2) K with estimated standard deviations in parentheses.

Label	x	y	z	Occupancy	U_{eq}*
Sb(1)	3303(2)	10000	5663(1)	1	15(1)
Sb(2)	0	6743(1)	7500(1)	1	15(1)
Sb(3)	3333.33	6666.67	9331(1)	1	12(1)
Sb(4)	0	10000	9337(1)	1	13(1)
Rb(1)	7196(2)	10000	5000	1	16(1)
Rb(2)	3333.33	6666.67	5000	1	16(1)
Rb(3)	-3333.33	3333.33	6325(1)	1	17(1)
Rb(4)	0	7115(2)	6318(1)	1	20(1)
Rb(5)	-3333.33	3333.33	7800(1)	1	34(1)
Rb(6)	-3349(2)	6651(2)	7156(2)	1	40(1)
Rb(7)	0	10000	7913(2)	1	26(1)
Rb(8)	0	6187(5)	8673(2)	0.3333	15(2)
Rb(9)	475(4)	7082(5)	8688(2)	0.3333	16(1)
Rb(10)	3339(2)	10000	8675(1)	0.6667	13(1)
Rb(11)	3312(3)	10000	10000	0.6667	12(1)
Rb(12)	6175(6)	10000	10000	0.3333	15(2)
Rb(13)	7184(6)	10551(5)	10000	0.3333	15(2)
Cl(1)	4735(9)	10000	5000	1	70(5)
Cl(2)	1890(9)	10000	5000	1	95(8)
Cl(3)	2079(4)	7616(4)	5666(1)	1	17(1)
Cl(4)	4546(7)	10000	6228(3)	1	41(2)
Cl(5)	1912(7)	10000	6164(3)	1	54(3)
Cl(6)	0	8423(6)	7131(2)	1	40(2)
Cl(7)	-1765(5)	5212(5)	7011(2)	1	32(1)
Cl(8)	0	4942(7)	7918(3)	1	98(5)
Cl(9)	1651(8)	8342(7)	7949(2)	1	71(3)
Cl(10)	0	8745(14)	8805(7)	0.3333	32(4)
Cl(11)	1467(14)	10000	8823(6)	0.3333	26(3)
Cl(12)	1112(10)	8736(9)	9333(3)	0.3333	14(2)
Cl(13)	2078(10)	7825(10)	9329(3)	0.3333	14(2)
Cl(14)	3353(11)	8128(18)	8854(7)	0.3333	54(6)
Cl(15)	4522(17)	7899(16)	8771(6)	0.3333	45(4)
Cl(16)	4537(10)	9028(9)	9344(3)	0.3333	15(2)
Cl(17)	6599(19)	11883(19)	10105(5)	0.1667	15(4)
Cl(18)	5220(20)	11890(20)	9915(7)	0.1667	27(6)
Cl(19)	1490(30)	10000	10000	0.3333	48(8)

*U_{eq} is defined as one third of the trace of the orthogonalized U_{ij} tensor.

Table S9. Anisotropic displacement parameters ($Å^2x10^3$) for $Rb_7Sb_3Cl_{16}$ at 100(2) K with estimated standard deviations in parentheses.

Label	U_{11}	U_{22}	U_{33}	U_{12}	U_{13}	U_{23}
Sb(1)	18(1)	17(1)	9(1)	8(1)	0(1)	0
Sb(2)	18(1)	16(1)	12(1)	9(1)	0	0(1)
Sb(3)	12(1)	12(1)	13(1)	6(1)	0	0
Sb(4)	14(1)	14(1)	10(1)	7(1)	0	0
Rb(1)	20(1)	18(2)	8(1)	9(1)	0	0
Rb(2)	23(2)	23(2)	2(2)	12(1)	0	0
Rb(3)	21(1)	21(1)	8(1)	11(1)	0	0
Rb(4)	20(1)	28(1)	8(1)	10(1)	0	0(1)
Rb(5)	44(2)	44(2)	13(2)	22(1)	0	0
Rb(6)	24(1)	24(1)	75(3)	14(2)	-4(2)	-4(2)
Rb(7)	32(2)	32(2)	15(2)	16(1)	0	0
Rb(8)	14(3)	19(2)	11(2)	7(2)	0	-1(2)
Rb(9)	17(2)	23(2)	15(2)	15(2)	3(2)	3(2)
Rb(10)	14(2)	13(2)	12(2)	6(1)	-1(1)	0
Rb(11)	8(2)	10(2)	20(2)	5(1)	0	0
Rb(12)	8(2)	5(3)	30(4)	2(2)	0	0
Rb(13)	10(2)	12(3)	23(3)	6(2)	0	0
Cl(1)	27(4)	37(6)	150(17)	18(3)	0	0
Cl(2)	20(4)	12(4)	250(30)	6(2)	0	0
Cl(3)	25(2)	22(2)	6(2)	14(2)	1(2)	1(2)
Cl(4)	38(3)	37(4)	47(4)	18(2)	-15(3)	0
Cl(5)	37(3)	22(3)	97(7)	11(2)	42(4)	0
Cl(6)	93(6)	39(2)	7(2)	47(3)	0	0(2)
Cl(7)	32(2)	30(2)	9(2)	-2(2)	2(2)	-4(2)
Cl(8)	207(17)	57(4)	80(7)	104(8)	0	18(4)
Cl(9)	56(4)	46(4)	58(4)	-14(3)	-31(4)	-19(3)
Cl(10)	36(10)	21(6)	45(11)	18(5)	0	-3(6)
Cl(11)	20(5)	30(9)	31(8)	15(4)	7(6)	0
Cl(12)	11(4)	8(4)	21(4)	5(3)	1(3)	2(3)
Cl(13)	12(4)	10(4)	25(4)	9(3)	-1(4)	-1(4)
Cl(14)	6(5)	46(10)	100(16)	7(6)	-10(6)	25(10)
Cl(15)	38(9)	28(7)	72(12)	17(7)	20(9)	21(8)
Cl(16)	13(4)	7(4)	25(5)	5(4)	0(4)	1(3)

The anisotropic displacement factor exponent takes the form: $-2\pi^2[h^2a^{*2}U_{11} + ... + 2hka^*b^*U_{12}]$.

Table S10. Selected bond lengths [Å] for $Rb_7Sb_3Cl_{16}$ at 100(2) K with estimated standard deviations in parentheses.

Label	Distances
Sb(1)-Cl(3)	2.655(4)
Sb(1)-Cl(4)	2.506(8)
Sb(1)-Cl(5)	2.474(8)
Sb(2)-Rb(4)	4.068(3)
Sb(2)-Rb(8)	4.068(6)
Sb(2)-Rb(9)	4.094(5)
Sb(2)-Cl(6)	2.503(7)
Sb(2)-Cl(7)	2.710(5)
Sb(2)-Cl(8)	2.721(9)
Sb(2)-Cl(9)	2.593(7)
Sb(3)-Cl(14)	2.477(19)
Sb(3)-Cl(15)	2.466(18)
Sb(3)-Cl(16)	2.630(10)
Sb(4)-Cl(10)	2.43(2)
Sb(4)-Cl(11)	2.579(18)
Sb(4)-Cl(12)	2.648(10)
Rb(1)-Rb(2)	4.6633(18)
Rb(1)-Cl(1)	3.164(13)
Rb(2)-Cl(1)	3.7271(12)
Rb(2)-Cl(3)	3.352(3)

Rb(3)-Rb(4)	4.6012(15)
Rb(3)-Cl(7)	3.243(5)
Rb(4)-Cl(3)	3.285(4)
Rb(4)-Cl(5)	3.310(3)
Rb(4)-Cl(6)	3.247(6)
Rb(4)-Cl(7)	3.346(5)
Rb(5)-Cl(7)	3.507(5)
Rb(5)-Cl(8)	3.7340(13)
Rb(6)-Cl(6)	3.732(2)
Rb(6)-Cl(7)	3.403(6)
Rb(7)-Cl(6)	3.355(7)
Rb(7)-Cl(10)	3.45(2)
Rb(7)-Cl(11)	3.63(2)
Rb(8)-Rb(9)	0.998(8)
Rb(8)-Cl(8)	3.034(14)
Rb(8)-Cl(10)	3.32(2)
Rb(8)-Cl(13)	3.313(12)
Rb(9)-Cl(9)	2.973(10)
Rb(9)-Cl(10)	2.533(19)
Rb(9)-Cl(11)	3.336(7)
Rb(9)-Cl(12)	2.883(11)
Rb(9)-Cl(13)	2.826(11)
Rb(9)-Cl(14)	3.293(13)
Rb(10)-Cl(9)	3.282(7)
Rb(10)-Cl(11)	2.458(18)
Rb(10)-Cl(12)	3.354(11)
Rb(10)-Cl(13)	3.303(11)
Rb(10)-Cl(14)	2.492(19)
Rb(10)-Cl(16)	3.329(11)
Rb(11)-Cl(16)	3.323(11)
Rb(11)-Cl(18)	2.46(3)
Rb(11)-Cl(19)	2.35(4)
Rb(12)-Rb(13)	1.125(10)
Rb(12)-Cl(16)	2.897(11)
Rb(12)-Cl(17)	2.23(2)
Rb(12)-Cl(18)	3.24(2)
Rb(13)-Cl(17)	2.22(2)
Cl(10)-Cl(11)	1.767(13)
Cl(10)-Cl(12)	2.31(2)
Cl(11)-Cl(12)	2.270(18)
Cl(12)-Cl(13)	2.090(15)
Cl(13)-Cl(14)	2.20(2)
Cl(14)-Cl(15)	1.69(2)
Cl(14)-Cl(16)	2.17(2)
Cl(15)-Cl(16)	2.43(2)
Cl(17)-Cl(18)	1.89(3)

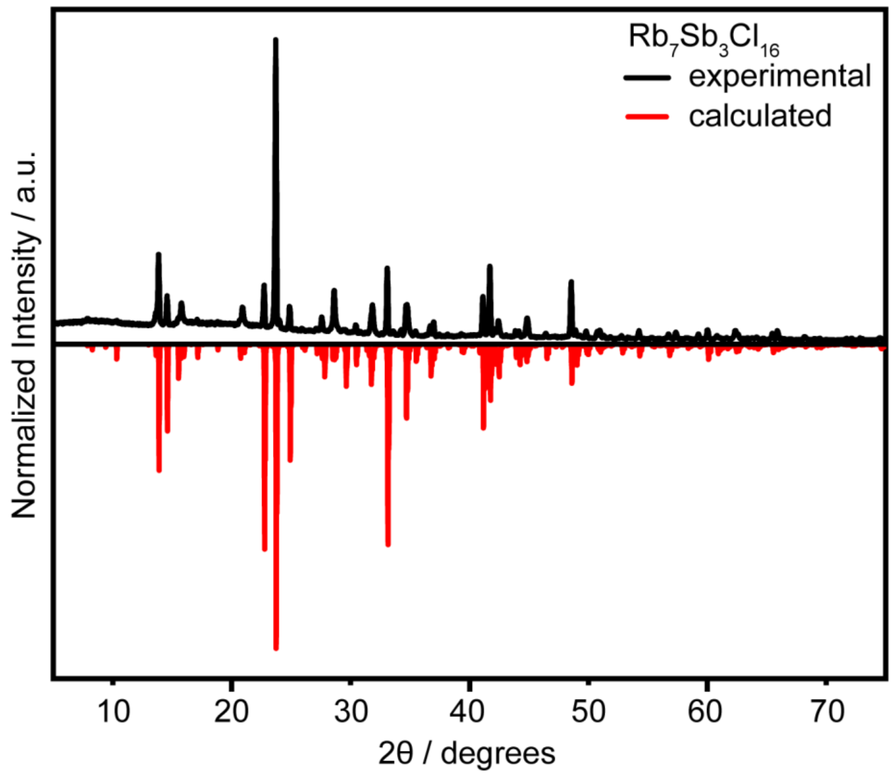

Figure S1. Experimental and calculated powder patterns of $Rb_7Sb_3Cl_{16}$. The $Rb_7Sb_3Cl_{16}$ diffraction pattern was calculated in Vesta[3] using the experimentally determined structure of $Rb_7Sb_3Cl_{16}$.

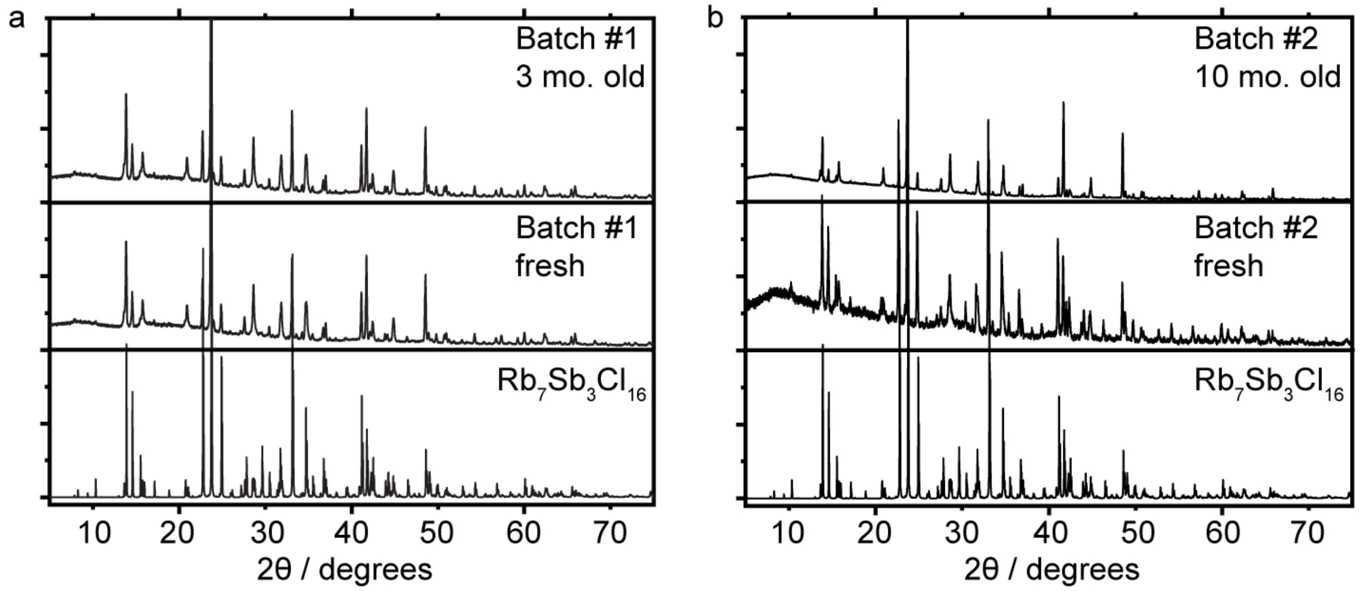

Figure S2. Structural stability of $Rb_7Sb_3Cl_{16}$. Two separate batches (a) and (b) were measured each after several months and no new phases were observed, supporting the oxidative stability of this material.

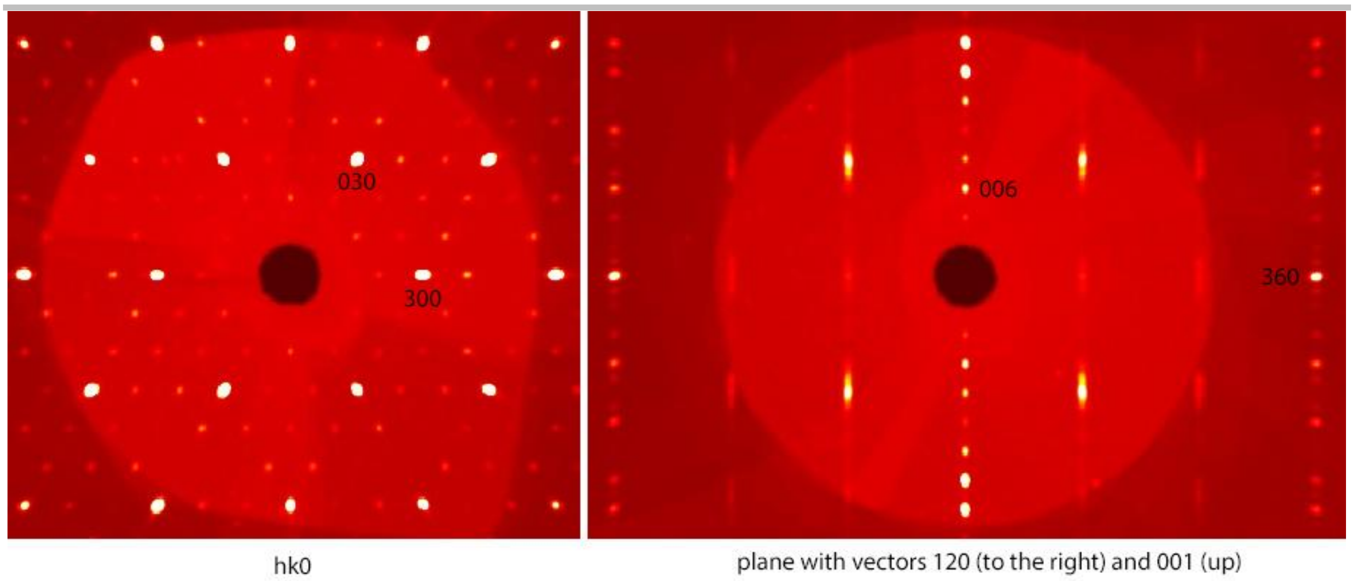

Figure S3. Unwrapped images from the 230 K diffraction experiment for $Rb_7Sb_3Cl_{16}$. The left panel demonstrates the six-fold symmetry present in the structure looking at the *ab* plane. The right image looks at the *c* axis and clearly demonstrates the combination of ordered and disordered units present in the structure. The discrete and well-resolved lines represent the ordered layers while the smeared lines in between are the disordered layers.

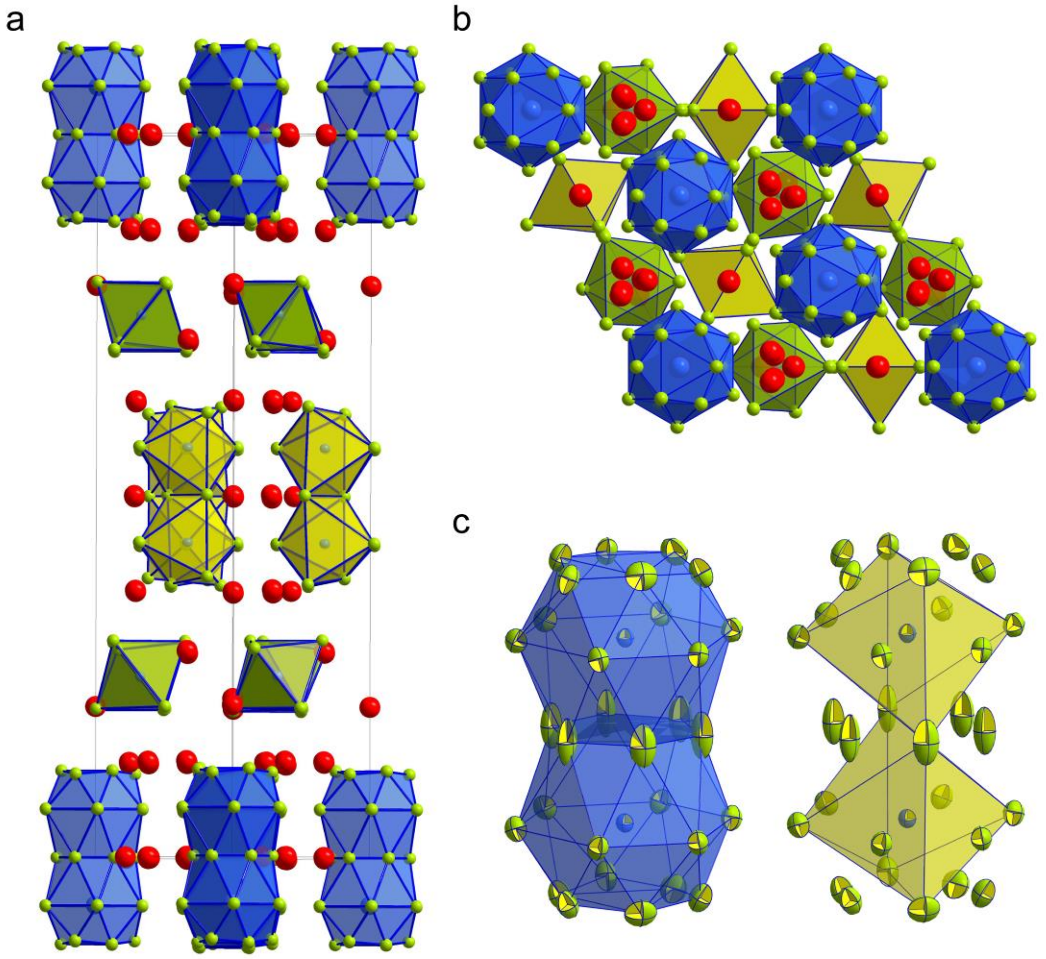

Figure S4. Disorder in the $Rb_7Sb_3Cl_{16}$ structure. a) The $Rb_7Sb_3Cl_{16}$ structure with color-coded ordered (yellow) and disordered (blue) units. Rubidium atoms are colored red. b) A view of the same structure down the *c*-axis depicting the three possible positions for the Rb (1/3 occupancy). c) A side-by-side comparison of the ordered and disordered $[Sb_2Cl_{10}]^{4-}$ dimers. The ordered dimers orient along one of three possible orientations as demonstrated by the yellow dimer (ellipsoids indicate 50% probability).

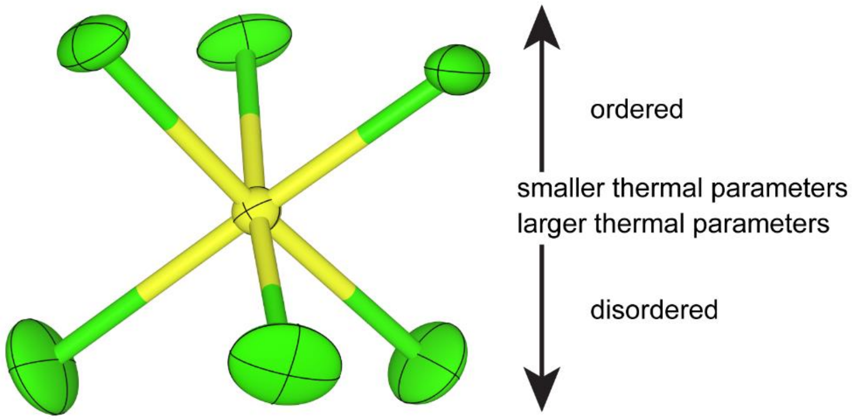

Figure S5. Thermal parameters of Cl atoms in the $[SbCl_6]^{3-}$ octahedra in the layer next to the disordered layer. Ellipsoids indicate 50% probability.

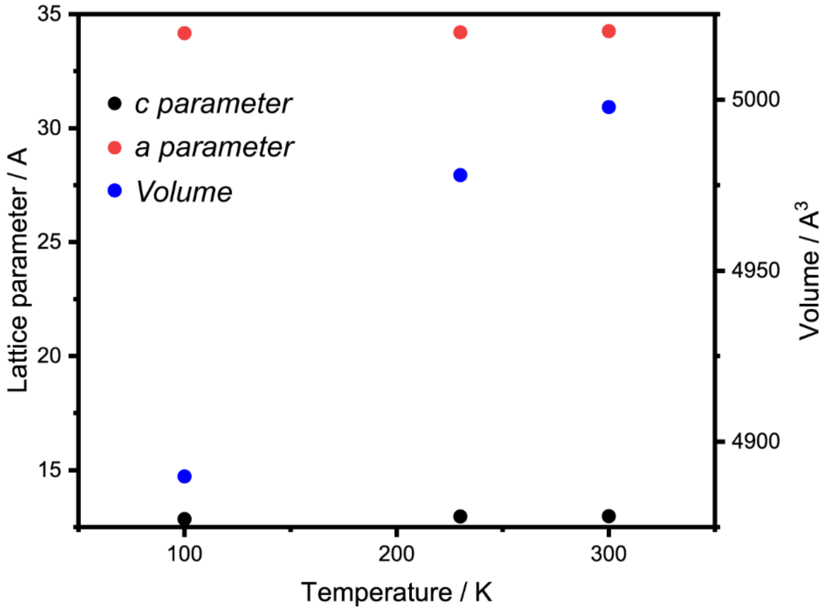

Figure S6. Lattice parameters and unit cell volume with temperature.

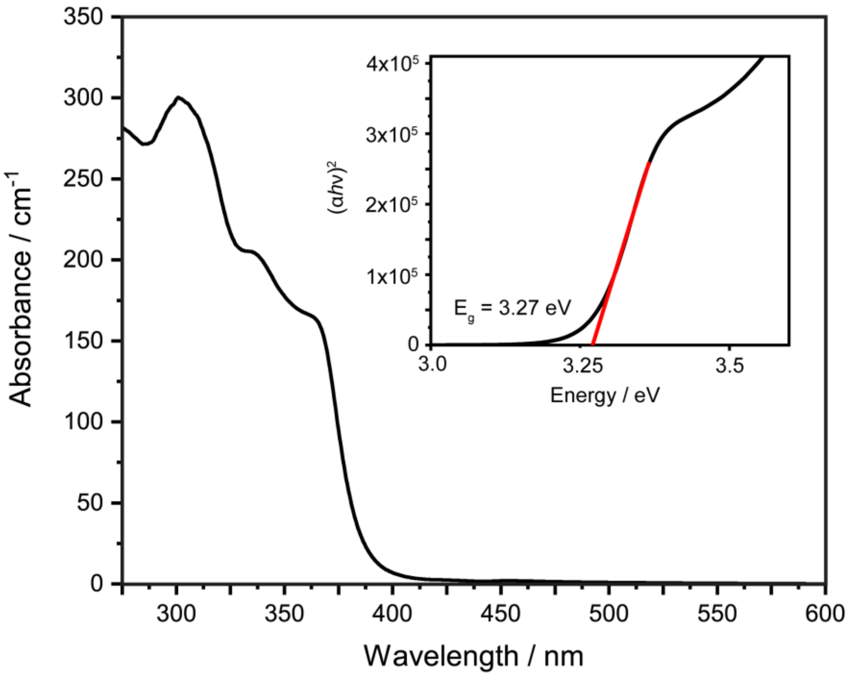

Figure S7. Absorption spectrum for $Rb_7Sb_3Cl_{16}$. Inset: Tauc plot for direct-gap semiconductor.

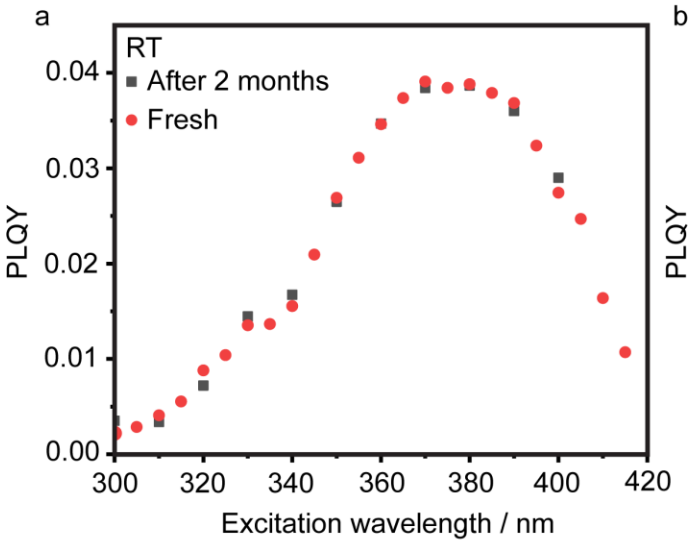

Figure S8. PLQY comparison at a) RT and b) 77 K.

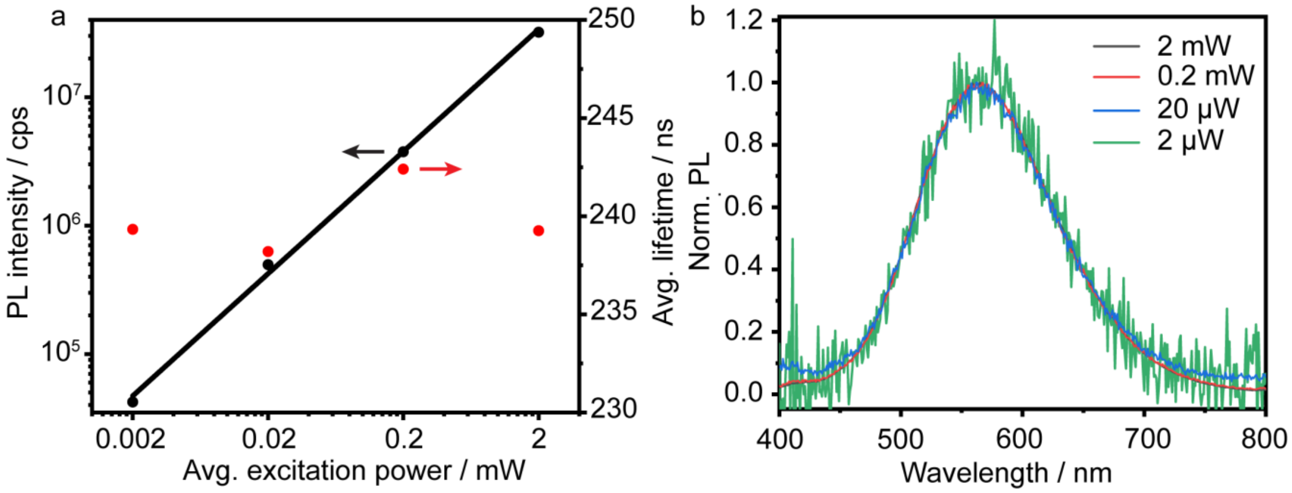

Figure S9. dW-PL of $Rb_7Sb_3Cl_{16}$: a) Log intensity vs. Log power, b) dW-PL spectra. The fitted line in (a) has a slope of m = 0.95 and R^2 = 0.998. The deviation in lifetime is the result of thermal fluctuations at room temperature.

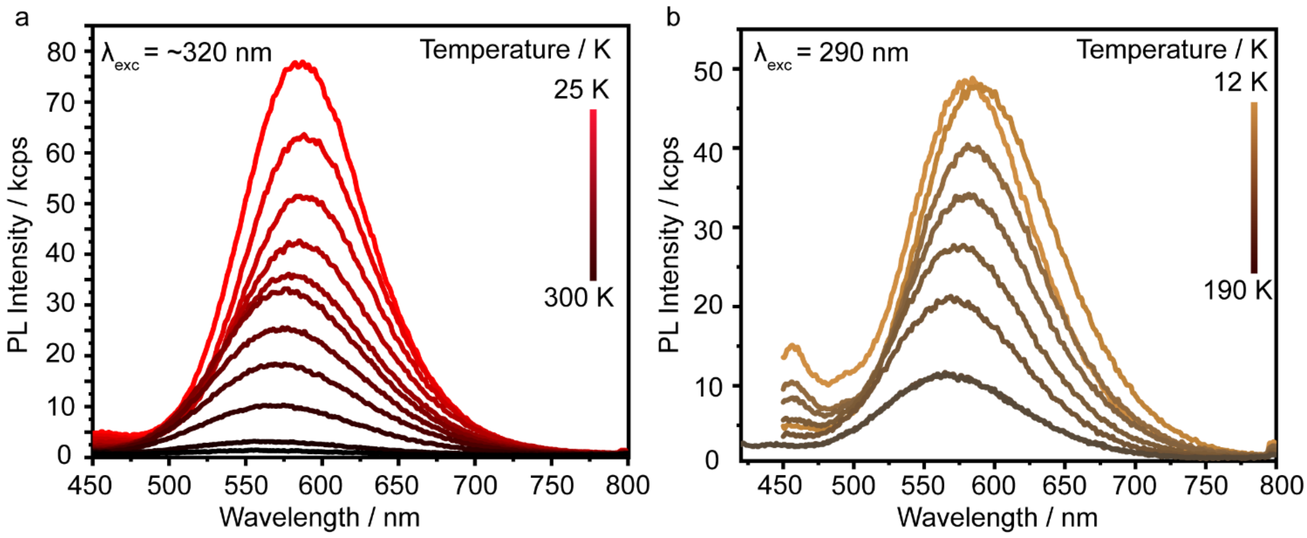

Figure S10. dT-PL spectra of $Rb_7Sb_3Cl_{16}$ with excitation at a) 320 nm and b) 290 nm.

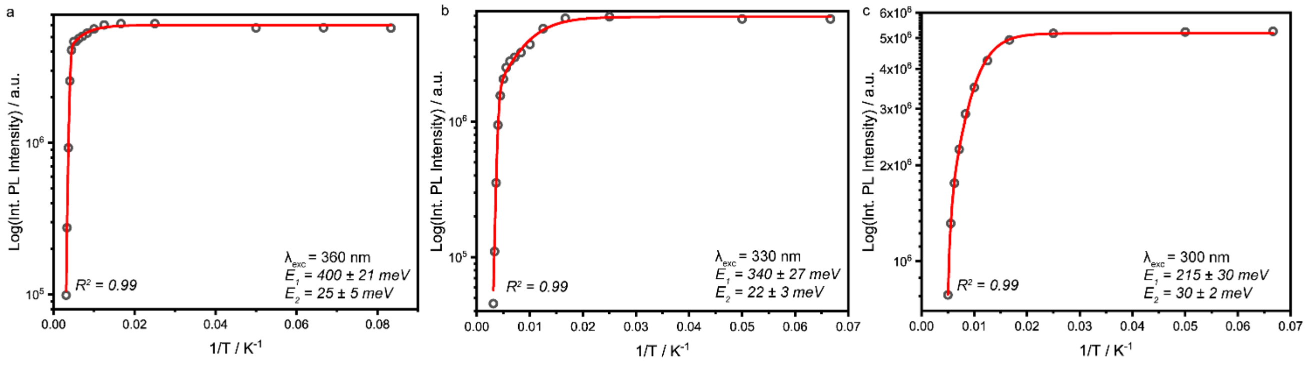

Figure S11. Arrhenius fits for the integrated PL-intensity of $Rb_7Sb_3Cl_{16}$ with two activation energies at a) 360 nm excitation, b) 330 nm excitation, and c) 300 nm excitation. The following formula was used during the fitting: $I_t = \frac{I_0}{1+C_1 e^{\frac{-E_1 T}{k_B 1000}} + C_2 e^{\frac{-E_2 T}{k_B 1000}}}$

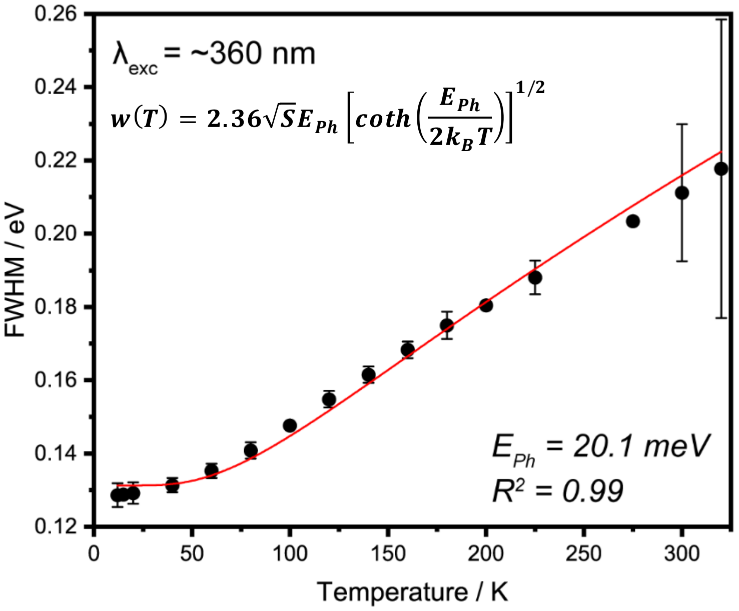

Figure S12. Temperature dependent broadening data from $Rb_7Sb_3Cl_{16}$ (excitation at PLE max) fitted with the Toyozawa model.

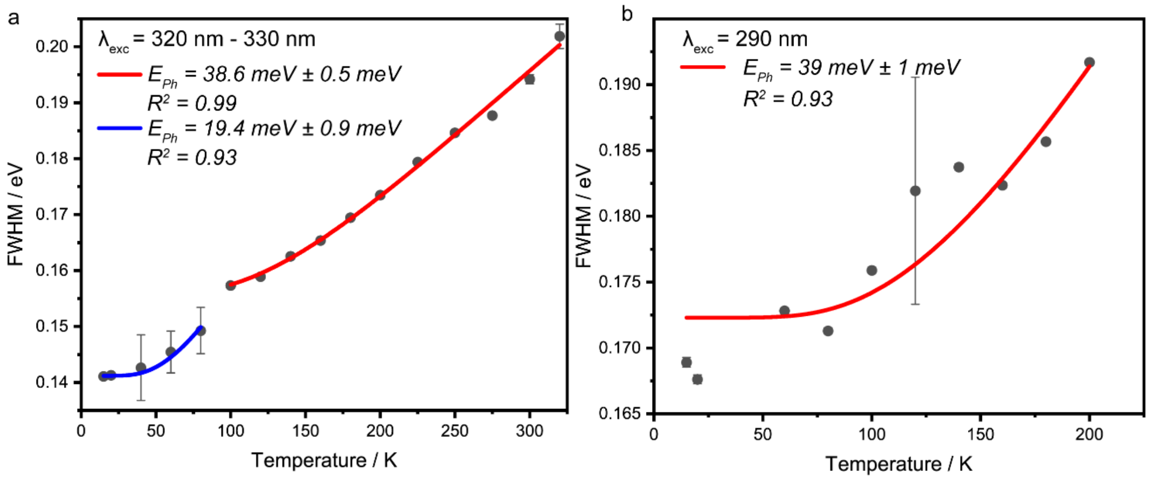

Figure S13. Temperature dependent broadening data from $Rb_7Sb_3Cl_{16}$ fitted with the Toyozawa model with excitation at a) 320 nm to 330 nm and at b) 290 nm. The fitting of the 290 nm excitation data indicates higher energy vibrational modes than those associated with the vibrational modes that couple to the structural feature excited at ~360 nm. These two different vibrational modes can both be found from fitting the dT-PL broadening data with ~330 nm excitation. The data around 100 K strongly deviates from the linear behaviour expected by the Toyozawa model and can be satisfactorily modelled only by the use of two separate fits. These demonstrate that, at low temperatures where features are narrower, primarily the lower-energy vibrational modes contribute (*i.e.* one structural feature); at higher temperatures, potentially as a result of spectral broadening, the high-energy vibrational modes dominate indicating the influence of the other structural feature.

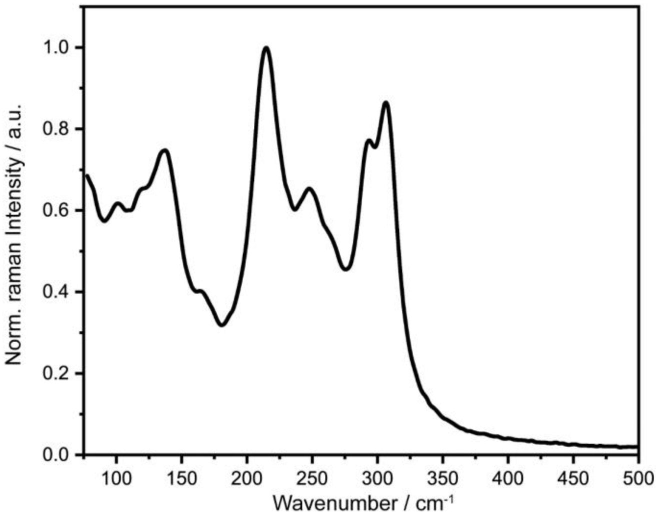

Figure S14. Raman spectra of $Rb_7Sb_3Cl_{16}$.

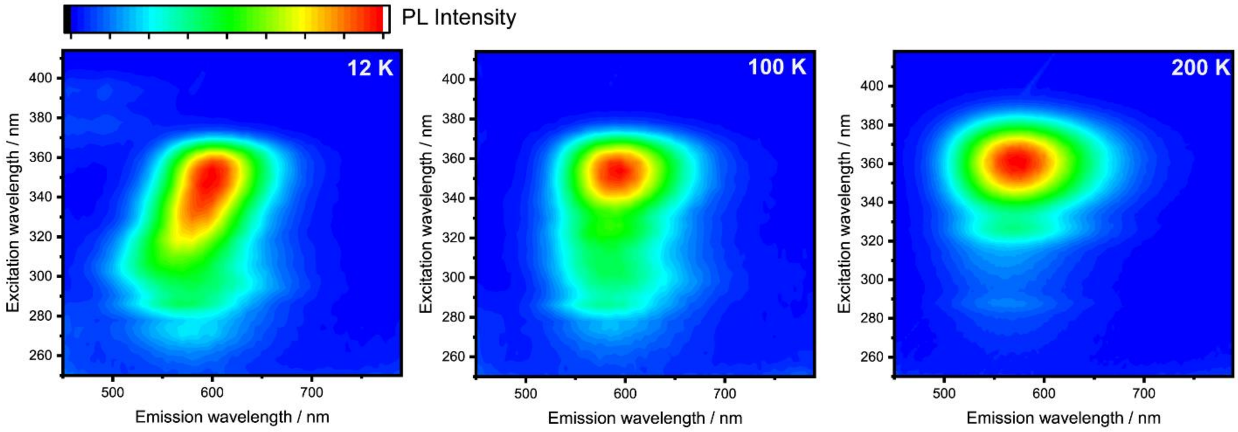

Figure S15. PL-PLE maps for $Rb_7Sb_3Cl_{16}$ measured at 12 K, 100 K, and 200 K.

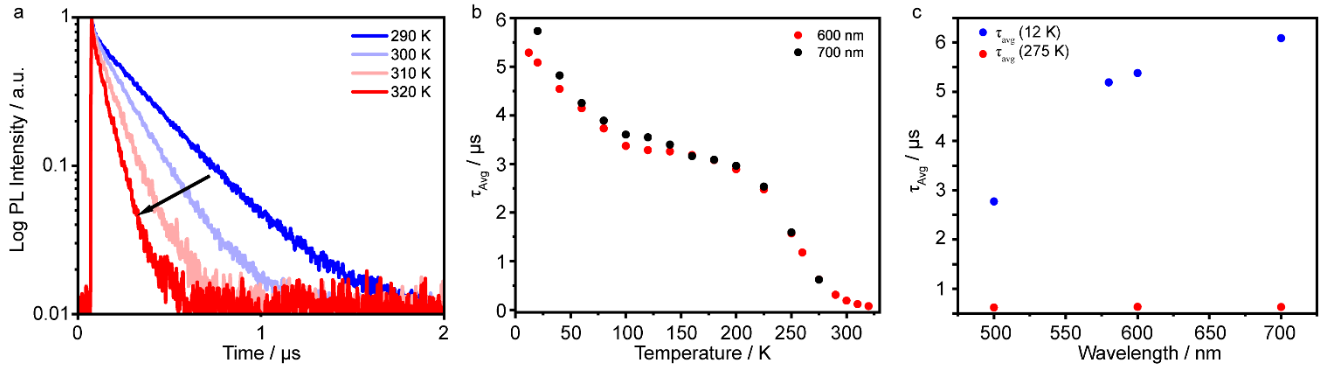

Figure S16. TRPL of $Rb_7Sb_3Cl_{16}$ a) TRPL of $Rb_7Sb_3Cl_{16}$ measured at various temperatures close to room temperature. b) Average, intensity-weighted, lifetime values for $Rb_7Sb_3Cl_{16}$ measured at two different emission wavelengths. c) Average, intensity-weighted, lifetime values for $Rb_7Sb_3Cl_{16}$ measured at different wavelengths and at different temperatures.

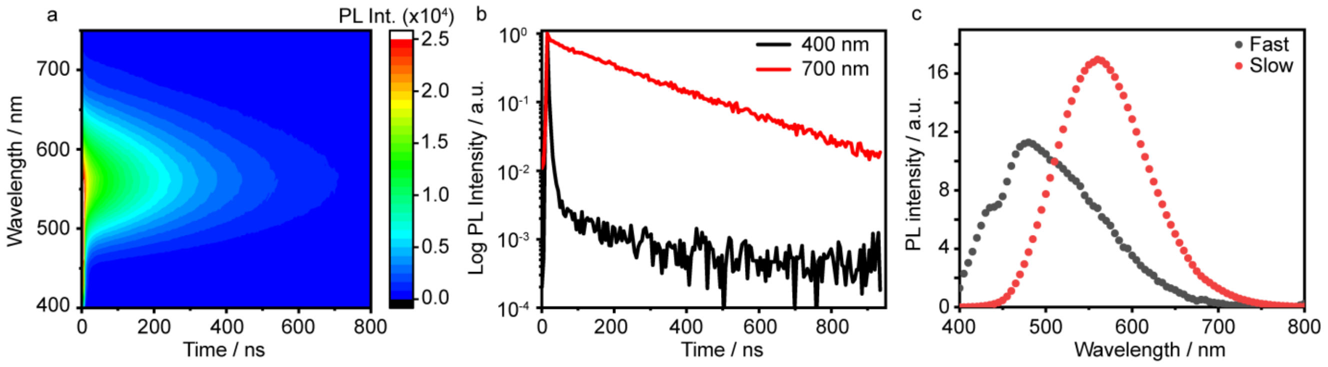

Figure S17. TRES for $Rb_7Sb_3Cl_{16}$: a) 2D TRES plot, c) lifetime slices at 400 nm and 700 nm, b) spectral slices of the fast (<50 ns) and slow components from the TRES experiment.

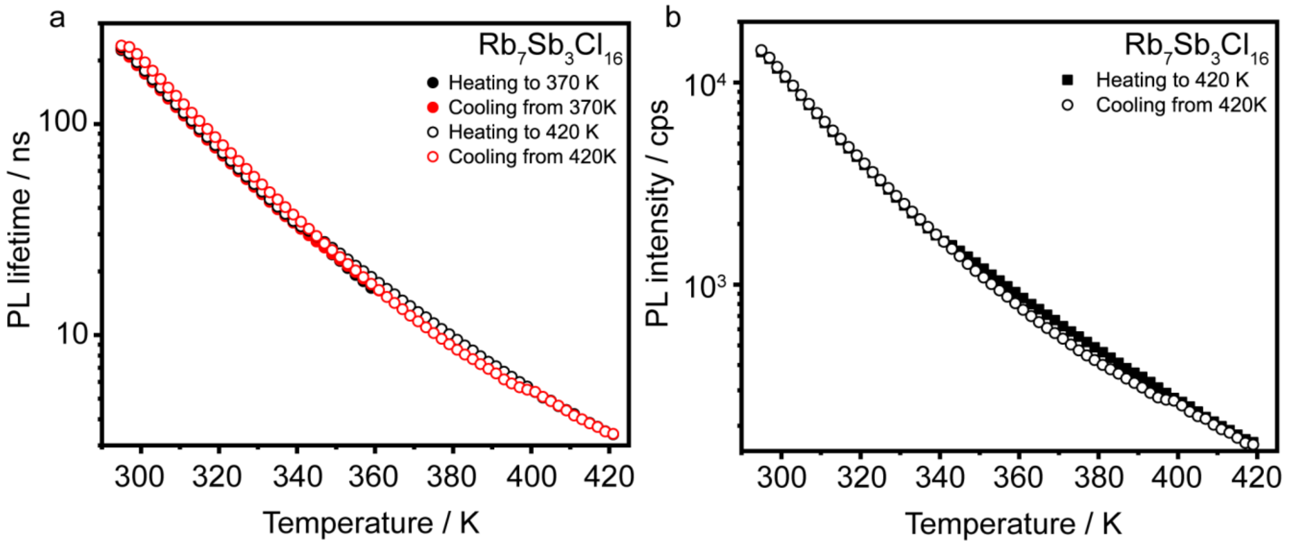

Figure S18. Thermal stability of $Rb_7Sb_3Cl_{16}$: a) PL lifetime as a function of temperature, b) PL intensity as a function of temperature. Lifetime and intensity were measured as separate runs.

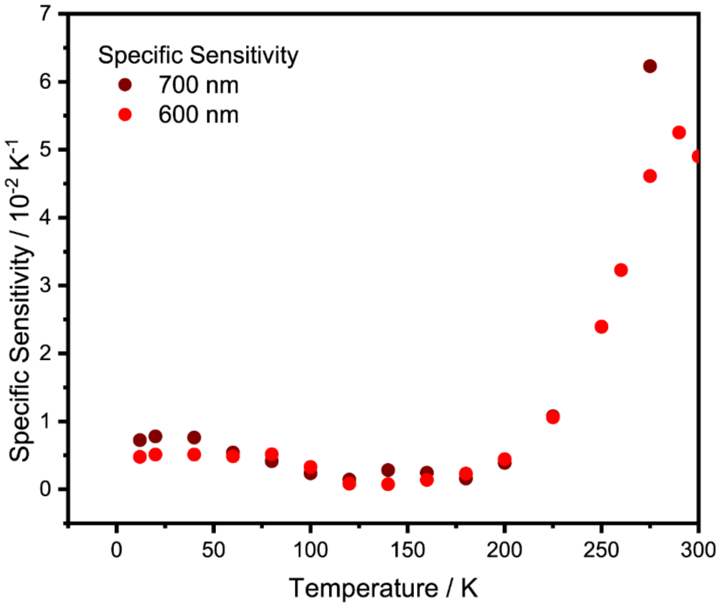

Figure S19. Specific temperature sensitivities (α) calculated for $Rb_7Sb_3Cl_{16}$ at two different wavelengths with respect to temperature according to the formula, $\alpha = -\frac{dt}{dT}\frac{1}{t}$.

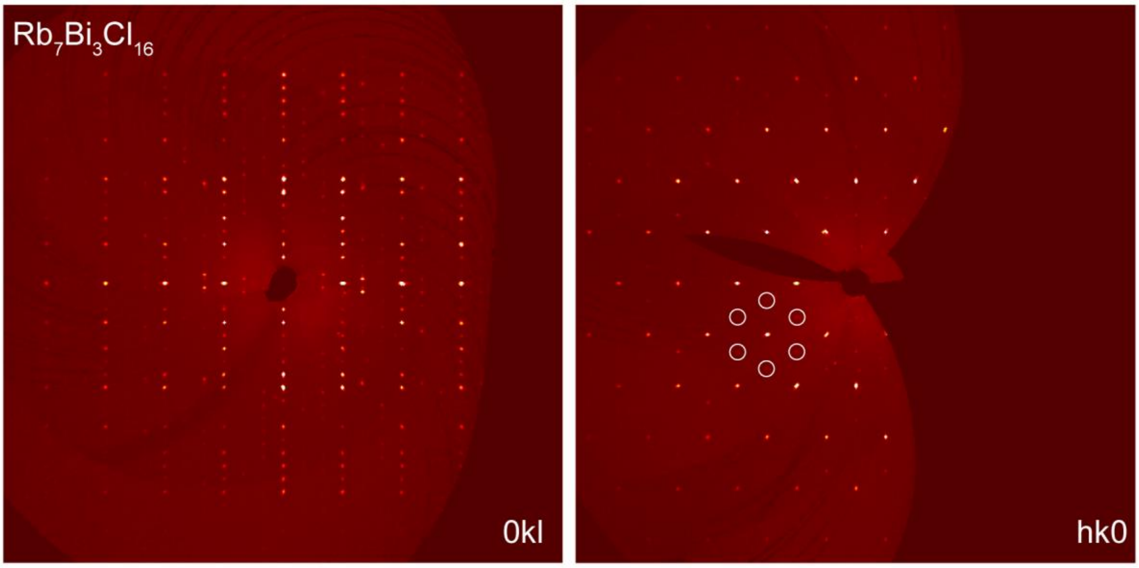

Figure S20. 0kl and hk0 unwrapped images for $Rb_7Bi_3Cl_{16}$.

Table S11. Crystal data and structure refinement for $Rb_7Bi_3Cl_{16}$ at 298 K.

Empirical formula	$Rb_7Bi_3Cl_{16}$
Formula weight	1792.43
Temperature	298 K
Wavelength	0.71073 Å
Crystal system	Hexagonal
Space group	*R*-3*c*
Unit cell dimensions	a = 13.10250(10) Å, α = 90°<br>b = 13.10250(10) Å, β = 90°<br>c = 102.9084(17) Å, γ = 120°
Volume	15299.9(3) $Å^3$
Z	18
Density (calculated)	3.502 g/cm^3
Absorption coefficient	26.700 mm^{-1}
F(000)	14040
Crystal size	0.071 x 0.058 x 0.04 mm^3
θ range for data collection	1.806 to 29.574°
Index ranges	-18<=h<=18, -13<=k<=18, -142<=l<=142
Reflections collected	60200
Independent reflections	4792 [R_{int} = 0.0878]
Completeness to θ = 25.242°	99.9%
Refinement method	Full-matrix least-squares on F^2
Data / restraints / parameters	4792 / 0 / 121
Goodness-of-fit	1.051
Final R indices [I > 2σ(I)]	R_{obs} = 0.0387, wR_{obs} = 0.0696
R indices [all data]	R_{all} = 0.0811, wR_{all} = 0.0778
Largest diff. peak and hole	1.180 and -1.552 e·$Å^{-3}$
Absolute structure	Refined with twin
Absolute structure parameter	0.206(3)

$R = \Sigma||F_o|-|F_c|| / \Sigma|F_o|$, $wR = \{\Sigma[w(|F_o|^2 - |F_c|^2)^2] / \Sigma[w(|F_o|^4)]\}^{1/2}$ and $w=1/[\sigma^2(Fo^2)+(0.0079P)^2+146.9450P]$ where $P=(Fo^2+2Fc^2)/3$

Table S12. Atomic coordinates ($x10^4$) and equivalent isotropic displacement parameters ($Å^2x10^3$) for $Rb_7Bi_3Cl_{16}$ at 298 K with estimated standard deviations in parentheses.

Label	x	y	z	Occupancy	U_{eq}*
Bi(1)	6666.67	3333.33	4980(1)	1	23(1)
Bi(2)	3331(1)	3351(1)	4385(1)	1	26(1)
Bi(3)	0	0	5000	1	22(1)
Rb(1)	9546(1)	6209(1)	5385(1)	1	35(1)
Rb(2)	9975(1)	6697(1)	4882(1)	1	61(1)
Rb(3)	6666.67	3333.33	4590(1)	1	40(1)
Rb(4)	6666.67	3333.33	4165(1)	1	36(1)
Rb(5)	3333.33	-534(1)	4166.67	1	34(1)
Rb(6)	0	0	4596(1)	1	40(1)
Cl(1)	8101(2)	5160(2)	5121(1)	1	56(1)
Cl(2)	8572(2)	3769(2)	4827(1)	1	47(1)
Cl(3)	5722(2)	4610(2)	4382(1)	1	34(1)
Cl(4)	3338(2)	4827(2)	4546(1)	1	58(1)
Cl(5)	3323(3)	2078(2)	4576(1)	1	58(1)
Cl(6)	3333.33	4749(2)	4166.67	1	35(1)
Cl(7)	3333.33	1924(2)	4166.67	1	50(1)
Cl(8)	932(2)	2211(2)	4385(1)	1	35(1)
Cl(9)	80(3)	1702(2)	4846(1)	1	49(1)

*U_{eq} is defined as one third of the trace of the orthogonalized U_{ij} tensor.

Table S13. Anisotropic displacement parameters (Å^{2}x10^3) for $Rb_7Bi_3Cl_{16}$ at 298 K with estimated standard deviations in parentheses.

Label	U_{11}	U_{22}	U_{33}	U_{12}	U_{13}	U_{23}
Bi(1)	23(1)	23(1)	21(1)	12(1)	0	0
Bi(2)	25(1)	28(1)	23(1)	13(1)	1(1)	-1(1)
Bi(3)	22(1)	22(1)	22(1)	11(1)	0	0
Rb(1)	36(1)	43(1)	29(1)	22(1)	-1(1)	-2(1)
Rb(2)	66(1)	43(1)	58(1)	16(1)	3(1)	-1(1)
Rb(3)	47(1)	47(1)	25(1)	24(1)	0	0
Rb(4)	42(1)	42(1)	26(1)	21(1)	0	0
Rb(5)	28(1)	31(1)	40(1)	14(1)	-1(1)	-1(1)
Rb(6)	47(1)	47(1)	26(1)	23(1)	0	0
Cl(1)	56(2)	48(2)	40(2)	8(2)	-13(2)	-16(2)
Cl(2)	36(2)	67(2)	40(2)	28(2)	6(1)	-7(2)
Cl(3)	30(2)	39(2)	33(1)	16(1)	1(1)	2(1)
Cl(4)	38(2)	53(2)	81(2)	21(2)	-1(2)	-35(2)
Cl(5)	77(2)	55(2)	43(2)	36(2)	-1(2)	15(2)
Cl(6)	29(2)	32(2)	43(2)	14(1)	2(2)	1(1)
Cl(7)	86(3)	39(2)	39(2)	43(2)	-1(2)	0(1)
Cl(8)	32(2)	35(2)	34(1)	14(1)	0(1)	0(1)
Cl(9)	82(2)	39(2)	37(2)	39(2)	-5(2)	5(1)

The anisotropic displacement factor exponent takes the form: $-2\pi^2[h^2a^{*2}U_{11} + ... + 2hka^*b^*U_{12}]$.

Table S14. Selected bond lengths [Å] for $Rb_7Bi_3Cl_{16}$ at 298 K with estimated standard deviations in parentheses.

Label	Distances
Bi(1)-Rb(2)	4.4871(10)
Bi(1)-Rb(3)	4.0144(13)
Bi(1)-Cl(1)	2.621(2)
Bi(1)-Cl(2)	2.762(2)
Bi(2)-Cl(3)	2.714(2)
Bi(2)-Cl(4)	2.544(2)
Bi(2)-Cl(5)	2.578(2)
Bi(2)-Cl(6)	2.8957(17)
Bi(2)-Cl(7)	2.9218(18)
Bi(2)-Cl(8)	2.723(2)
Bi(3)-Rb(6)	4.1545(12)
Bi(3)-Cl(9)	2.694(2)
Rb(1)-Cl(1)	3.195(2)
Rb(2)-Cl(1)	3.348(3)
Rb(2)-Cl(2)	3.371(3)
Rb(3)-Rb(4)	4.3724(16)
Rb(3)-Cl(2)	3.325(2)
Rb(3)-Cl(3)	3.315(2)
Rb(3)-Cl(5)	3.837(3)
Rb(4)-Cl(3)	3.373(2)
Rb(4)-Cl(7)	3.7974(3)
Rb(5)-Cl(7)	3.220(3)
Rb(6)-Cl(5)	3.815(3)
Rb(6)-Cl(8)	3.328(2)
Rb(6)-Cl(9)	3.370(2)

Supplementary Note 1. Choosing the ordered model over the disordered model for $Rb_7Bi_3Cl_{16}$.

As shown in Fig. S14, the unwrapped images of the diffraction pattern exhibit weak superstructure that remains unaccounted for with the $P3_1c$ space group, which results in a disordered structure. Furthermore, the *R*-3*c* solution accounts for these weak points and has lower correlation matrix values than the disordered solution.

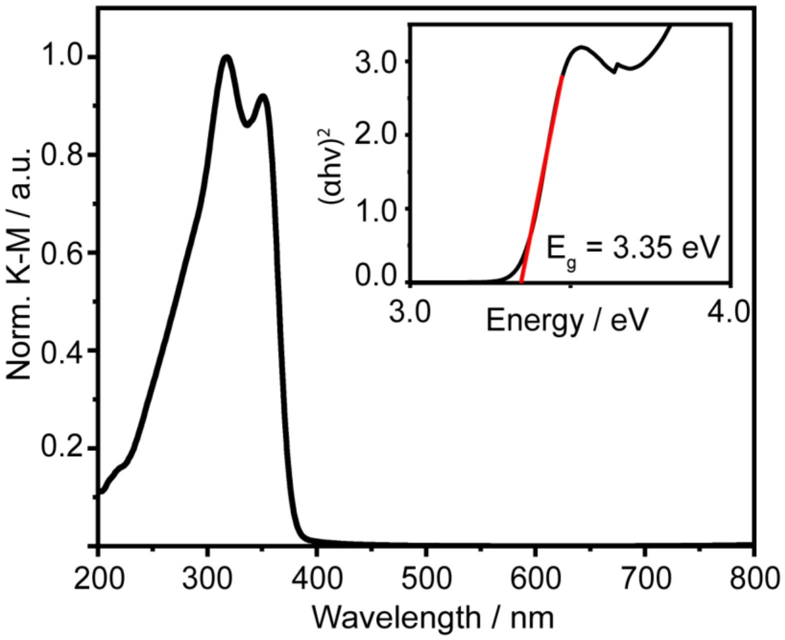

Figure S21. Absorption spectra of $Rb_7Bi_3Cl_{16}$ with Tauc plot shown in the inset. The absorption spectrum was measured in diffuse reflectance and recalculated using the Kubelka-Munk transformation. The UV absorption agrees with the colorless appearance of the crystals. No PL was observed at room temperature regardless of excitation wavelength.

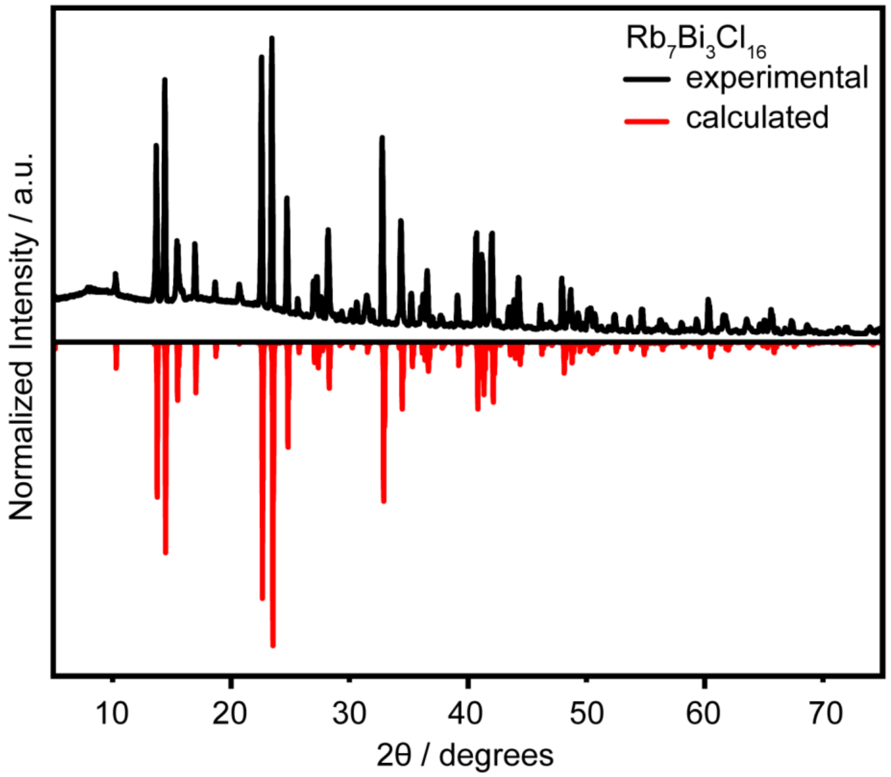

Figure S22. Experimental and calculated powder patterns of $Rb_7Bi_3Cl_{16}$. The diffraction pattern was calculated in Vesta[3] using the experimentally determined structure of $Rb_7Bi_3Cl_{16}$.

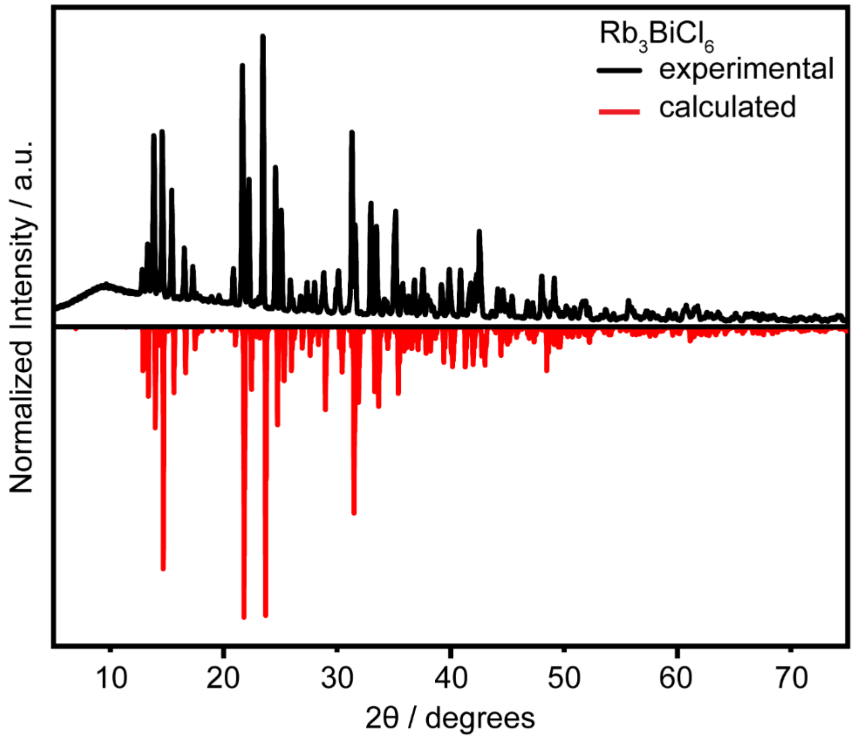

Figure S23. Experimental and calculated powder patterns of Rb_3BiCl_6. The Rb_3BiCl_6 diffraction pattern was calculated in Vesta[3] using the experimentally determined structure of Rb_3BiCl_6.

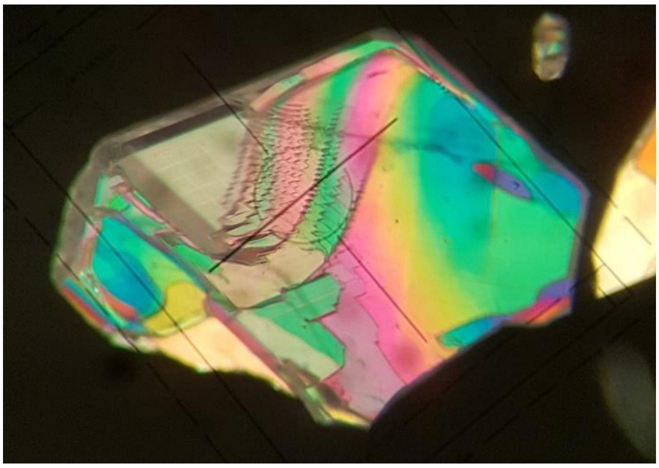

Figure S24. Photos of Rb_3BiCl_6 crystals under polarized light. Their interaction with polarized light also them to be more easily separated from crystals of $Rb_7Bi_3Cl_{16}$ system, which grow as thin platelets (like the $Rb_7Sb_3Cl_{16}$ system) and do not rotate polarized light indicating that their high symmetry axis must be oriented perpendicular to the large, flat face of the platelet.

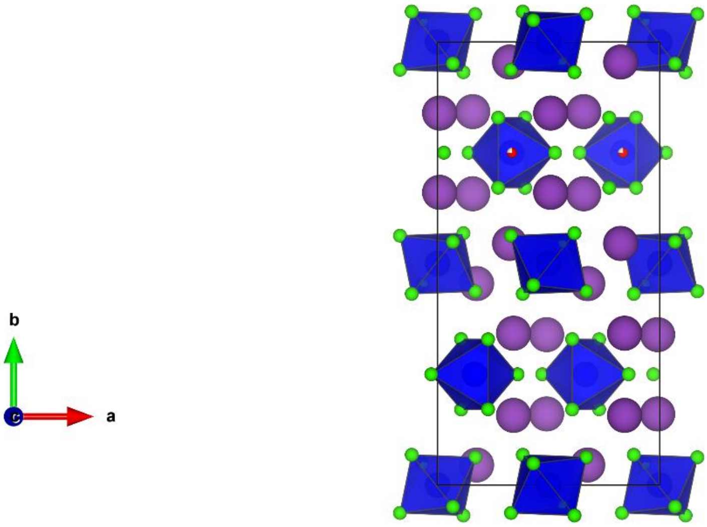

Figure S25. Structure of Rb_3BiCl_6 at 298 K.

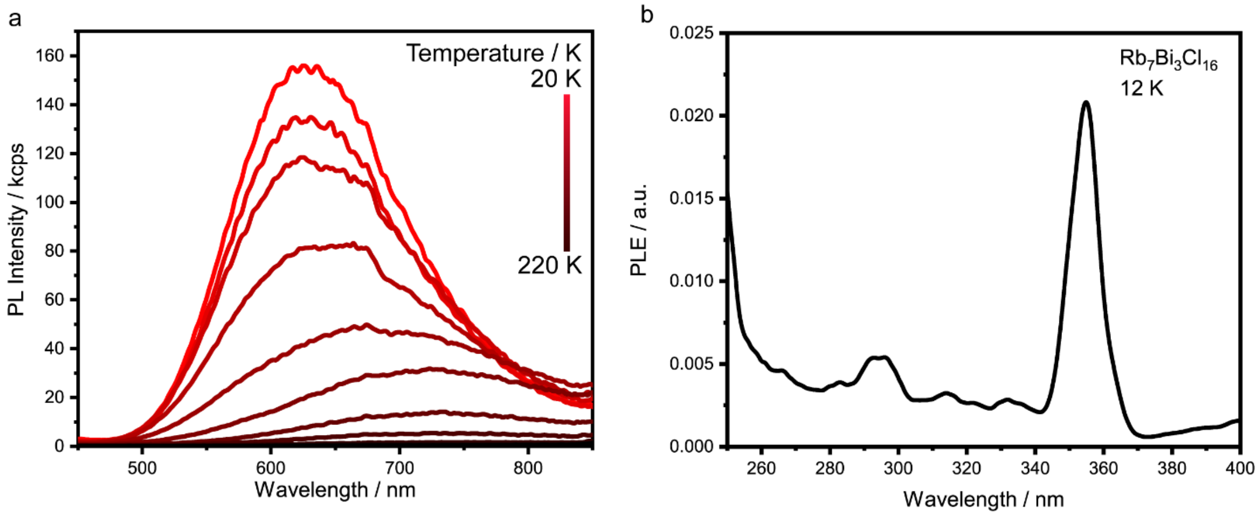

Figure S26. a) dT-PL for $Rb_7Bi_3Cl_{16}$ and b) PLE at 12 K.

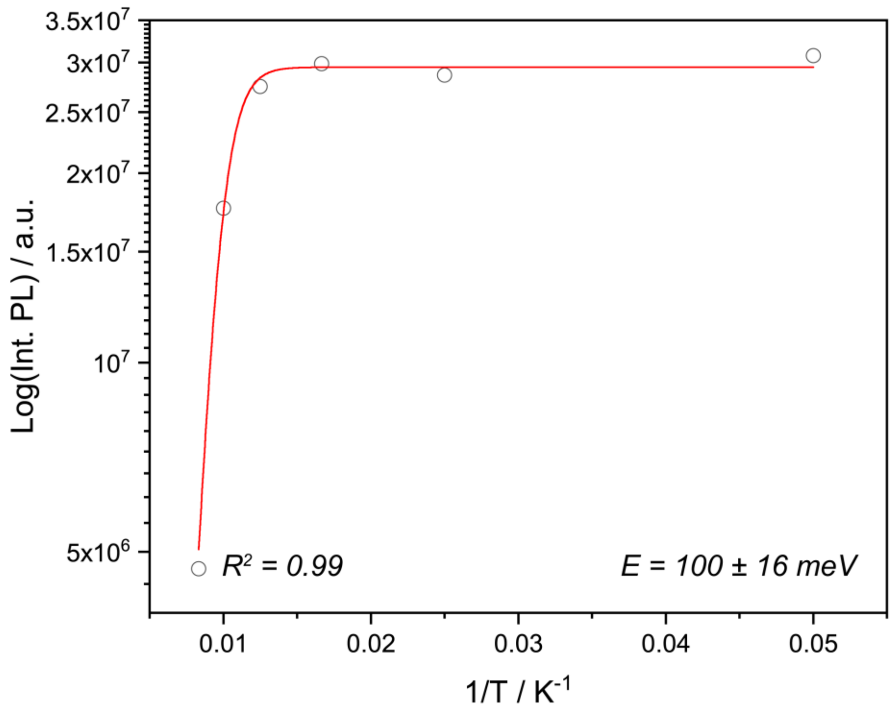

Figure S27. Arrhenius plot of PL intensity vs. T for $Rb_7Bi_3Cl_{16}$. The activation energy of *ca.* 100 meV is much lower than that determined for the pure-Sb and agrees with the experimental observation that this material is quenched at far lower temperatures then $Rb_7Sb_3Cl_{16}$.

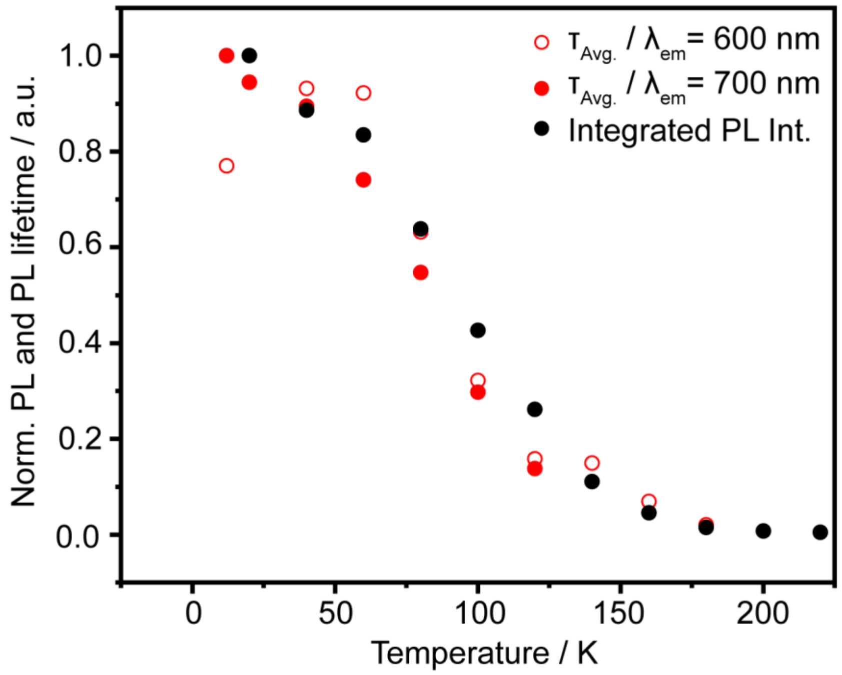

Figure S28. Normalized PL lifetime and PL intensity vs. temperature for $Rb_7Bi_3Cl_{16}$.

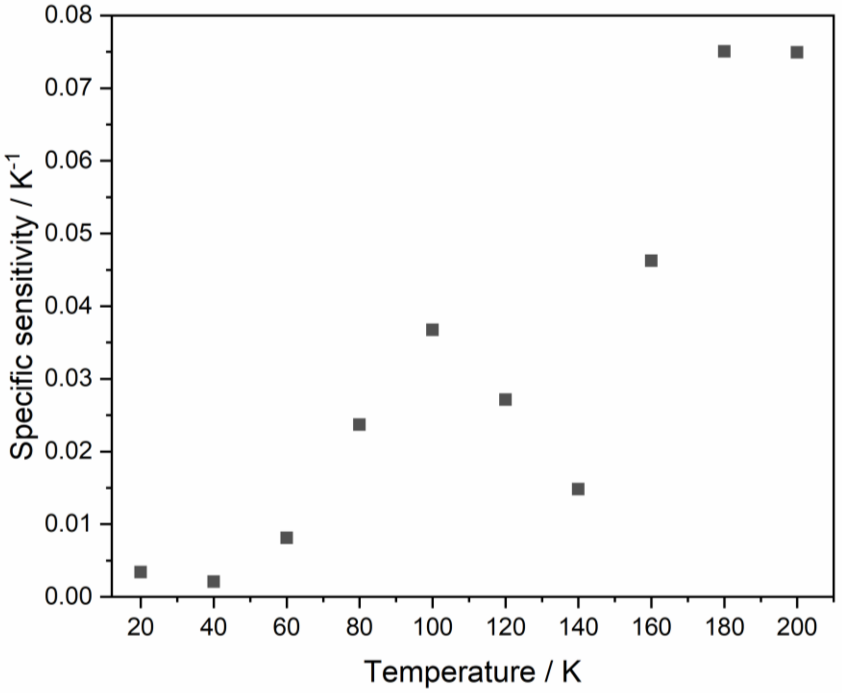

Figure S29. Specific sensitivity of $Rb_7Bi_3Cl_{16}$.

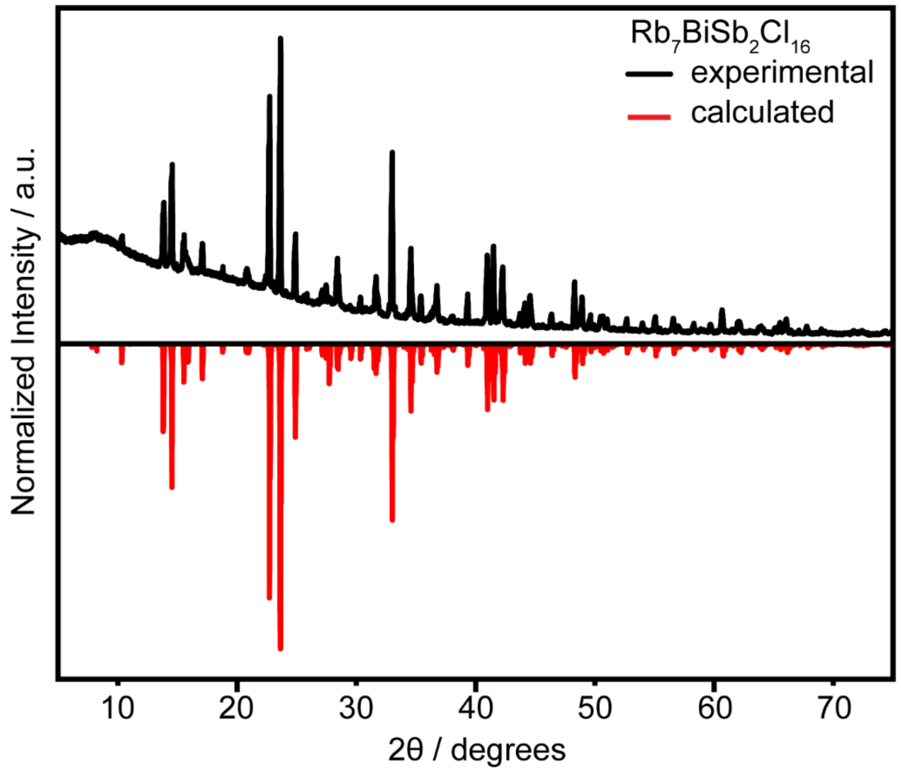

Figure S30. Experimental and calculated powder patterns of $Rb_7BiSb_2Cl_{16}$. The $Rb_7BiSb_2Cl_{16}$ diffraction pattern was calculated in Vesta[3] using the experimentally determined structure of $Rb_7BiSb_2Cl_{16}$.

Table S15. Crystal data and structure refinement for $Rb_7Sb_{2.07}Bi_{0.93}Cl_{16}$ at 296(3) K.

Empirical formula	$Rb_7Sb_{2.07}Bi_{0.93}Cl_{16}$
Formula weight	1612.15
Temperature	296(3) K
Wavelength	0.71073 Å
Crystal system	Hexagonal
Space group	*P*-62*m*
Unit cell dimensions	a = 13.0332(2) Å, α = 90°<br>b = 13.0332(2) Å, β = 90°<br>c = 34.1956(5) Å, γ = 120°
Volume	5030.40(17) $Å^3$
Z	6
Density (calculated)	3.193 g/cm^3
Absorption coefficient	17.904 mm^{-1}
F(000)	4283
Crystal size	0.375 x 0.3 x 0.188 mm^3
θ range for data collection	1.804 to 35.484°
Index ranges	-21<=h<=21, -21<=k<=20, -54<=l<=54
Reflections collected	149507
Independent reflections	8103 [R_{int} = 0.0844]
Completeness to θ = 25.242°	99.8%
Refinement method	Full-matrix least-squares on F^2
Data / restraints / parameters	8103 / 0 / 186
Goodness-of-fit	1.054
Final R indices [I > 2σ(I)]	R_{obs} = 0.0539, wR_{obs} = 0.1651
R indices [all data]	R_{all} = 0.0712, wR_{all} = 0.1798
Largest diff. peak and hole	4.582 and -1.702 e·$Å^{-3}$

$R = \Sigma||F_o|-|F_c|| / \Sigma|F_o|$, $wR = \{\Sigma[w(|F_o|^2 - |F_c|^2)^2] / \Sigma[w(|F_o|^4)]\}^{1/2}$ and $w=1/[\sigma^2(Fo^2)+(0.0813P)^2+39.1137P]$ where $P=(Fo^2+2Fc^2)/3$

Table S16. Atomic coordinates ($x10^4$) and equivalent isotropic displacement parameters ($Å^2x10^3$) for $Rb_7Sb_{2.07}Bi_{0.93}Cl_{16}$ at 296(3) K with estimated standard deviations in parentheses.

Label	x	y	z	Occupancy	U_{eq}*
Rb(1)	6622(2)	10000	2149(1)	1	58(1)
Rb(2)	6683(2)	6683(2)	2843(1)	1	57(1)
Rb(3)	7114(2)	7114(2)	1345(1)	1	34(1)
Rb(4)	6666.67	3333.33	1290(1)	1	42(1)
Rb(5)	6666.67	3333.33	0	1	38(1)
Rb(6)	7202(2)	7202(2)	0	1	33(1)
Rb(7)	3332(2)	3875(3)	5000	0.5	37(1)
Rb(8)	3333.33	6666.67	5000	0.5	36(2)
Rb(9)	10000	10000	5000	1	38(1)
Rb(10)	3333.33	6666.67	3707(2)	0.5	34(1)
Rb(11)	10000	10000	3709(1)	1	41(1)
Rb(12)	6222(2)	9553(2)	3658(1)	0.5	35(1)
Cl(1)	4838(5)	8408(4)	2914(2)	1	71(2)
Cl(2)	5148(5)	6927(5)	2017(1)	1	59(2)
Cl(3)	8071(6)	9602(6)	2999(2)	0.5	48(2)
Cl(4)	8427(6)	8427(6)	2128(2)	1	130(5)
Cl(5)	1893(5)	1893(5)	1143(2)	1	66(2)
Cl(6)	4554(5)	4554(5)	1228(2)	1	63(2)
Cl(7)	4437(3)	2073(3)	663(1)	1	36(1)
Cl(8)	4748(7)	4748(7)	0	1	69(3)
Cl(9)	1912(6)	1912(6)	0	1	57(2)
Cl(10)	5232(10)	6657(7)	5000	0.5	63(4)
Cl(11)	6680(15)	8109(12)	5000	0.5	86(6)

Cl(12)	4303(5)	5407(6)	4338(2)	0.5	36(2)
Cl(13)	7764(5)	9023(5)	4341(2)	0.5	36(2)
Cl(14)	5216(9)	6669(7)	3864(3)	0.5	66(3)
Cl(15)	6666(8)	7899(8)	3797(3)	0.5	64(2)
Bi(01)	3333.33	6666.67	2480(1)	0.408(10)	24(1)
Bi(02)	10000	10000	2551(1)	0.327(13)	24(1)
Bi(03)	3312(1)	3312(1)	657(1)	0.171(9)	24(1)
Bi(04)	6656(1)	6656(1)	4343(1)	0.381(10)	28(1)
Sb(1)	3333.33	6666.67	2480(1)	0.592(10)	24(1)
Sb(2)	10000	10000	2551(1)	0.673(13)	24(1)
Sb(3)	3312(1)	3312(1)	657(1)	0.829(9)	24(1)
Sb(4)	6656(1)	6656(1)	4343(1)	0.619(10)	28(1)

*U_{eq} is defined as one third of the trace of the orthogonalized U_{ij} tensor.

Table S17. Anisotropic displacement parameters (Å^{2}x10^3) for $Rb_7Sb_{2.07}Bi_{0.93}Cl_{16}$ at 296(3) K with estimated standard deviations in parentheses.

Label	U_{11}	U_{22}	U_{33}	U_{12}	U_{13}	U_{23}
Rb(1)	66(2)	40(2)	58(2)	20(1)	2(1)	0
Rb(2)	56(2)	56(2)	46(2)	18(1)	0(1)	0(1)
Rb(3)	44(1)	44(1)	21(1)	26(1)	-2(1)	-2(1)
Rb(4)	53(1)	53(1)	19(1)	26(1)	0	0
Rb(5)	46(2)	46(2)	21(1)	23(1)	0	0
Rb(6)	35(1)	35(1)	31(1)	19(1)	0	0
Rb(7)	27(2)	28(2)	56(2)	13(2)	0	0
Rb(8)	38(2)	38(2)	32(2)	19(1)	0	0
Rb(9)	41(2)	41(2)	33(2)	20(1)	0	0
Rb(10)	39(2)	39(2)	26(2)	19(1)	0	0
Rb(11)	43(2)	43(2)	35(2)	22(1)	0	0
Rb(12)	34(2)	38(2)	36(1)	20(2)	1(1)	-1(1)
Cl(1)	69(3)	43(2)	63(2)	-3(2)	15(2)	-15(2)
Cl(2)	62(2)	105(3)	37(2)	62(3)	-10(2)	-21(2)
Cl(3)	31(3)	66(4)	47(3)	25(3)	7(2)	-6(3)
Cl(4)	113(4)	113(4)	34(3)	-42(5)	-15(2)	-15(2)
Cl(5)	59(3)	59(3)	97(5)	43(3)	41(3)	41(3)
Cl(6)	68(3)	68(3)	40(2)	23(3)	-15(2)	-15(2)
Cl(7)	38(2)	42(2)	29(1)	21(2)	0(1)	0(2)
Cl(8)	49(4)	49(4)	85(6)	6(4)	0	0
Cl(9)	42(3)	42(3)	91(6)	24(4)	0	0
Cl(10)	39(5)	26(5)	122(12)	15(4)	0	0
Cl(11)	112(14)	41(6)	125(13)	53(8)	0	0
Cl(12)	27(2)	37(3)	42(2)	16(2)	0(2)	0(2)
Cl(13)	33(3)	28(3)	38(2)	9(2)	-2(2)	1(2)
Cl(14)	62(5)	55(5)	82(6)	31(4)	-39(5)	-2(4)
Cl(15)	69(6)	53(4)	76(6)	35(4)	2(4)	22(4)
Bi(01)	22(1)	22(1)	29(1)	11(1)	0	0
Bi(02)	25(1)	25(1)	22(1)	12(1)	0	0
Bi(03)	27(1)	27(1)	18(1)	14(1)	1(1)	1(1)
Bi(04)	28(1)	28(1)	28(1)	13(1)	-1(1)	-1(1)
Sb(1)	22(1)	22(1)	29(1)	11(1)	0	0
Sb(2)	25(1)	25(1)	22(1)	12(1)	0	0
Sb(3)	27(1)	27(1)	18(1)	14(1)	1(1)	1(1)
Sb(4)	28(1)	28(1)	28(1)	13(1)	-1(1)	-1(1)

The anisotropic displacement factor exponent takes the form: $-2\pi^2[h^2a^{*2}U_{11} + ... + 2hka^*b^*U_{12}]$.

Table S18. Bond lengths [Å] for $Rb_7Sb_{2.07}Bi_{0.93}Cl_{16}$ at 296(3) K with estimated standard deviations in parentheses.

Label	Distances
Rb(1)-Cl(1)	3.426(5)
Rb(1)-Cl(2)	3.498(5)
Rb(1)-Cl(3)	3.642(8)
Rb(1)-Cl(4)	3.816(2)

Rb(1)-Bi(01)	4.4619(15)
Rb(2)-Cl(2)	3.564(4)
Rb(2)-Cl(3)	3.339(7)
Rb(2)-Cl(4)	3.337(8)
Rb(2)-Cl(15)	3.632(11)
Rb(2)-Bi(01)	4.5280(13)
Rb(2)-Bi(02)	4.437(2)
Rb(3)-Rb(4)	4.6670(11)
Rb(3)-Rb(6)	4.6004(14)
Rb(3)-Cl(2)	3.359(4)
Rb(3)-Cl(4)	3.179(6)
Rb(3)-Cl(6)	3.360(7)
Rb(4)-Rb(5)	4.4130(18)
Rb(4)-Cl(6)	3.8130(10)
Rb(4)-Cl(7)	3.313(3)
Rb(5)-Cl(7)	3.392(3)
Rb(5)-Cl(8)	3.7767(8)
Rb(6)-Cl(8)	3.198(9)
Rb(7)-Rb(8)	3.637(4)
Rb(7)-Cl(10)	3.210(10)
Rb(7)-Cl(12)	2.860(6)
Rb(8)-Cl(10)	2.480(12)
Rb(8)-Cl(12)	3.389(6)
Rb(9)-Cl(13)	3.388(6)
Rb(10)-Rb(12)	3.767(3)
Rb(10)-Cl(1)	3.450(5)
Rb(10)-Cl(12)	3.320(6)
Rb(10)-Cl(14)	2.510(9)
Rb(11)-Cl(3)	3.344(7)
Rb(11)-Cl(13)	3.328(6)
Rb(12)-Cl(1)	3.042(5)
Rb(12)-Cl(3)	3.275(6)
Rb(12)-Cl(13)	3.373(6)
Rb(12)-Cl(14)	3.379(8)
Rb(12)-Cl(15)	2.541(9)
Cl(1)-Bi(01)	2.597(5)
Cl(1)-Sb(1)	2.597(5)
Cl(2)-Bi(01)	2.723(4)
Cl(2)-Sb(1)	2.723(4)
Cl(3)-Bi(02)	2.764(6)
Cl(3)-Sb(2)	2.764(6)
Cl(4)-Bi(02)	2.508(6)
Cl(4)-Sb(2)	2.508(6)
Cl(5)-Bi(03)	2.486(5)
Cl(5)-Sb(3)	2.486(5)
Cl(6)-Bi(03)	2.535(5)
Cl(6)-Sb(3)	2.535(5)
Cl(7)-Bi(03)	2.670(3)
Cl(7)-Sb(3)	2.670(3)
Cl(8)-Bi(03)	2.925(6)
Cl(9)-Bi(03)	2.896(5)
Cl(9)-Sb(3)	2.896(5)
Cl(10)-Cl(11)	1.890(19)
Cl(10)-Bi(04)	2.916(8)
Cl(11)-Bi(04)	2.930(8)
Cl(12)-Cl(14)	2.191(11)
Cl(12)-Bi(04)	2.659(6)
Cl(12)-Sb(4)	2.659(6)
Cl(13)-Cl(15)	2.359(12)
Cl(13)-Bi(04)	2.673(6)
Cl(13)-Sb(4)	2.673(6)
Cl(14)-Cl(15)	1.779(13)
Cl(14)-Bi(04)	2.498(8)
Cl(14)-Sb(4)	2.498(8)
Cl(15)-Bi(04)	2.467(8)
Cl(15)-Sb(4)	2.467(8)

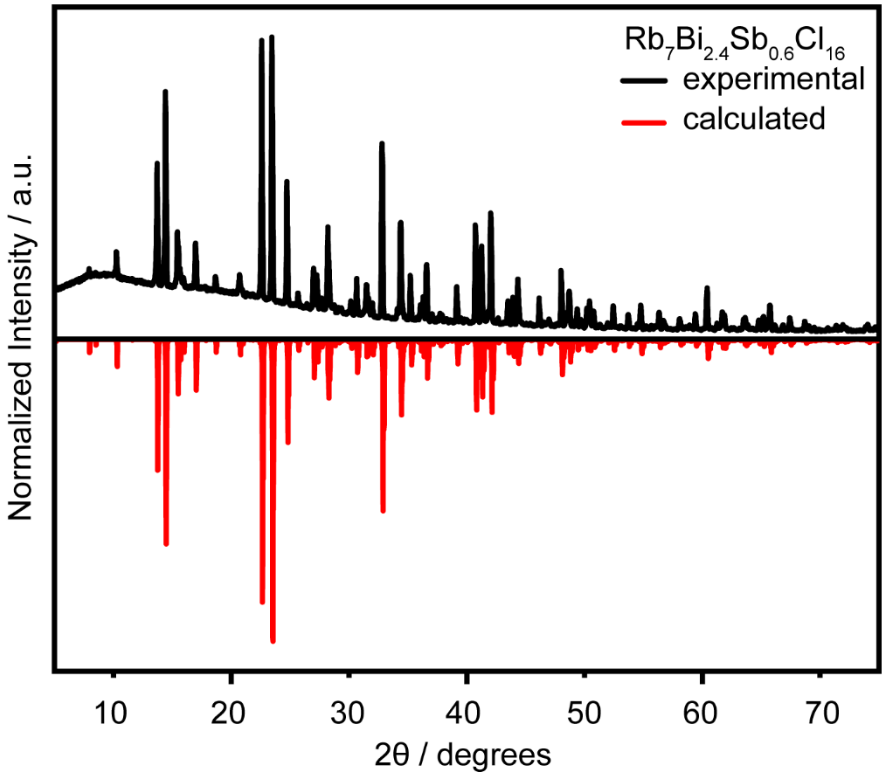

Figure S31. Experimental and calculated powder patterns of $Rb_7Bi_{2.4}Sb_{0.6}Cl_{16}$. The $Rb_7Bi_{2.4}Sb_{0.6}Cl_{16}$ diffraction pattern was calculated in Vesta[3] using the experimentally determined structure of $Rb_7Bi_{2.4}Sb_{0.6}Cl_{16}$.

Table S19. Crystal data and structure refinement for $Rb_7Bi_{2.41}Sb_{0.59}Cl_{16}$ at 300 K.

Empirical formula	$Rb_7Bi_{2.41}Sb_{0.59}Cl_{16}$
Formula weight	1740.72
Temperature	300 K
Wavelength	0.71073 Å
Crystal system	Hexagonal
Space group	*R*-3*c*
Unit cell dimensions	a = 13.08950(10) Å, α = 90°<br>b = 13.08950(10) Å, β = 90°<br>c = 102.8418(13) Å, γ = 120°
Volume	15259.7(3) $Å^3$
Z	18
Density (calculated)	3.410 g/cm^3
Absorption coefficient	24.171 mm^{-1}
F(000)	13699
Crystal size	0.203 x 0.124 x 0.076 mm^3
θ range for data collection	1.188 to 33.775°
Index ranges	-20<=h<=20, -20<=k<=20, -160<=l<=159
Reflections collected	223325
Independent reflections	6819 [R_{int} = 0.0764]
Completeness to θ = 25.242°	100%
Refinement method	Full-matrix least-squares on F^2
Data / restraints / parameters	6819 / 0 / 123
Goodness-of-fit	1.070
Final R indices [I > 2σ(I)]	R_{obs} = 0.0269, wR_{obs} = 0.0473
R indices [all data]	R_{all} = 0.0623, wR_{all} = 0.0556
Largest diff. peak and hole	0.807 and -1.667 e·$Å^{-3}$

$R = \Sigma||F_o|-|F_c|| / \Sigma|F_o|$, $wR = \{\Sigma[w(|F_o|^2 - |F_c|^2)^2] / \Sigma[w(|F_o|^4)]\}^{1/2}$ and $w=1/[\sigma^2(Fo^2)+(0.0183P)^2+53.8518P]$ where $P=(Fo^2+2Fc^2)/3$

Table S20. Atomic coordinates ($x10^4$) and equivalent isotropic displacement parameters ($Å^2x10^3$) for $Rb_7Bi_{2.41}Sb_{0.59}Cl_{16}$ at 300 K with estimated standard deviations in parentheses.

Label	x	y	z	Occupancy	U_{eq}*
Bi(1)	0	0	5000	0.917(4)	22(1)
Bi(2)	3333.33	6666.67	5019(1)	0.879(3)	24(1)
Bi(3)	3351(1)	3331(1)	5615(1)	0.758(2)	26(1)
Rb(3)	6211(1)	9550(1)	4615(1)	1	35(1)
Rb(5)	-538(1)	3333.33	5833.33	1	35(1)
Rb(6)	0	0	5832(1)	1	39(1)
Rb(4)	3333.33	6666.67	5409(1)	1	41(1)
Rb(1)	0	0	5404(1)	1	41(1)
Rb(2)	6723(1)	6695(1)	4883(1)	1	61(1)
Cl(4)	4602(1)	5709(1)	5617(1)	1	36(1)
Cl(9)	2220(1)	944(1)	5614(1)	1	36(1)
Cl(7)	4750(1)	3333.33	5833.33	1	40(1)
Cl(1)	1702(1)	88(2)	5154(1)	1	49(1)
Cl(3)	4810(1)	6232(1)	5172(1)	1	48(1)
Cl(8)	4816(1)	3336(1)	5455(1)	1	62(1)
Cl(6)	1912(1)	3333.33	5833.33	1	56(1)
Cl(2)	5150(1)	8096(1)	4878(1)	1	58(1)
Cl(5)	2088(1)	3323(2)	5424(1)	1	60(1)
Sb(3)	3351(1)	3331(1)	5615(1)	0.242(2)	26(1)
Sb(1)	0	0	5000	0.083(4)	22(1)
Sb(2)	3333.33	6666.67	5019(1)	0.121(3)	24(1)

*U_{eq} is defined as one third of the trace of the orthogonalized U_{ij} tensor.

Table S21. Anisotropic displacement parameters (Å^{2}x10^3) for $Rb_7Bi_{2.41}Sb_{0.59}Cl_{16}$ at 300 K with estimated standard deviations in parentheses.

Label	U_{11}	U_{22}	U_{33}	U_{12}	U_{13}	U_{23}
Bi(1)	23(1)	23(1)	21(1)	11(1)	0	0
Bi(2)	24(1)	24(1)	22(1)	12(1)	0	0
Bi(3)	28(1)	26(1)	23(1)	13(1)	1(1)	-1(1)
Rb(3)	42(1)	37(1)	31(1)	23(1)	3(1)	1(1)
Rb(5)	32(1)	29(1)	43(1)	14(1)	0(1)	1(1)
Rb(6)	44(1)	44(1)	29(1)	22(1)	0	0
Rb(4)	48(1)	48(1)	27(1)	24(1)	0	0
Rb(1)	48(1)	48(1)	26(1)	24(1)	0	0
Rb(2)	79(1)	44(1)	58(1)	28(1)	-4(1)	-1(1)
Cl(4)	40(1)	32(1)	36(1)	19(1)	-1(1)	-1(1)
Cl(9)	36(1)	32(1)	38(1)	14(1)	0(1)	-1(1)
Cl(7)	34(1)	31(1)	53(1)	15(1)	0(1)	0(1)
Cl(1)	39(1)	81(1)	38(1)	37(1)	-4(1)	4(1)
Cl(3)	47(1)	68(1)	42(1)	39(1)	-14(1)	-6(1)
Cl(8)	54(1)	38(1)	88(1)	19(1)	35(1)	-2(1)
Cl(6)	42(1)	89(2)	52(1)	44(1)	-1(1)	-1(1)
Cl(2)	49(1)	58(1)	42(1)	7(1)	17(1)	12(1)
Cl(5)	58(1)	77(1)	48(1)	37(1)	-15(1)	2(1)
Sb(3)	28(1)	26(1)	23(1)	13(1)	1(1)	-1(1)
Sb(1)	23(1)	23(1)	21(1)	11(1)	0	0
Sb(2)	24(1)	24(1)	22(1)	12(1)	0	0

The anisotropic displacement factor exponent takes the form: $-2\pi^2[h^2a^{*2}U_{11} + ... + 2hka^*b^*U_{12}]$.

Table S22. Selected bond lengths [Å] for $Rb_7Bi_{2.41}Sb_{0.59}Cl_{16}$ at 300 K with estimated standard deviations in parentheses.

Label	Distances
Bi(1)-Rb(1)	4.1492(6)
Bi(1)-Cl(1)	2.6881(9)
Bi(2)-Cl(3)	2.7626(9)
Bi(2)-Cl(2)	2.6085(10)
Bi(3)-Cl(4)	2.6960(9)
Bi(3)-Cl(9)	2.7080(9)
Bi(3)-Cl(7)	2.8944(8)
Bi(3)-Cl(8)	2.5302(10)
Bi(3)-Cl(6)	2.9294(9)
Bi(3)-Cl(5)	2.5641(10)
Rb(3)-Rb(2)	4.9483(6)
Rb(3)-Cl(2)	3.1981(10)
Rb(5)-Rb(6)	4.7547(3)
Rb(5)-Cl(6)	3.2071(14)
Rb(6)-Rb(1)	4.4079(7)
Rb(6)-Cl(9)	3.3780(9)
Rb(6)-Cl(6)	3.79226(15)
Rb(4)-Cl(4)	3.3138(9)
Rb(4)-Cl(3)	3.3287(11)
Rb(4)-Cl(5)	3.8346(13)
Rb(4)-Sb(2)	4.0077(6)
Rb(1)-Cl(9)	3.3265(9)
Rb(1)-Cl(1)	3.3626(11)
Rb(1)-Cl(5)	3.8139(13)
Rb(1)-Sb(1)	4.1492(6)
Rb(2)-Cl(3)	3.7393(12)
Rb(2)-Cl(2)	3.3739(14)
Cl(4)-Sb(3)	2.6960(9)
Cl(9)-Sb(3)	2.7080(9)
Cl(7)-Sb(3)	2.8944(8)
Cl(1)-Sb(1)	2.6881(9)
Cl(3)-Sb(2)	2.7626(9)
Cl(8)-Sb(3)	2.5302(10)

Cl(2)-Sb(2)	2.6085(9)
Cl(5)-Sb(3)	2.5641(10)

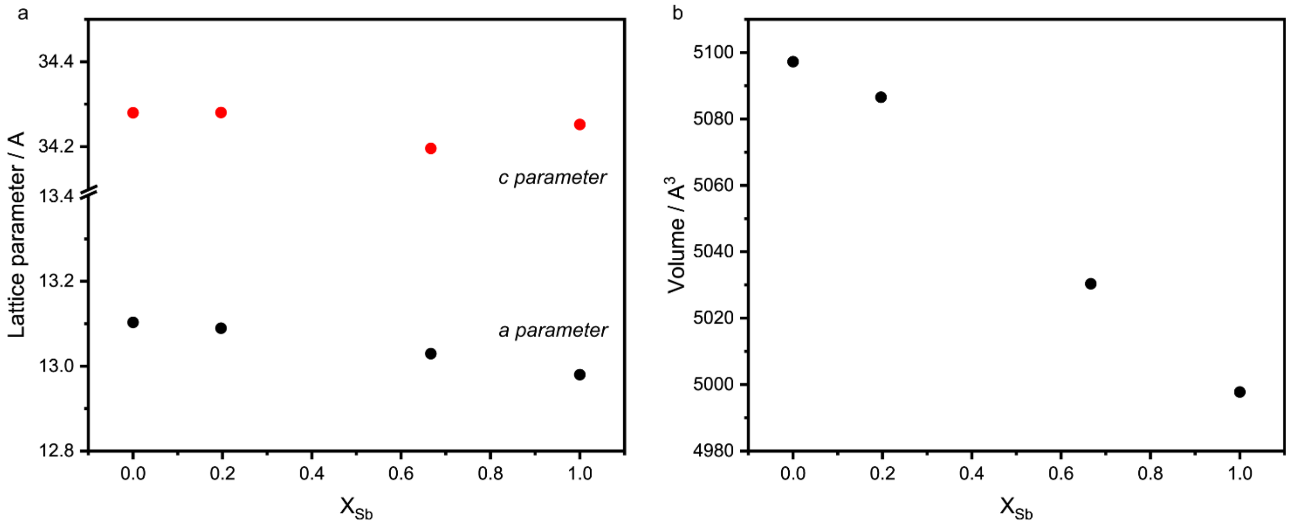

Figure S32. Vegard's law for the $Rb_7Bi_{3-3x}Sb_{3x}Cl_{16}$ family. a) lattice parameter a is strongly affected while c stays relatively constant. b) The volume follows a linear trend in agreement with Vegard's law.

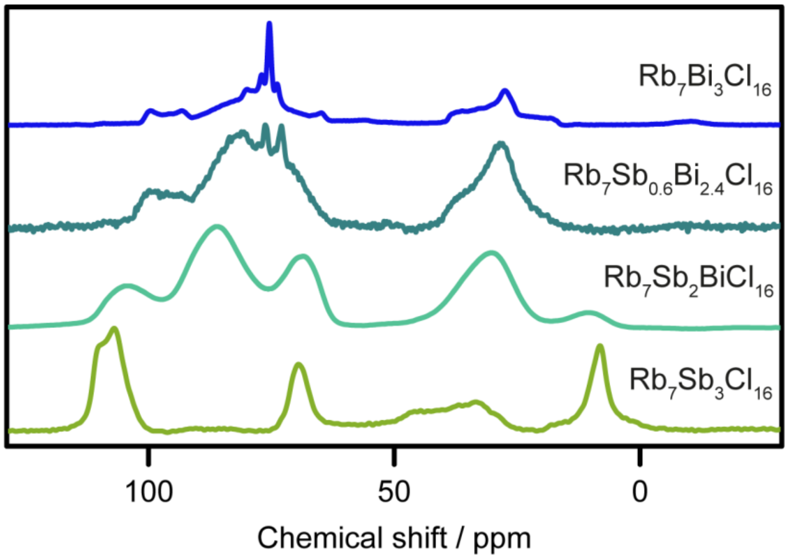

Figure S33. 1D ^{87}Rb NMR spectra for the $Rb_7Bi_{3-3x}Sb_{3x}Cl_{16}$ family.

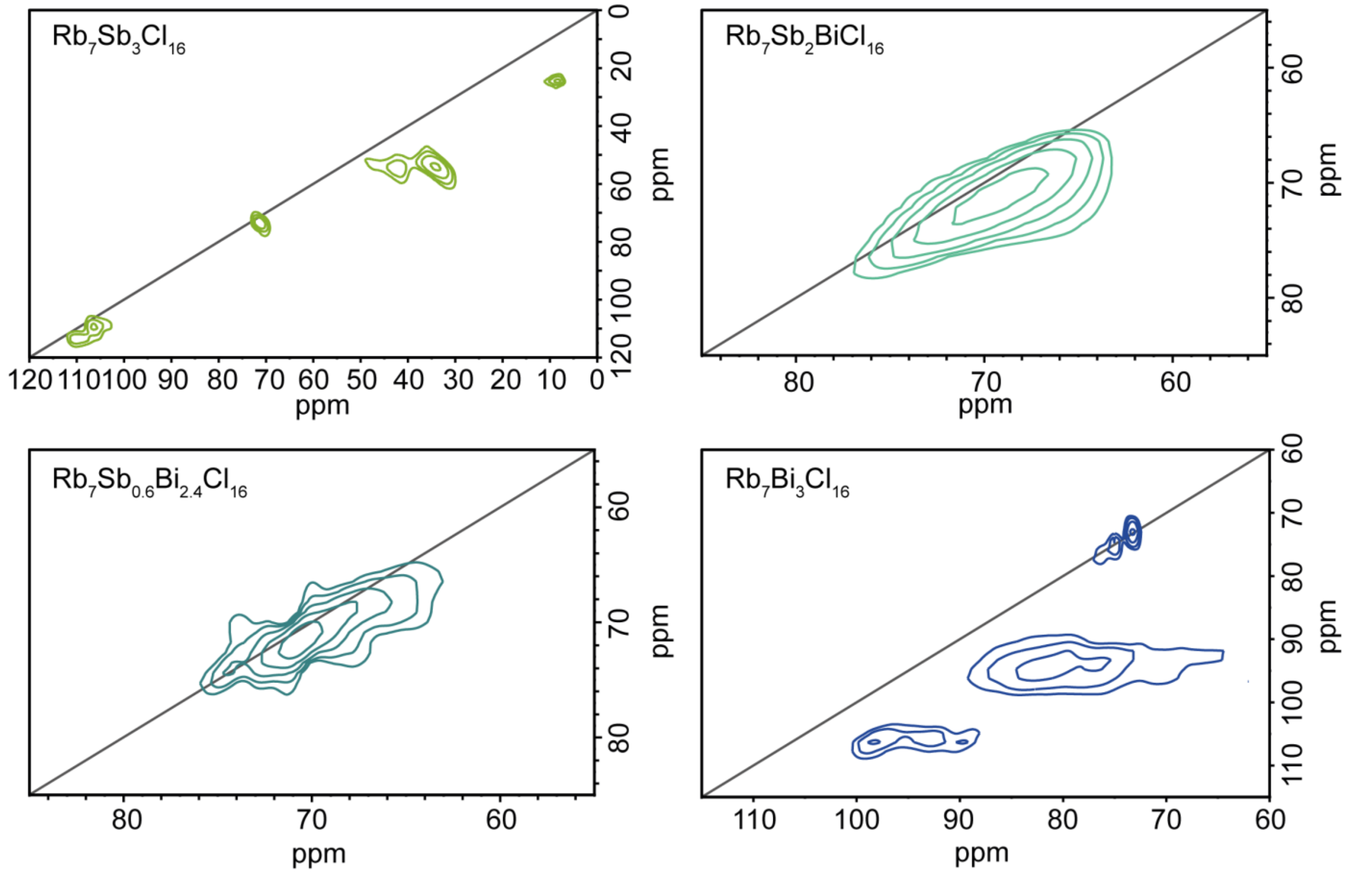

Figure S34. MQMAS ^{87}Rb NMR spectra for the $Rb_7Bi_{3-3x}Sb_{3x}Cl_{16}$ family.

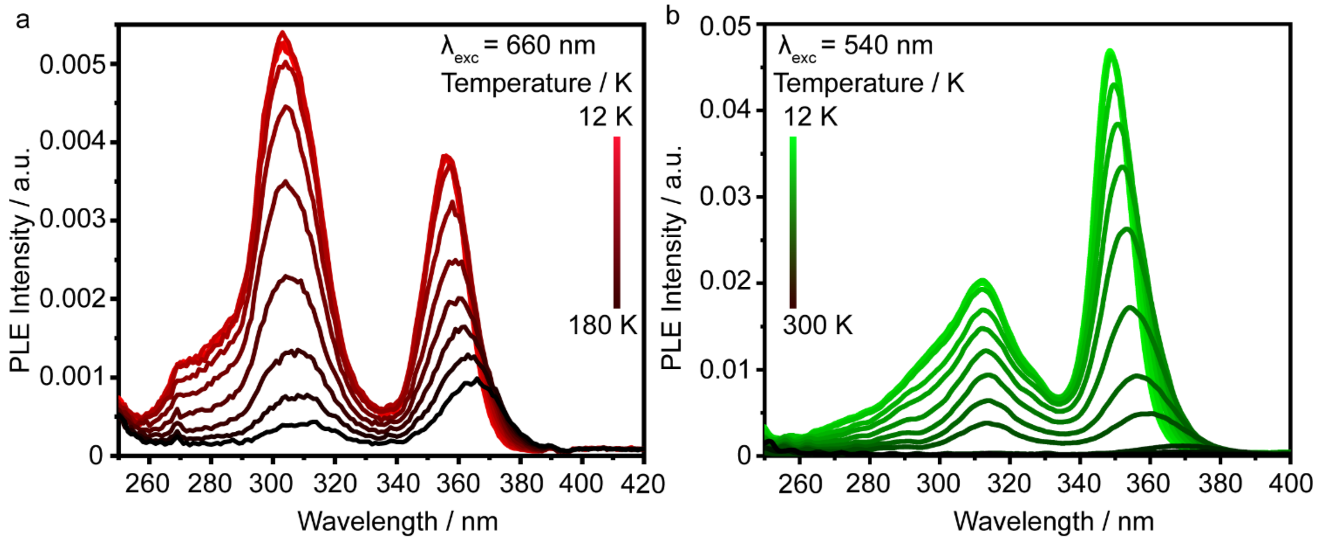

Figure S35. dT-PLE spectra for $Rb_7BiSb_2Cl_{16}$. Measured by detecting light emitted at a) 660 nm and b) 540 nm.

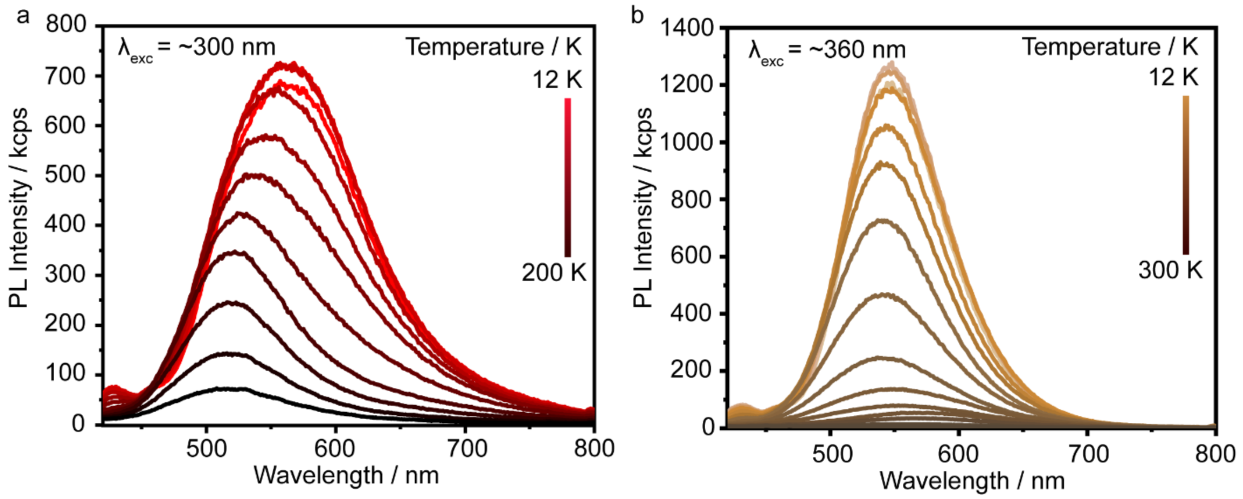

Figure 36. dT-PL spectra for $Rb_7BiSb_2Cl_{16}$. Measured by exciting at a) 300 nm and b) 360 nm.

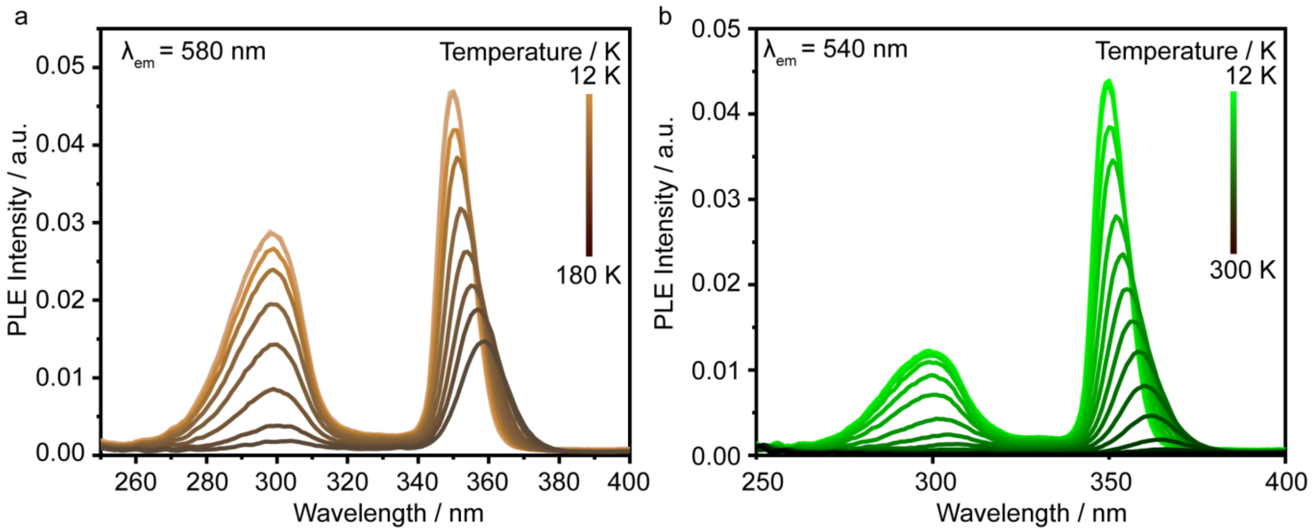

Figure S37. dT-PLE spectra for $Rb_7Bi_{2.6}Sb_{0.4}Cl_{16}$. Measured by detecting light emitted at a) 580 nm and b) 540 nm.

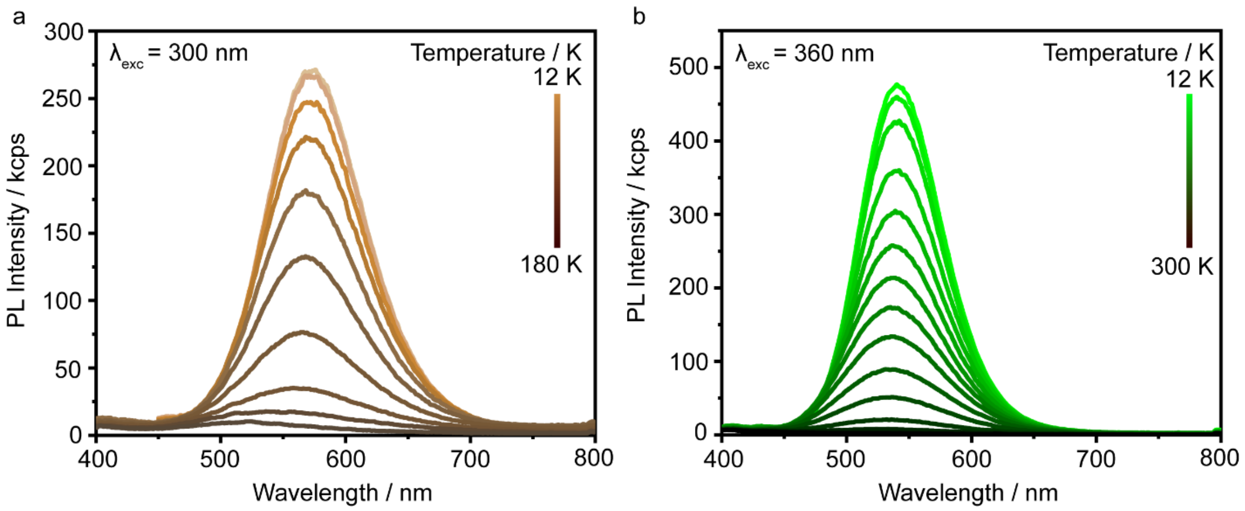

Figure S38. dT-PL spectra for $Rb_7Bi_{2.6}Sb_{0.4}Cl_{16}$. Measured by exciting at a) 300 nm and b) 360 nm.

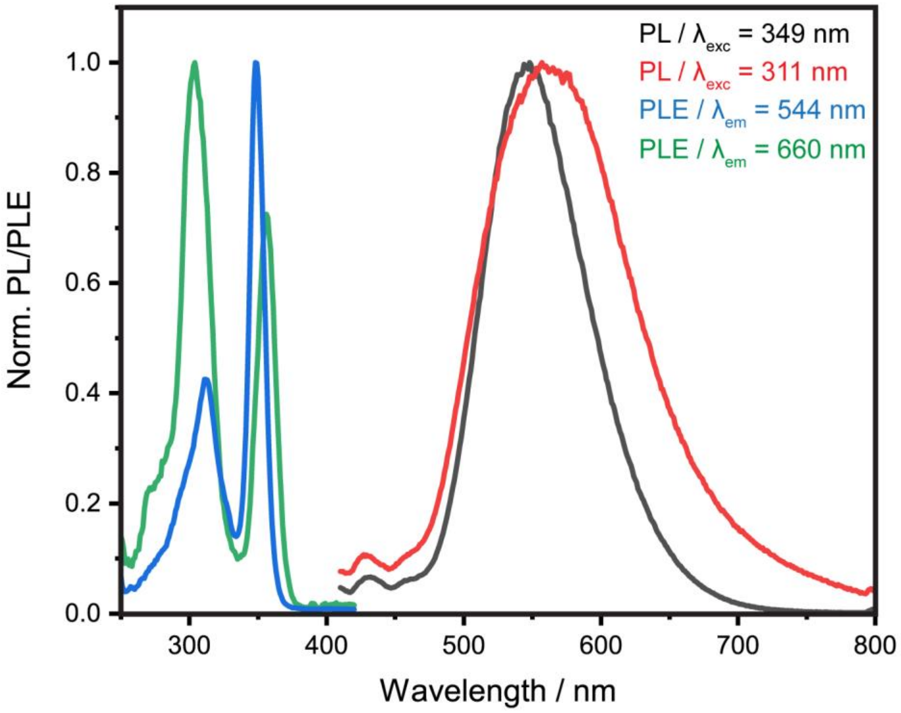

Figure S39. 12 K PL and PLE comparison for $Rb_7BiSb_2Cl_{16}$.

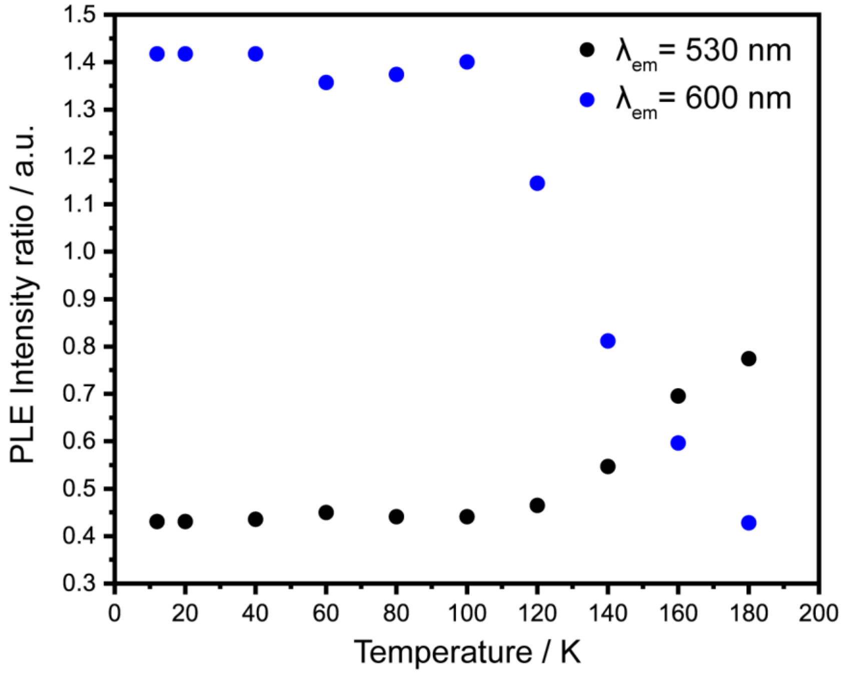

Figure S40. PLE peak intensity ratio as a function of temperature for $Rb_7BiSb_2Cl_{16}$.

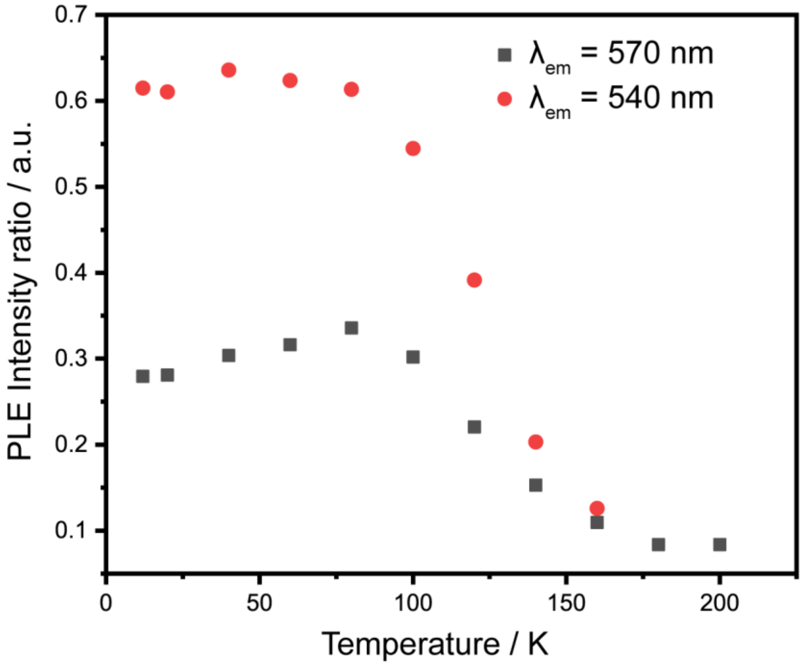

Figure S41. PLE peak intensity ratio as a function of temperature for $Rb_7Bi_{2.6}Sb_{0.4}Cl_{16}$.

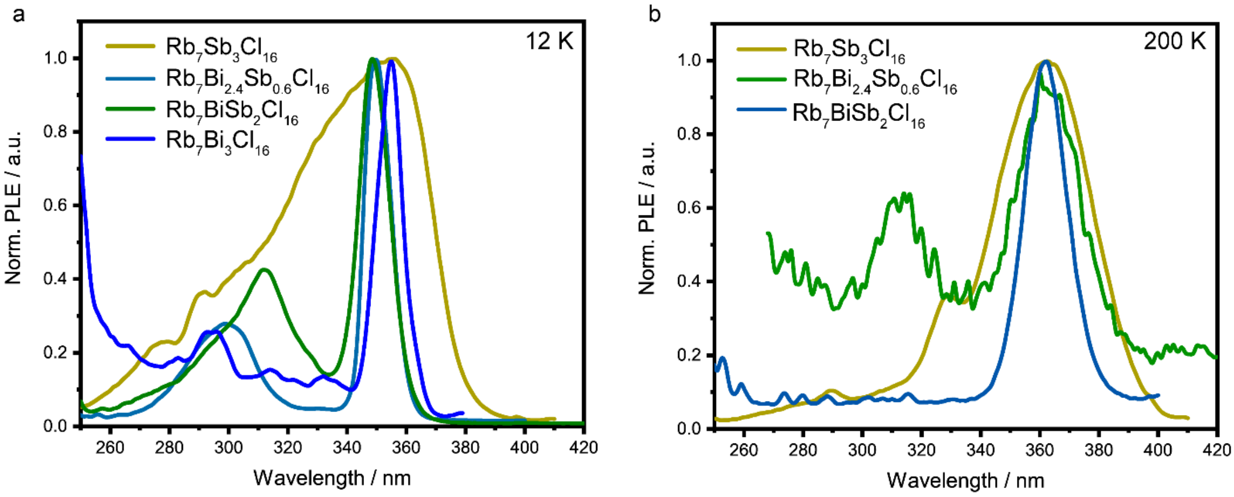

Figure S42. PLE comparisons for the $Rb_7Bi_{3-3x}Sb_{3x}Cl_{16}$ family at a) 12 K and b) 200 K.

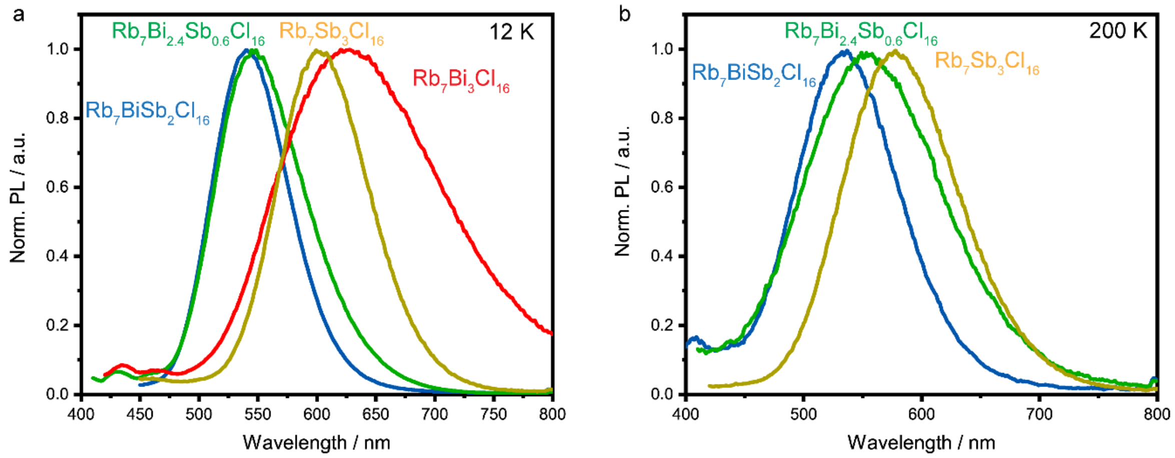

Figure S43. PL comparisons for the $Rb_7Bi_{3-3x}Sb_{3x}Cl_{16}$ family at a) 12 K and b) 200 K

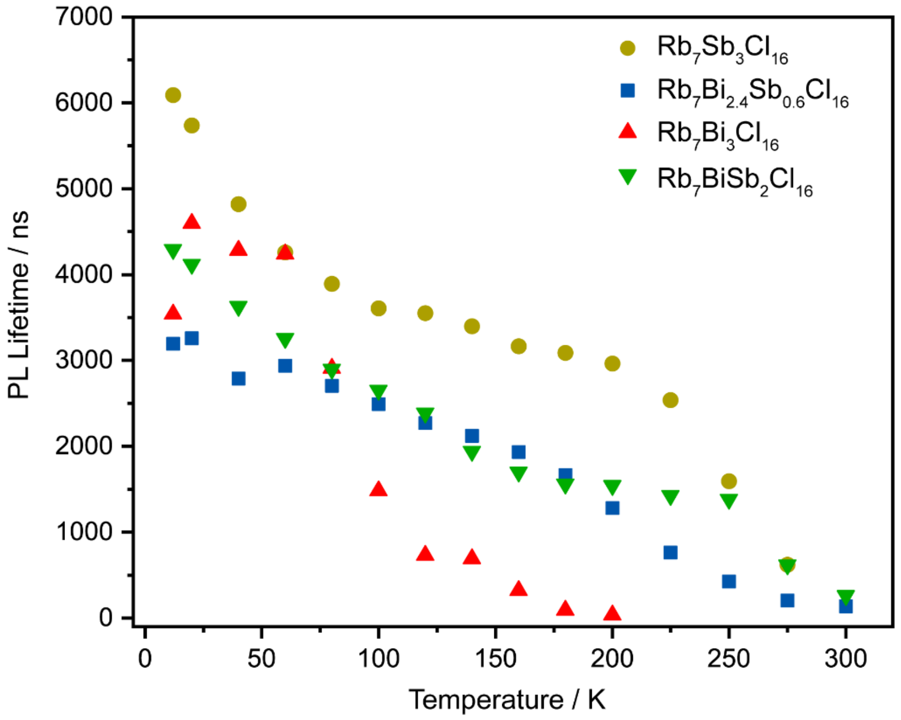

Figure S44. Average PL lifetimes with respect to temperature for the $Rb_7Bi_{3-3x}Sb_{3x}Cl_{16}$ family.

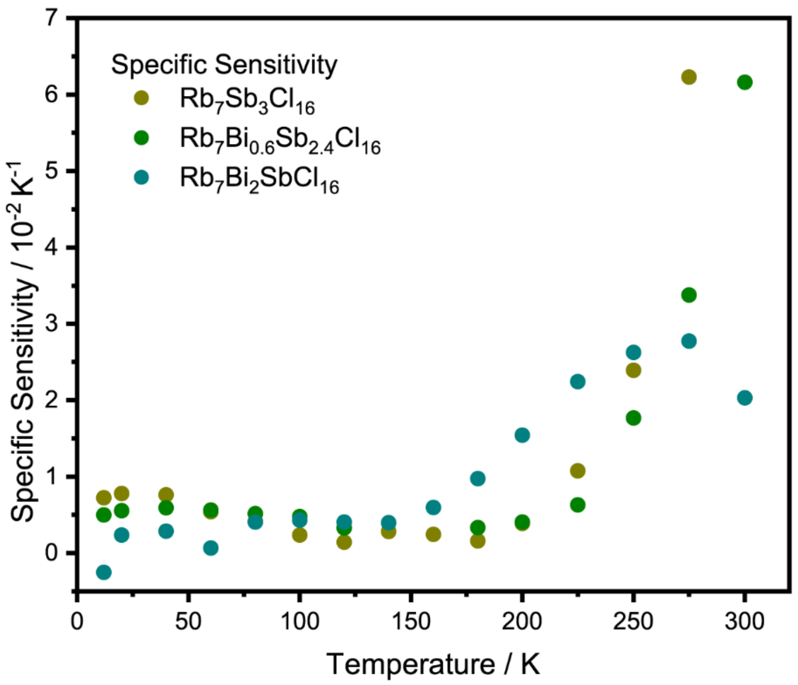

Figure S45. Specific sensitivity values for the $Rb_7Bi_{3-3x}Sb_{3x}Cl_{16}$ family.

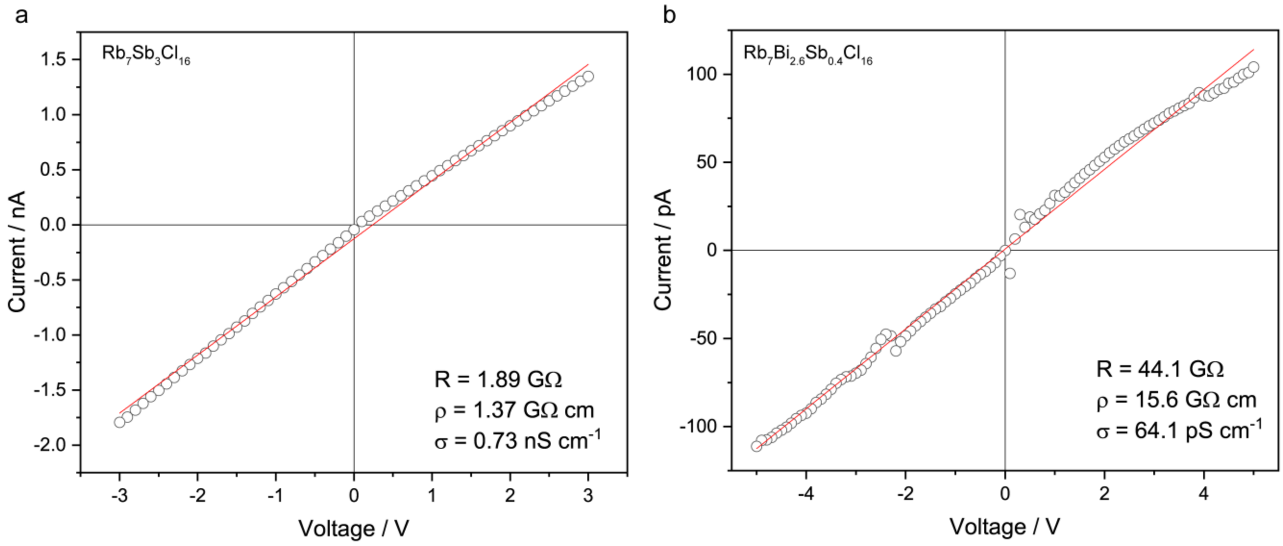

Figure S46. I-V curves for a) $Rb_7Sb_3Cl_{16}$ and b) $Rb_7Bi_{2.6}Sb_{0.4}Cl_{16}$.

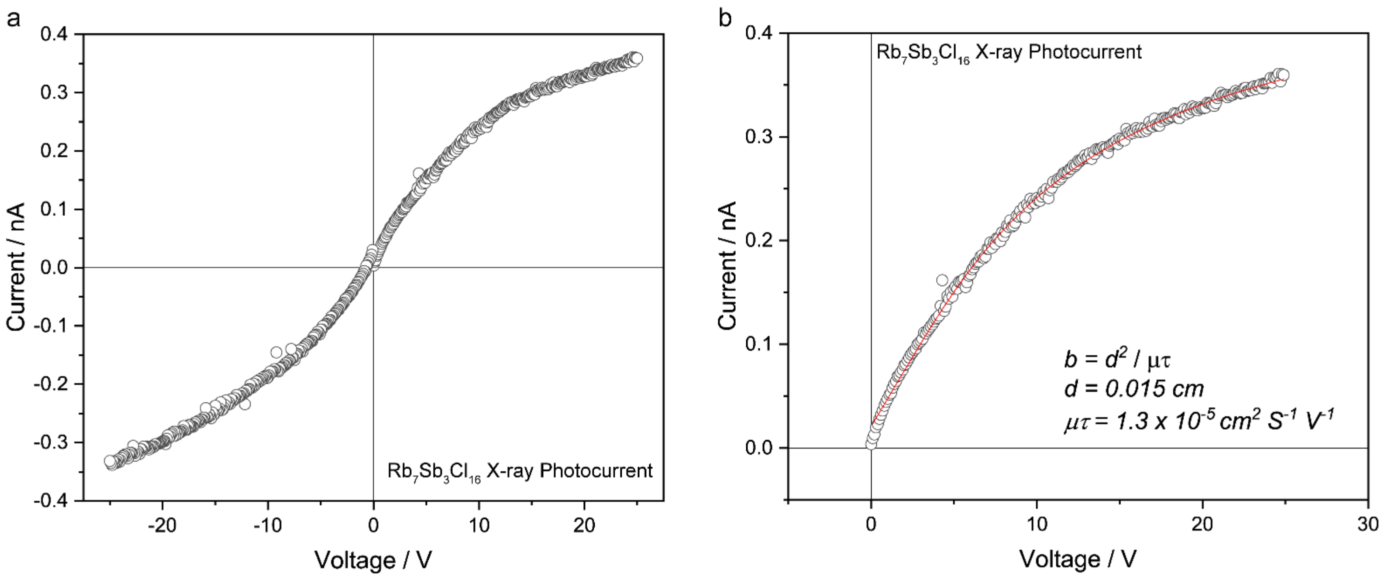

Figure S47. a) X-ray photoconductivity of $Rb_7Sb_3Cl_{16}$ and b) fitting with the Hecht equation to determine the μτ product.

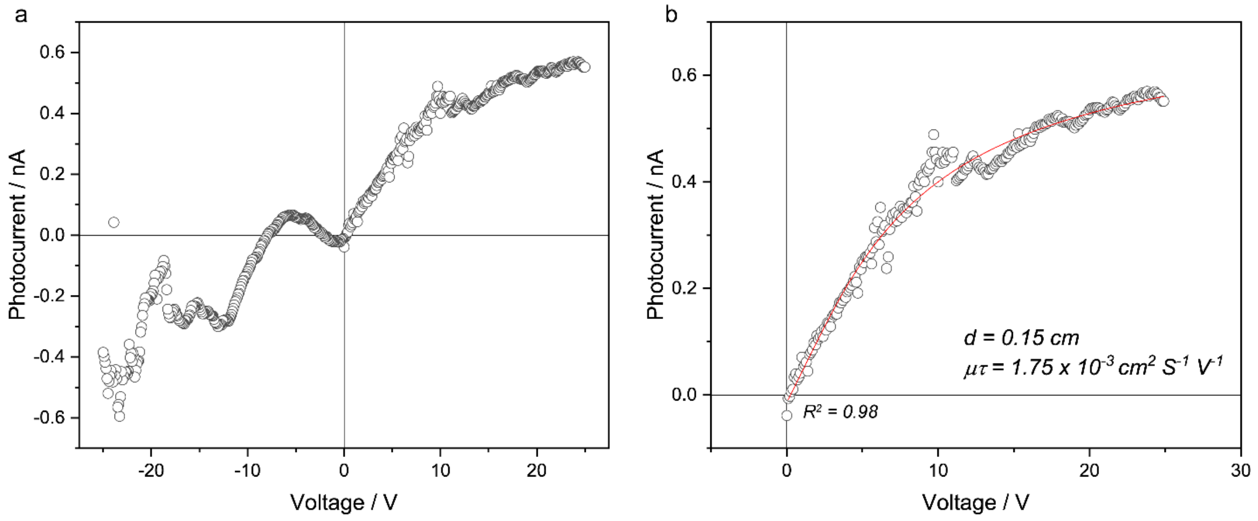

Figure S48. a) X-ray photoconductivity of $Rb_7Bi_{2.6}Sb_{0.4}Cl_{16}$ and b) fitting with the Hecht equation to determine the μτ product.

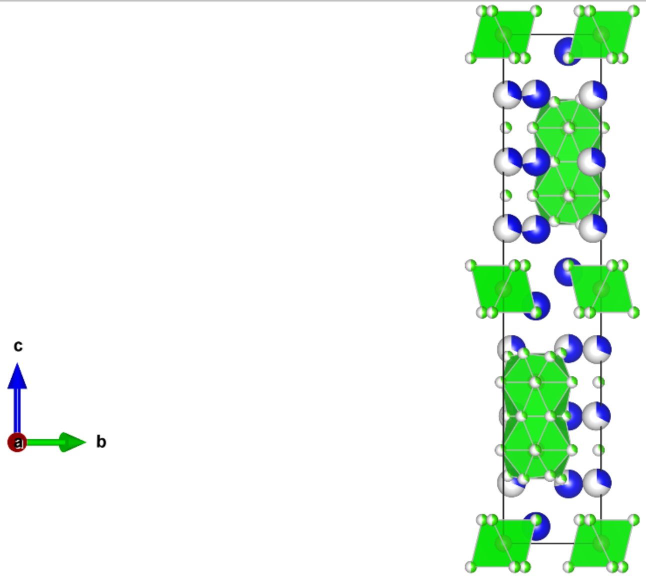

Figure S49. Structure of $K_7Sb_3Cl_{16}$ at 298 K.

Table S23. Crystal data and structure refinement for $K_7Sb_3Cl_{16}$ at 298.4(7) K.

Empirical formula	$K_7Sb_3Cl_{16}$
Formula weight	1206.35
Temperature	298.4(7) K
Wavelength	0.71073 Å
Crystal system	Hexagonal
Space group	*P*63/*mmc*
Unit cell dimensions	a = 7.3434(4) Å, α = 90°<br>b = 7.3434(4) Å, β = 90°<br>c = 33.143(2) Å, γ = 120°
Volume	1547.8(2) Å^3
Z	2
Density (calculated)	2.588 g/cm^3
Absorption coefficient	4.918 mm^{-1}
F(000)	1116
Crystal size	0.115 x 0.094 x 0.037 mm^3
θ range for data collection	2.458 to 33.335°
Index ranges	-6<=h<=9, -4<=k<=10, -33<=l<=50
Reflections collected	5651
Independent reflections	1161 [R_{int} = 0.0849]
Completeness to θ = 25.242°	99.8%
Refinement method	Full-matrix least-squares on F^2
Data / restraints / parameters	1161 / 0 / 66
Goodness-of-fit	1.171
Final R indices [I > 2σ(I)]	R_{obs} = 0.0673, wR_{obs} = 0.1104
R indices [all data]	R_{all} = 0.1118, wR_{all} = 0.1236
Largest diff. peak and hole	0.703 and -0.987 e·Å^{-3}

$R = \Sigma||F_o|-|F_c|| / \Sigma|F_o|$, $wR = \{\Sigma[w(|F_o|^2 - |F_c|^2)^2] / \Sigma[w(|F_o|^4)]\}^{1/2}$ and $w=1/[\sigma^2(Fo^2)+16.4487P]$ where $P=(Fo^2+2Fc^2)/3$

Table S24. Atomic coordinates (x10^4) and equivalent isotropic displacement parameters (Å^{2}x10^3) for $K_7Sb_3Cl_{16}$ at 298.4(7) K with estimated standard deviations in parentheses.

Label	x	y	z	Occupancy	U_{eq}*
Sb(1)	10000	10000	5000	1	32(1)
Sb(2)	3333.33	6666.67	6832(1)	1	27(1)
K(1)	6666.67	3333.33	4666(2)	1	78(2)
K(2)	6666.67	3333.33	6170(2)	0.722(16)	69(3)
K(3)	-415(7)	415(7)	6189(2)	0.313(6)	48(3)
K(4)	6666.67	3333.33	7500	0.72(2)	84(5)
K(5)	991(19)	495(10)	7500	0.323(9)	49(4)
Cl(1)	11147(9)	13336(8)	4538(2)	0.5	80(2)
Cl(2)	6994(8)	6739(9)	6833(2)	0.3333	40(2)
Cl(3)	4777(10)	5223(10)	6332(4)	0.3333	74(3)
Cl(4)	2061(9)	4122(18)	6268(3)	0.3333	72(3)
Cl(5)	4811(10)	5189(10)	7500	0.3333	60(4)
Cl(6)	1851(10)	3700(20)	7500	0.3333	66(4)

*U_{eq} is defined as one third of the trace of the orthogonalized U_{ij} tensor.

Table S25. Anisotropic displacement parameters (Å^{2}x10^3) for $K_7Sb_3Cl_{16}$ at 298.4(7) K with estimated standard deviations in parentheses.

Label	U_{11}	U_{22}	U_{33}	U_{12}	U_{13}	U_{23}
Sb(1)	29(1)	29(1)	38(1)	15(1)	0	0
Sb(2)	27(1)	27(1)	26(1)	14(1)	0	0
K(1)	67(2)	67(2)	100(4)	34(2)	0	0
K(2)	89(4)	89(4)	30(3)	44(2)	0	0
K(3)	39(3)	39(3)	38(3)	-3(3)	6(2)	-6(2)
K(4)	106(7)	106(7)	40(5)	53(4)	0	0
K(5)	74(8)	39(4)	46(6)	37(4)	0	0
Cl(1)	96(6)	46(2)	96(3)	35(3)	37(3)	23(3)
Cl(2)	32(3)	51(3)	40(2)	23(3)	2(2)	0(2)
Cl(3)	62(5)	62(5)	86(7)	22(5)	23(3)	-23(3)
Cl(4)	66(5)	75(7)	79(7)	38(4)	-24(3)	-49(6)
Cl(5)	39(5)	39(5)	105(11)	22(6)	0	0
Cl(6)	58(7)	28(7)	100(11)	14(3)	0	0

The anisotropic displacement factor exponent takes the form: $-2\pi^2[h^2a^{*2}U_{11} + ... + 2hka^*b^*U_{12}]$.

Table S26. Bond lengths [Å] for $K_7Sb_3Cl_{16}$ at 298.4(7) K with estimated standard deviations in parentheses.

Label	Distances
Sb(1)-Cl(1)	2.643(5)
Sb(2)-Cl(2)	2.662(5)
Sb(2)-Cl(3)	2.473(10)
Sb(2)-Cl(4)	2.473(9)
K(2)-Cl(2)	3.247(7)
K(2)-Cl(3)	2.464(11)
K(3)-Cl(4)	2.415(11)
K(4)-Cl(5)	2.360(13)
K(5)-Cl(5)	3.175(10)
K(5)-Cl(6)	2.110(13)
Cl(2)-Cl(3)	2.198(11)
Cl(3)-Cl(4)	1.750(8)
Cl(5)-Cl(6)	1.883(9)

Supplementary note S2. Structure solution of $K_7Sb_3Cl_{16}$.

The disordered *P*6$_3$*mmc* solution is an acceptable model for this structure, but ultimately not the most correct solution. There is insufficient data to satisfactorily apply a more ordered model to this data as was done for $Rb_7Bi_3Cl_{16}$. However, this data set shows the same features that led us to the adoption of the larger, ordered model as described in Supplementary note S1.

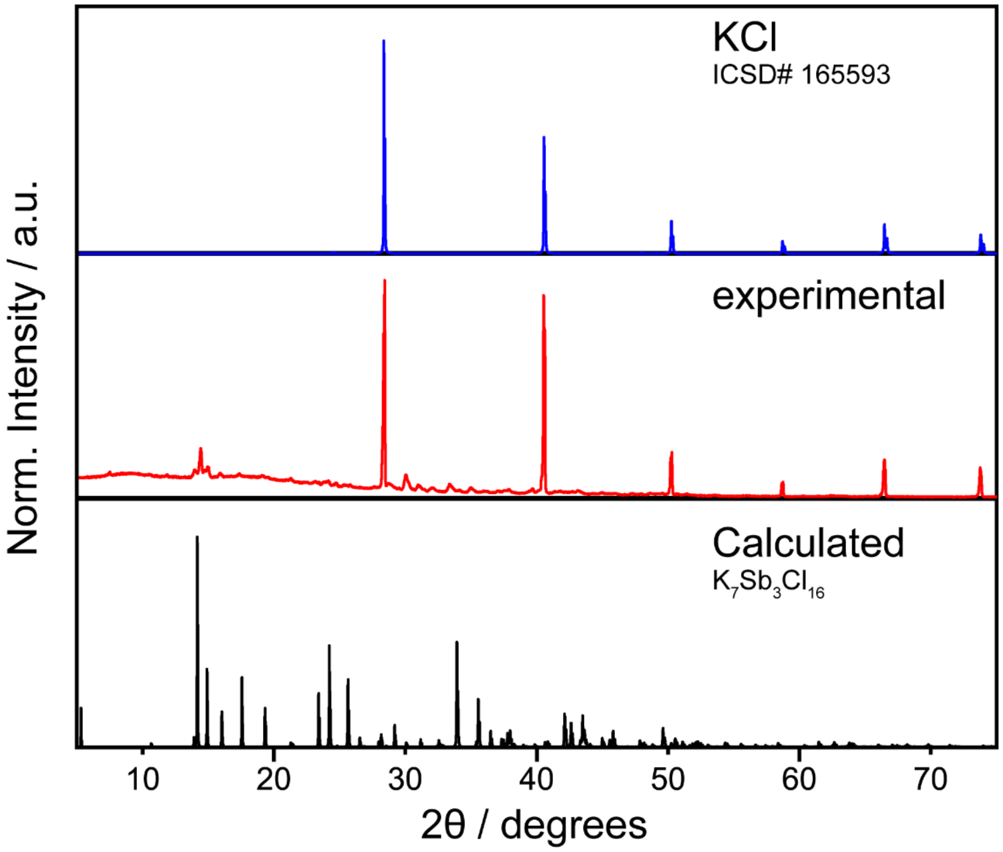

Figure S50. PXRD of $K_7Sb_3Cl_6$. The synthesis of $K_7Sb_3Cl_{16}$ typically resulted in the presence of large quantity of KCl; however, KCl is a non-emissive salt under UV excitation and therefore does not interfere in the investigation of optical properties such as photoluminescence.

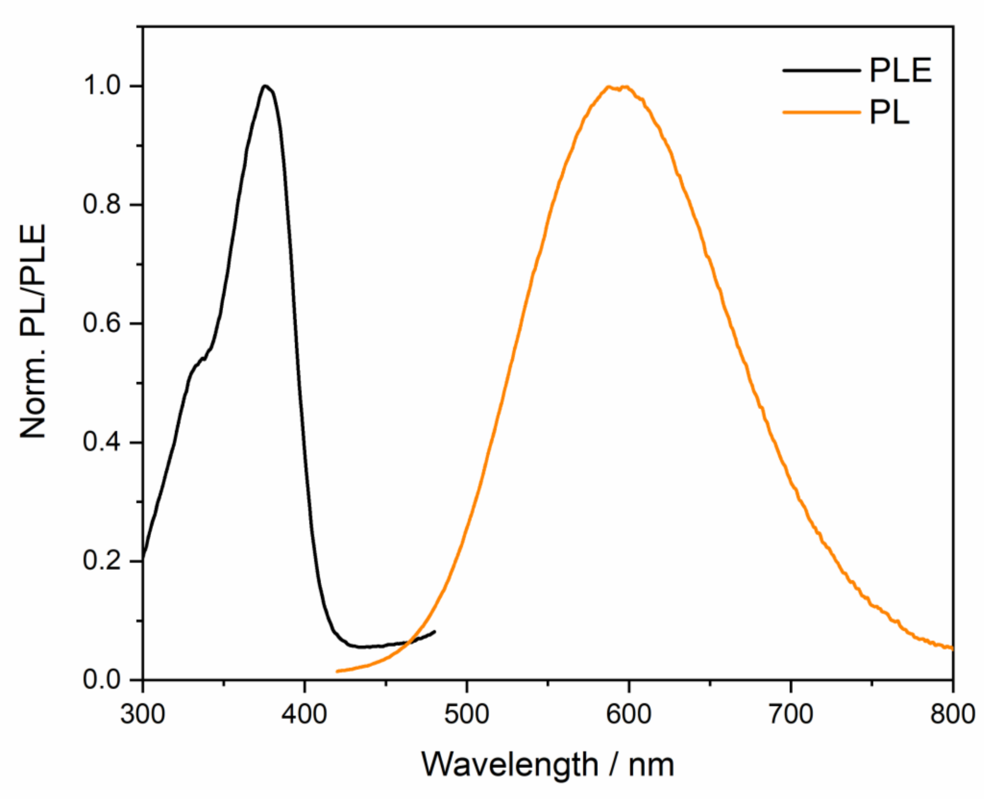

Figure S51. PL and PLE of $K_7Sb_3Cl_6$ at 298 K.

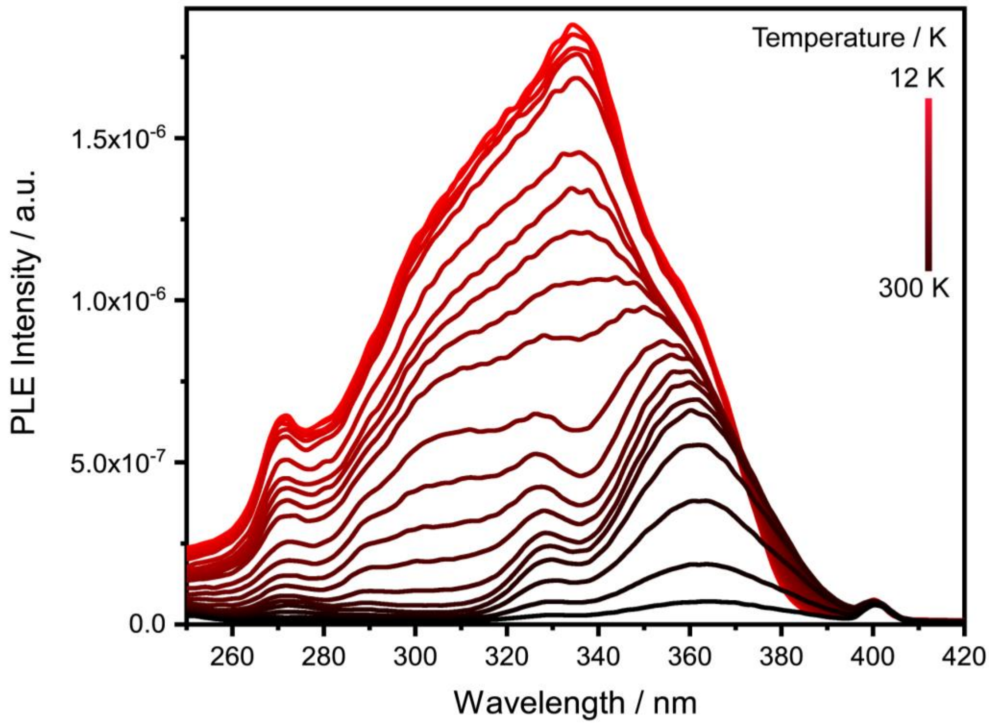

Figure S52. dT-PLE of $K_7Sb_3Cl_6$.

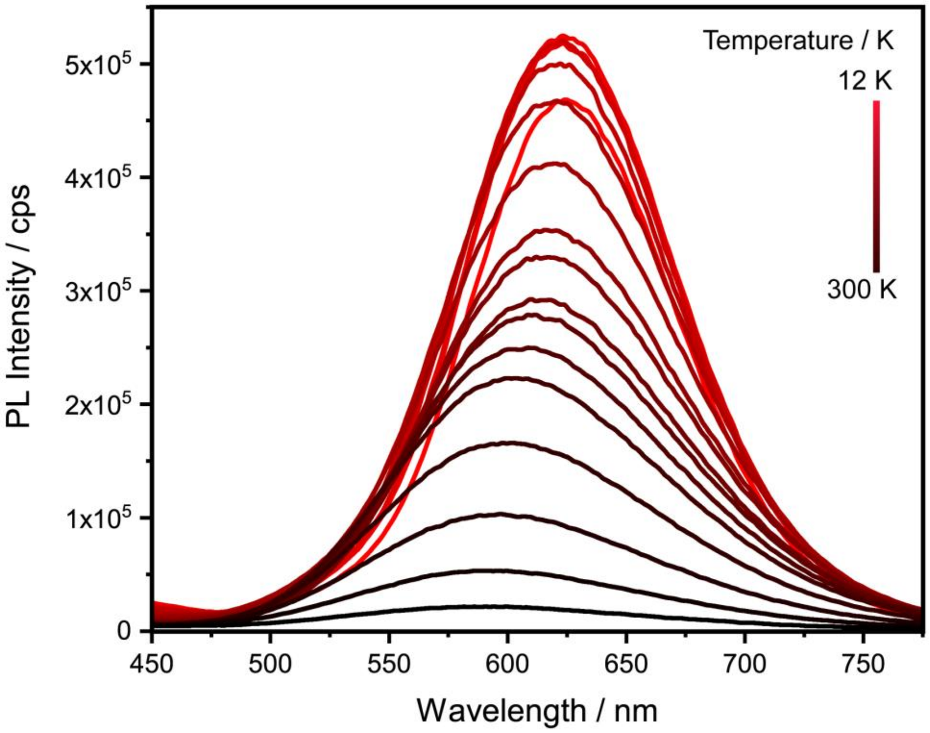

Figure S53. dT-PL of $K_7Sb_3Cl_6$.

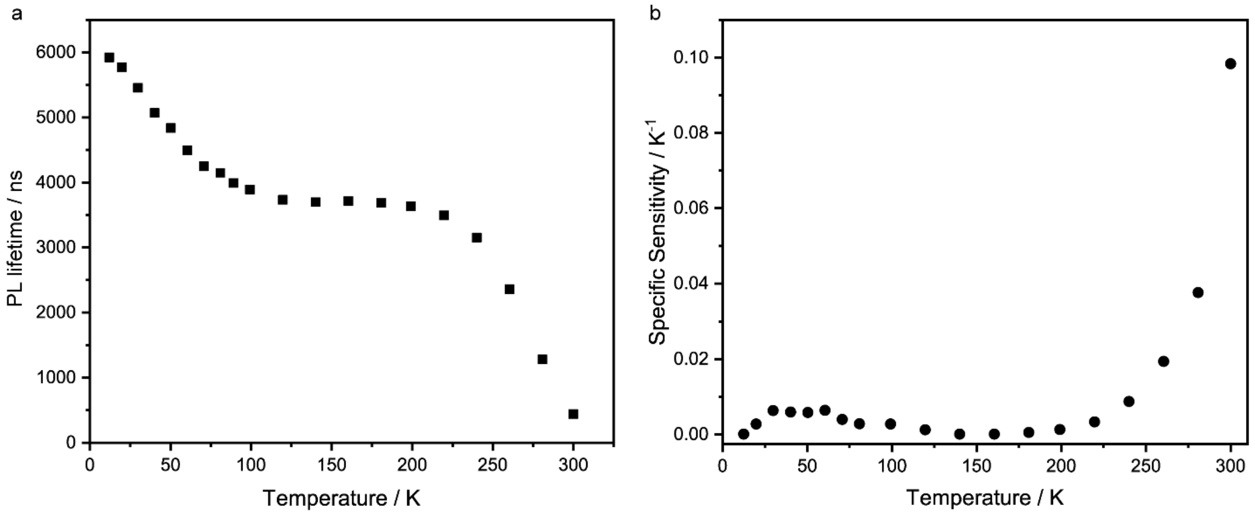

Figure S54. dT-TRPL (TRPL vs. temperature) of $K_7Sb_3Cl_6$. a) Average lifetime with temperature. b) specific sensitivity.

3. REFERENCES

[1] G. M. Sheldrick, *Acta Cryst.* **2008**, *64*, 112-122.
[2] O. V. Dolomanov, L. J. Bourhis, R. J. Gildea, J. A. K. Howard, H. Puschmann, *J. Appl. Cryst.* **2009**, *42*, 339-341.
[3] K. Momma; F. Izumi, *J. Appl. Cryst.* **2011**, *44*, 1272-1276.
